# Chinese species of egg-parasitoids of the genera *Oxyscelio* Kieffer, *Heptascelio* Kieffer and *Platyscelio* Kieffer (Hymenoptera: Platygastridae *s.l.*, Scelioninae)

**DOI:** 10.3897/BDJ.1.e987

**Published:** 2013-09-16

**Authors:** Norman F Johnson, Roger Burks, Andrew Austin, Xu Zaifu

**Affiliations:** † The Ohio State University, Columbus, OH, United States of America; ‡ The Ohio State University, Columbus, United States of America; § The University of Adelaide, Adelaide, Australia; | South China Agricultural University, Guangzhou, China

**Keywords:** parasitoid, Scelioninae, Orthoptera, Oriental region, Palearctic region

## Abstract

To date, the known Chinese fauna of egg-parasitoids of the genus *Oxyscelio* Kieffer encompasses two species from the mainland – *Oxyscelio doumao* Burks and *Oxyscelio nubbin* Burks. Here we record eighteen species of *Oxyscelio* from collections in mainland China: *Oxyscelio arvi* Burks, *Oxyscelio ceylonensis* (Dodd), *Oxyscelio convergens* Burks, *Oxyscelio cordis* Burks, *Oxyscelio crebritas* Burks, *Oxyscelio cuculli* Burks, *Oxyscelio dermatoglyphes* Burks, *Oxyscelio doumao* Burks, *Oxyscelio florus* Kononova, *Oxyscelio granorum* Burks, *Oxyscelio intermedietas* Burks, *Oxyscelio jugi* Burks, *Oxyscelio kramatos* Burks, *Oxyscelio longiventris* Burks, *Oxyscelio naraws* Kozlov & Lê, *Oxyscelio perpensus* Kononova, *Oxyscelio planocarinae* Burks, and *Oxyscelio striarum* Burks. *Oxyscelio* is primarily found in the tropics, and most of these species are shared with Taiwan and southeast Asia. Three species previously known only from Japan, *Oxyscelio arvi*, *Oxyscelio florus*, *Oxyscelio perpensus*, are shared. The Chinese species are recorded from Guangdong, Guangxi, Hainan, Hebei, Hunan, Shaanxi, Sichuan, Yunnan and Zhejiang as well as additional material from Taiwan. *Heptascelio hamatus* Masner & Johnson and *Platyscelio pulchricornis* Kieffer are both recorded from Hainan and Guangdong, as well as records of *Platyscelio pulchricornis* from Sarawak and Thailand.

## Introduction

The genus *Oxyscelio* Kieffer is an extraordinarily diverse group of egg parasitoids found in Australia, tropical Asia, Japan, and sub-Saharan Africa. In total, it comprises nearly two hundred species. The Indo-Malayan and Palearctic species have recently been revised (Burks et al. 2013), and in that work 90 species were recognized. *Heptascelio* Kieffer is a smaller genus, restricted to Africa and tropical Asia, and encompasses 18 species (Johnson et al. 2008). *Platyscelio* Kieffer, is even smaller, with only six species distributed through Africa, tropical Asia and Australia (Taekul et al. 2010). For all of these recent studies, the samples of specimens available from Asia were biased toward the faunas of southeast Asia and the Malay Archipelago, reflecting the intensive field work of our colleagues in the region, particularly D.C. Darling (Royal Ontario Museum) and M.J. Sharkey (University of Kentucky) and their in-country collaborators. In contrast, areas such as China, which would be expected to hold a rich fauna, had relatively few specimens available for inclusion. In this work, we report the results of the study of specimens in the Hymenopteran Collection of South China Agricultural University (Guangzhou) which begins to significantly address the lack of knowledge of the species of China.

## Materials and methods

A total of 191 specimens of the genera *Oxyscelio*, *Heptascelio* and *Platyscelio* from China were examined. The label data have been captured and are available through Hymenoptera Online (hol.osu.edu) and the Global Biodiversity Information Facility (www.gbif.org). Additionally, a DarwinCore Archive file as exported from Hymenoptera Online is included as a supplemental file to this paper. Species were identified using the keys of Burks et al. 2013, Johnson et al. 2008 and Taekul et al. 2010. In each taxon treatment the "Link to dynamic distribution map" generates a map using all of the data for that species that have been recorded in Hymenoptera Online.

## Taxon treatments

### Oxyscelio arvi

Burks, 2013

http://lsid.tdwg.org/urn:lsid:biosci.ohio-state.edu:osuc_concepts:275548

urn:lsid:zoobank.org:act:491CF086-1501-4C6C-BB0E-1A9588FECF4F

http://species-id.net/wiki/Oxyscelio_arvi

Oxyscelio arvi Burks et al. 2013: 16, 19, 46. Original description, keyed, placed in *florus* species group.

#### Materials

- **Type status:** Other material. **Occurrence:** catalogNumber: SCAU 2011000627; recordedBy: Zhang Hong-Ying; individualCount: 1; sex: female; lifeStage: adult; **Taxon:** taxonID: urn:lsid:biosci.ohio-state.edu:osuc_names:275548; scientificName: Oxyscelio arvi; **Location:** country: China; stateProvince: Zhejiang; locality: Mt Qingliangfeng; locationRemarks: label transliteration: "Zhejiang, Qingliangfeng, 2005.08.09, Zhang Hongying"; [浙江清凉峰 2005.08.09 张红英]; decimalLatitude: 30.0703; decimalLongitude: 118.8944; georeferenceProtocol: Google Earth; georeferenceRemarks: GPS coords. adjusted to place within Zhejiang Prov.; **Identification:** identifiedBy: Norman F. Johnson; dateIdentified: 2012; **Event:** eventID: urn:lsid:biosci.ohio-state.edu:osuc_occurrences:SCAU__2011000627; samplingProtocol: none specified; eventDate: 2005-08-05; **Record Level:** modified: 2013-07-17T11:03:59Z; language: en; collectionID: urn:lsid:biocol.org:col:34252; collectionCode: Insects; basisOfRecord: PreservedSpecimen; source: http://hol.osu.edu/spmInfo.html?id=SCAU%202011000627
- **Type status:** Other material. **Occurrence:** catalogNumber: SCAU 2011000626; recordedBy: Zhang Hong-Ying; individualCount: 1; sex: female; lifeStage: adult; **Taxon:** taxonID: urn:lsid:biosci.ohio-state.edu:osuc_names:275548; scientificName: Oxyscelio arvi; **Location:** country: China; stateProvince: Zhejiang; locality: Mt Qingliangfeng; locationRemarks: label transliteration: "Zhejiang, Qingliangfeng, 2005.08.09, Zhang Hongying"; [浙江清凉峰 2005.08.09 张红英]; decimalLatitude: 30.0703; decimalLongitude: 118.8944; georeferenceProtocol: Google Earth; georeferenceRemarks: GPS coords. adjusted to place within Zhejiang Prov.; **Identification:** identifiedBy: Norman F. Johnson; dateIdentified: 2012; **Event:** eventID: urn:lsid:biosci.ohio-state.edu:osuc_occurrences:SCAU__2011000626; samplingProtocol: none specified; eventDate: 2005-08-05; **Record Level:** modified: 2013-07-17T11:03:59Z; language: en; collectionID: urn:lsid:biocol.org:col:34252; collectionCode: Insects; basisOfRecord: PreservedSpecimen; source: http://hol.osu.edu/spmInfo.html?id=SCAU%202011000626
- **Type status:** Other material. **Occurrence:** catalogNumber: SCAU 2011000467; recordedBy: Shi Min; individualCount: 1; sex: female; lifeStage: adult; **Taxon:** taxonID: urn:lsid:biosci.ohio-state.edu:osuc_names:275548; scientificName: Oxyscelio arvi; **Location:** country: China; stateProvince: Zhejiang; locality: Mt Qingliangfeng; locationRemarks: label transliteration: "Zhejiang, Qingliangfeng, 2005.08.09, Shi Min"; [浙江清凉峰 2005.08.09 时敏]; decimalLatitude: 30.0703; decimalLongitude: 118.8944; georeferenceProtocol: Google Earth; georeferenceRemarks: GPS coords. adjusted to place within Zhejiang Prov.; **Identification:** identifiedBy: Norman F. Johnson; dateIdentified: 2012; **Event:** eventID: urn:lsid:biosci.ohio-state.edu:osuc_occurrences:SCAU__2011000467; samplingProtocol: none specified; eventDate: 2005-08-05; **Record Level:** modified: 2013-07-17T11:03:53Z; language: en; collectionID: urn:lsid:biocol.org:col:34252; collectionCode: Insects; basisOfRecord: PreservedSpecimen; source: http://hol.osu.edu/spmInfo.html?id=SCAU%202011000467
- **Type status:** Other material. **Occurrence:** catalogNumber: SCAU 2011000631; recordedBy: Zhang Hong-Ying; individualCount: 1; sex: female; lifeStage: adult; **Taxon:** taxonID: urn:lsid:biosci.ohio-state.edu:osuc_names:275548; scientificName: Oxyscelio arvi; **Location:** country: China; stateProvince: Zhejiang; locality: Mt Qingliangfeng; locationRemarks: label transliteration: "Zhejiang, Qingliangfeng, 2005.08.09, Zhang Hongying"; [浙江清凉峰 2005.08.09 张红英]; decimalLatitude: 30.0703; decimalLongitude: 118.8944; georeferenceProtocol: Google Earth; georeferenceRemarks: GPS coords. adjusted to place within Zhejiang Prov.; **Identification:** identifiedBy: Norman F. Johnson; dateIdentified: 2012; **Event:** eventID: urn:lsid:biosci.ohio-state.edu:osuc_occurrences:SCAU__2011000631; samplingProtocol: none specified; eventDate: 2005-08-05; **Record Level:** modified: 2013-07-17T11:04:00Z; language: en; collectionID: urn:lsid:biocol.org:col:34252; collectionCode: Insects; basisOfRecord: PreservedSpecimen; source: http://hol.osu.edu/spmInfo.html?id=SCAU%202011000631
- **Type status:** Other material. **Occurrence:** catalogNumber: SCAU 2011000633; recordedBy: Zhang Hong-Ying; individualCount: 1; sex: female; lifeStage: adult; **Taxon:** taxonID: urn:lsid:biosci.ohio-state.edu:osuc_names:275548; scientificName: Oxyscelio arvi; **Location:** country: China; stateProvince: Zhejiang; locality: Mt Qingliangfeng; locationRemarks: label transliteration: "Zhejiang, Qingliangfeng, 2005.08.09, Zhang Hongying"; [浙江清凉峰 2005.08.09 张红英]; decimalLatitude: 30.0703; decimalLongitude: 118.8944; georeferenceProtocol: Google Earth; georeferenceRemarks: GPS coords. adjusted to place within Zhejiang Prov.; **Identification:** identifiedBy: Norman F. Johnson; dateIdentified: 2012; **Event:** eventID: urn:lsid:biosci.ohio-state.edu:osuc_occurrences:SCAU__2011000633; samplingProtocol: none specified; eventDate: 2005-08-05; **Record Level:** modified: 2013-07-17T11:04:01Z; language: en; collectionID: urn:lsid:biocol.org:col:34252; collectionCode: Insects; basisOfRecord: PreservedSpecimen; source: http://hol.osu.edu/spmInfo.html?id=SCAU%202011000633
- **Type status:** Other material. **Occurrence:** catalogNumber: SCAU 2011000449; recordedBy: Zhang Hong-Ying; individualCount: 1; sex: female; lifeStage: adult; **Taxon:** taxonID: urn:lsid:biosci.ohio-state.edu:osuc_names:275548; scientificName: Oxyscelio arvi; **Location:** country: China; stateProvince: Sichuan; locality: Mt Qingliangfeng; locationRemarks: label transliteration: "Sichuan, Emeishan, 2006.08.01, Zhang Hongying"; [四川峨眉山 2006.08.01, 张红英]; decimalLatitude: 29.5667; decimalLongitude: 103.4333; georeferenceProtocol: GPS; **Identification:** identifiedBy: Norman F. Johnson; dateIdentified: 2012; **Event:** eventID: urn:lsid:biosci.ohio-state.edu:osuc_occurrences:SCAU__2011000449; samplingProtocol: none specified; eventDate: 2006-08-01; **Record Level:** modified: 2013-07-17T11:03:52Z; language: en; collectionID: urn:lsid:biocol.org:col:34252; collectionCode: Insects; basisOfRecord: PreservedSpecimen; source: http://hol.osu.edu/spmInfo.html?id=SCAU%202011000449
- **Type status:** Other material. **Occurrence:** catalogNumber: SCAU 2010100391; recordedBy: Chen Hua-Yan; individualCount: 1; sex: female; lifeStage: adult; **Taxon:** taxonID: urn:lsid:biosci.ohio-state.edu:osuc_names:275548; scientificName: Oxyscelio arvi; **Location:** country: China; stateProvince: Guangdong; county: Zhaoqing; locality: Xiwang Valley; locationRemarks: label transliteration: "Guangdong, Zhaoqing xiwang gu (valley), 2010.08.2-6, Chen Huayan"; [广东肇庆希望谷 2010.08.2-6, 陈华燕]; decimalLatitude: 23.2167; decimalLongitude: 112.5167; georeferenceProtocol: GPS; **Identification:** identifiedBy: Norman F. Johnson; dateIdentified: 2012; **Event:** eventID: urn:lsid:biosci.ohio-state.edu:osuc_occurrences:SCAU__2010100391; samplingProtocol: none specified; eventDate: 2010-08-02/06; **Record Level:** modified: 2013-07-17T11:03:26Z; language: en; collectionID: urn:lsid:biocol.org:col:34252; collectionCode: Insects; basisOfRecord: PreservedSpecimen; source: http://hol.osu.edu/spmInfo.html?id=SCAU%202010100391

#### Distribution

This species previously was known only from Japan, on the island of Honshu. It is widespread in China, occurring in Sichuan, Zhejiang and Guangdong Provinces. Link to dynamic distribution map: http://hol.osu.edu/map-large.html?id=275548

### Oxyscelio ceylonensis

(Dodd, 1920)

http://lsid.tdwg.org/urn:lsid:biosci.ohio-state.edu:osuc_concepts:5010

urn:lsid:zoobank.org:act:FA70AF9F-816F-497E-BD85-674997D2543B

http://species-id.net/wiki/Oxyscelio_ceylonensis

Sceliomorpha ceylonensis Dodd 1920: 349. Original description.

#### Materials

- **Type status:** Other material. **Occurrence:** catalogNumber: SCAU 2011000651; recordedBy: Wu Qiong; individualCount: 1; sex: female; lifeStage: adult; **Taxon:** taxonID: urn:lsid:biosci.ohio-state.edu:osuc_names:5010; scientificName: Oxyscelio ceylonensis; **Location:** country: China; stateProvince: Zhejiang; county: Hangzhou; locality: West Lake; locationRemarks: label transliteration: "Zhejiang, Hangzhou, West Lake, 2003.08.25,Wu Qiong"; [浙江杭州西湖 2003.08.25 吴琼]; decimalLatitude: 30.25; decimalLongitude: 120.1167; georeferenceProtocol: GPS; **Identification:** identifiedBy: Norman F. Johnson; dateIdentified: 2012; **Event:** eventID: urn:lsid:biosci.ohio-state.edu:osuc_occurrences:SCAU__2011000651; samplingProtocol: none specified; eventDate: 2003-08-25; **Record Level:** modified: 2013-07-17T11:04:02Z; language: en; collectionID: urn:lsid:biocol.org:col:34252; collectionCode: Insects; basisOfRecord: PreservedSpecimen; source: http://hol.osu.edu/spmInfo.html?id=SCAU%202011000651
- **Type status:** Other material. **Occurrence:** catalogNumber: SCAU 20095891; recordedBy: Ma Juan-Juan; individualCount: 1; sex: male; lifeStage: adult; **Taxon:** taxonID: urn:lsid:biosci.ohio-state.edu:osuc_names:5010; scientificName: Oxyscelio ceylonensis; **Location:** country: China; stateProvince: Guangdong; locality: Nanling National Nature Reserve; locationRemarks: label transliteration: "Guangdong, Nanling, 2004.07.09-18, Ma Juanjuan"; [广东南岭 2004.07.09-18, 马娟娟]; decimalLatitude: 24.9; decimalLongitude: 113; georeferenceProtocol: GPS; **Identification:** identifiedBy: Norman F. Johnson; dateIdentified: 2012; **Event:** eventID: urn:lsid:biosci.ohio-state.edu:osuc_occurrences:SCAU__20095891; samplingProtocol: none specified; eventDate: 2004-07-09/18; **Record Level:** modified: 2013-07-17T11:03:22Z; language: en; collectionID: urn:lsid:biocol.org:col:34252; collectionCode: Insects; basisOfRecord: PreservedSpecimen; source: http://hol.osu.edu/spmInfo.html?id=SCAU%2020095891
- **Type status:** Other material. **Occurrence:** catalogNumber: SCAU 200905875; recordedBy: Ma Juan-Juan; individualCount: 1; sex: female; lifeStage: adult; **Taxon:** taxonID: urn:lsid:biosci.ohio-state.edu:osuc_names:5010; scientificName: Oxyscelio ceylonensis; **Location:** country: China; stateProvince: Guangdong; locality: Nanling National Nature Reserve; locationRemarks: label transliteration: "Guangdong, Nan ling, 2004.07.09-18, Ma Juanjuan"; [广东南岭 2004.07.09-18, 马娟娟]; decimalLatitude: 24.9; decimalLongitude: 113; georeferenceProtocol: GPS; **Identification:** identifiedBy: Norman F. Johnson; dateIdentified: 2012; **Event:** eventID: urn:lsid:biosci.ohio-state.edu:osuc_occurrences:SCAU__200905875; samplingProtocol: none specified; eventDate: 2004-07-09/18; **Record Level:** modified: 2013-07-17T11:03:16Z; language: en; collectionID: urn:lsid:biocol.org:col:34252; collectionCode: Insects; basisOfRecord: PreservedSpecimen; source: http://hol.osu.edu/spmInfo.html?id=SCAU%20200905875

#### Distribution

This is a widespread species in south and southeast Asia, extending from India (Karnataka and Andhra Pradesh), Sri Lanka and Ceylon, east to Thailand, Vietnam, and Sabah. It has previously been recorded from Guangdong in China. The new data are additional records from Nanling Reserve in Guangdong and Hangzhou in Zhejiang Province of eastern China. Link to dynamic distribution map: http://hol.osu.edu/map-large.html?id=5010

### Oxyscelio convergens

Burks, 2013

http://lsid.tdwg.org/urn:lsid:biosci.ohio-state.edu:osuc_concepts:275500

urn:lsid:zoobank.org:act:E03A3DFC-3859-4097-9D95-508F16CF1C04

http://species-id.net/wiki/Oxyscelio_convergens

Oxyscelio convergens Burks et al. 2013

#### Materials

- **Type status:** Other material. **Occurrence:** catalogNumber: SCAU 2011000646; recordedBy: Shi Min; individualCount: 1; sex: female; lifeStage: adult; **Taxon:** taxonID: urn:lsid:biosci.ohio-state.edu:osuc_names:275500; scientificName: Oxyscelio convergens; **Location:** country: China; stateProvince: Zhejiang; locality: Gutianshan National Nature Reserve, Zhejiang Prov, China; locationRemarks: label transliteration: "Zhejiang, Gutianshan, 2005.07.03, Shi Min"; [浙江古田山, 2005.07.03, 时敏]; decimalLatitude: 29.2636; decimalLongitude: 118.1339; georeferenceProtocol: GEOnet; **Identification:** identifiedBy: Norman F. Johnson; dateIdentified: 2012; **Event:** eventID: urn:lsid:biosci.ohio-state.edu:osuc_occurrences:SCAU__2011000646; samplingProtocol: none specified; eventDate: 2005-07-03; **Record Level:** modified: 2013-07-17T11:04:01Z; language: en; collectionID: urn:lsid:biocol.org:col:34252; collectionCode: Insects; basisOfRecord: PreservedSpecimen; source: http://hol.osu.edu/spmInfo.html?id=SCAU%202011000646
- **Type status:** Other material. **Occurrence:** catalogNumber: SCAU 2011000621; recordedBy: Zhang Hong-Ying; individualCount: 1; sex: female; lifeStage: adult; **Taxon:** taxonID: urn:lsid:biosci.ohio-state.edu:osuc_names:275500; scientificName: Oxyscelio convergens; **Location:** country: China; stateProvince: Zhejiang; locality: Mt Qingliangfeng, Zhejiang Prov., China; locationRemarks: label transliteration: "Zhejiang, Qingliangfeng, 2005.08.09, Zhang Hongying"; [浙江清凉峰 2005.08.09 张红英]; decimalLatitude: 30.0703; decimalLongitude: 118.8944; georeferenceProtocol: Google Earth; georeferenceRemarks: GPS coords. adjusted to place within Zhejiang Prov.; **Identification:** identifiedBy: Norman F. Johnson; dateIdentified: 2012; **Event:** eventID: urn:lsid:biosci.ohio-state.edu:osuc_occurrences:SCAU__2011000621; samplingProtocol: none specified; eventDate: 2005-08-09; **Record Level:** modified: 2013-07-17T11:03:59Z; language: en; collectionID: urn:lsid:biocol.org:col:34252; collectionCode: Insects; basisOfRecord: PreservedSpecimen; source: http://hol.osu.edu/spmInfo.html?id=SCAU%202011000621
- **Type status:** Other material. **Occurrence:** catalogNumber: SCAU 2011000620; recordedBy: Zhang Hong-Ying; individualCount: 1; sex: female; lifeStage: adult; **Taxon:** taxonID: urn:lsid:biosci.ohio-state.edu:osuc_names:275500; scientificName: Oxyscelio convergens; **Location:** country: China; stateProvince: Zhejiang; locality: Mt Qingliangfeng, Zhejiang Prov., China; locationRemarks: label transliteration: "Zhejiang, Qingliangfeng, 2005.08.09, Zhang Hongying"; [浙江清凉峰 2005.08.09 张红英]; decimalLatitude: 30.0703; decimalLongitude: 118.8944; georeferenceProtocol: Google Earth; georeferenceRemarks: GPS coords. adjusted to place within Zhejiang Prov.; **Identification:** identifiedBy: Norman F. Johnson; dateIdentified: 2012; **Event:** eventID: urn:lsid:biosci.ohio-state.edu:osuc_occurrences:SCAU__2011000620; samplingProtocol: none specified; eventDate: 2005-08-09; **Record Level:** modified: 2013-07-17T11:03:59Z; language: en; collectionID: urn:lsid:biocol.org:col:34252; collectionCode: Insects; basisOfRecord: PreservedSpecimen; source: http://hol.osu.edu/spmInfo.html?id=SCAU%202011000620
- **Type status:** Other material. **Occurrence:** catalogNumber: SCAU 2011000619; recordedBy: Zhang Hong-Ying; individualCount: 1; sex: female; lifeStage: adult; **Taxon:** taxonID: urn:lsid:biosci.ohio-state.edu:osuc_names:275500; scientificName: Oxyscelio convergens; **Location:** country: China; stateProvince: Zhejiang; locality: Mt Qingliangfeng, Zhejiang Prov., China; locationRemarks: label transliteration: "Zhejiang, Qingliangfeng, 2005.08.09, Zhang Hongying"; [浙江清凉峰 2005.08.09 张红英]; decimalLatitude: 30.0703; decimalLongitude: 118.8944; georeferenceProtocol: Google Earth; georeferenceRemarks: GPS coords. adjusted to place within Zhejiang Prov.; **Identification:** identifiedBy: Norman F. Johnson; dateIdentified: 2012; **Event:** eventID: urn:lsid:biosci.ohio-state.edu:osuc_occurrences:SCAU__2011000619; samplingProtocol: none specified; eventDate: 2005-08-09; **Record Level:** modified: 2013-07-17T11:03:58Z; language: en; collectionID: urn:lsid:biocol.org:col:34252; collectionCode: Insects; basisOfRecord: PreservedSpecimen; source: http://hol.osu.edu/spmInfo.html?id=SCAU%202011000619
- **Type status:** Other material. **Occurrence:** catalogNumber: SCAU 2011000618; recordedBy: Zhang Hong-Ying; individualCount: 1; sex: female; lifeStage: adult; **Taxon:** taxonID: urn:lsid:biosci.ohio-state.edu:osuc_names:275500; scientificName: Oxyscelio convergens; **Location:** country: China; stateProvince: Zhejiang; locality: Mt Qingliangfeng, Zhejiang Prov., China; locationRemarks: label transliteration: "Zhejiang, Qingliangfeng, 2005.08.09, Zhang Hongying"; [浙江清凉峰 2005.08.09 张红英]; decimalLatitude: 30.0703; decimalLongitude: 118.8944; georeferenceProtocol: Google Earth; georeferenceRemarks: GPS coords. adjusted to place within Zhejiang Prov.; **Identification:** identifiedBy: Norman F. Johnson; dateIdentified: 2012; **Event:** eventID: urn:lsid:biosci.ohio-state.edu:osuc_occurrences:SCAU__2011000618; samplingProtocol: none specified; eventDate: 2005-08-09; **Record Level:** modified: 2013-07-17T11:03:58Z; language: en; collectionID: urn:lsid:biocol.org:col:34252; collectionCode: Insects; basisOfRecord: PreservedSpecimen; source: http://hol.osu.edu/spmInfo.html?id=SCAU%202011000618
- **Type status:** Other material. **Occurrence:** catalogNumber: SCAU 2011000614; recordedBy: Zhang Hong-Ying; individualCount: 1; sex: male; lifeStage: adult; **Taxon:** taxonID: urn:lsid:biosci.ohio-state.edu:osuc_names:275500; scientificName: Oxyscelio convergens; **Location:** country: China; stateProvince: Zhejiang; locality: Mt Qingliangfeng, Zhejiang Prov., China; locationRemarks: label transliteration: "Zhejiang, Qingliangfeng, 2005.08.09, Zhang Hongying"; [浙江清凉峰 2005.08.09 张红英]; decimalLatitude: 30.0703; decimalLongitude: 118.8944; georeferenceProtocol: Google Earth; georeferenceRemarks: GPS coords. adjusted to place within Zhejiang Prov.; **Identification:** identifiedBy: Norman F. Johnson; dateIdentified: 2012; **Event:** eventID: urn:lsid:biosci.ohio-state.edu:osuc_occurrences:SCAU__2011000614; samplingProtocol: none specified; eventDate: 2005-08-09; **Record Level:** modified: 2013-07-17T11:03:58Z; language: en; collectionID: urn:lsid:biocol.org:col:34252; collectionCode: Insects; basisOfRecord: PreservedSpecimen; source: http://hol.osu.edu/spmInfo.html?id=SCAU%202011000614
- **Type status:** Other material. **Occurrence:** catalogNumber: SCAU 2011000613; recordedBy: Zhang Hong-Ying; individualCount: 1; sex: male; lifeStage: adult; **Taxon:** taxonID: urn:lsid:biosci.ohio-state.edu:osuc_names:275500; scientificName: Oxyscelio convergens; **Location:** country: China; stateProvince: Zhejiang; locality: Mt Qingliangfeng, Zhejiang Prov., China; locationRemarks: label transliteration: "Zhejiang, Qingliangfeng, 2005.08.09, Zhang Hongying"; [浙江清凉峰 2005.08.09 张红英]; decimalLatitude: 30.0703; decimalLongitude: 118.8944; georeferenceProtocol: Google Earth; georeferenceRemarks: GPS coords. adjusted to place within Zhejiang Prov.; **Identification:** identifiedBy: Norman F. Johnson; dateIdentified: 2012; **Event:** eventID: urn:lsid:biosci.ohio-state.edu:osuc_occurrences:SCAU__2011000613; samplingProtocol: none specified; eventDate: 2005-08-09; **Record Level:** modified: 2013-07-17T11:03:58Z; language: en; collectionID: urn:lsid:biocol.org:col:34252; collectionCode: Insects; basisOfRecord: PreservedSpecimen; source: http://hol.osu.edu/spmInfo.html?id=SCAU%202011000613
- **Type status:** Other material. **Occurrence:** catalogNumber: SCAU 2011000469; recordedBy: Shi Min; individualCount: 1; sex: male; lifeStage: adult; **Taxon:** taxonID: urn:lsid:biosci.ohio-state.edu:osuc_names:275500; scientificName: Oxyscelio convergens; **Location:** country: China; stateProvince: Zhejiang; locality: Mt Qingliangfeng, Zhejiang Prov., China; locationRemarks: label transliteration: "Zhejiang, Qingliangfeng, 2005.08.09, Shi Min"; [浙江清凉峰 2005.08.09 时敏]; decimalLatitude: 30.0703; decimalLongitude: 118.8944; georeferenceProtocol: Google Earth; georeferenceRemarks: GPS coords. adjusted to place within Zhejiang Prov.; **Identification:** identifiedBy: Norman F. Johnson; dateIdentified: 2012; **Event:** eventID: urn:lsid:biosci.ohio-state.edu:osuc_occurrences:SCAU__2011000469; samplingProtocol: none specified; eventDate: 2005-08-09; **Record Level:** modified: 2013-07-17T11:03:53Z; language: en; collectionID: urn:lsid:biocol.org:col:34252; collectionCode: Insects; basisOfRecord: PreservedSpecimen; source: http://hol.osu.edu/spmInfo.html?id=SCAU%202011000469
- **Type status:** Other material. **Occurrence:** catalogNumber: SCAU 2011000465; recordedBy: Shi Min; individualCount: 1; sex: female; lifeStage: adult; **Taxon:** taxonID: urn:lsid:biosci.ohio-state.edu:osuc_names:275500; scientificName: Oxyscelio convergens; **Location:** country: China; stateProvince: Zhejiang; locality: Mt Qingliangfeng, Zhejiang Prov., China; locationRemarks: label transliteration: "Zhejiang, Qingliangfeng, 2005.08.09, Shi Min"; [浙江清凉峰 2005.08.09 时敏]; decimalLatitude: 30.0703; decimalLongitude: 118.8944; georeferenceProtocol: Google Earth; georeferenceRemarks: GPS coords. adjusted to place within Zhejiang Prov.; **Identification:** identifiedBy: Norman F. Johnson; dateIdentified: 2012; **Event:** eventID: urn:lsid:biosci.ohio-state.edu:osuc_occurrences:SCAU__2011000465; samplingProtocol: none specified; eventDate: 2005-08-09; **Record Level:** modified: 2013-07-17T11:03:52Z; language: en; collectionID: urn:lsid:biocol.org:col:34252; collectionCode: Insects; basisOfRecord: PreservedSpecimen; source: http://hol.osu.edu/spmInfo.html?id=SCAU%202011000465
- **Type status:** Other material. **Occurrence:** catalogNumber: SCAU 2011000412; recordedBy: Shi Min; individualCount: 1; sex: male; lifeStage: adult; **Taxon:** taxonID: urn:lsid:biosci.ohio-state.edu:osuc_names:275500; scientificName: Oxyscelio convergens; **Location:** country: China; stateProvince: Hebei; county: Zhangjiakou; locality: Mt Dongling, eastern Zhangjiakou City, Hebei Prov., China; locationRemarks: label transliteration: "Hebei, Zhangjiakou, Donglingshan, 2005.08.11, Shi Min"; [河北张家口东灵山, 2005.08.11, 时敏]; decimalLatitude: 40.0333; decimalLongitude: 115.45; georeferenceProtocol: GPS; **Identification:** identifiedBy: Norman F. Johnson; dateIdentified: 2012; **Event:** eventID: urn:lsid:biosci.ohio-state.edu:osuc_occurrences:SCAU__2011000412; samplingProtocol: none specified; eventDate: 2005-08-11; **Record Level:** modified: 2013-07-17T11:03:50Z; language: en; collectionID: urn:lsid:biocol.org:col:34252; collectionCode: Insects; basisOfRecord: PreservedSpecimen; source: http://hol.osu.edu/spmInfo.html?id=SCAU%202011000412
- **Type status:** Other material. **Occurrence:** catalogNumber: SCAU 2011000681; recordedBy: Shi Min; individualCount: 1; sex: female; lifeStage: adult; **Taxon:** taxonID: urn:lsid:biosci.ohio-state.edu:osuc_names:275500; scientificName: Oxyscelio convergens; **Location:** country: China; stateProvince: Zhejiang; locality: Mt Qingliangfeng, Zhejiang Prov., China; locationRemarks: label transliteration: "Zhejiang, Qingliangfeng, 2005.08.11, Shi Min"; [浙江清凉峰 2005.08.11 时敏]; decimalLatitude: 30.0703; decimalLongitude: 118.8944; georeferenceProtocol: Google Earth; georeferenceRemarks: GPS coords. adjusted to place within Zhejiang Prov.; **Identification:** identifiedBy: Norman F. Johnson; dateIdentified: 2012; **Event:** eventID: urn:lsid:biosci.ohio-state.edu:osuc_occurrences:SCAU__2011000681; samplingProtocol: none specified; eventDate: 2005-08-11; **Record Level:** modified: 2013-07-17T11:04:03Z; language: en; collectionID: urn:lsid:biocol.org:col:34252; collectionCode: Insects; basisOfRecord: PreservedSpecimen; source: http://hol.osu.edu/spmInfo.html?id=SCAU%202011000681
- **Type status:** Other material. **Occurrence:** catalogNumber: SCAU 2011000682; recordedBy: Shi Min; individualCount: 1; sex: female; lifeStage: adult; **Taxon:** taxonID: urn:lsid:biosci.ohio-state.edu:osuc_names:275500; scientificName: Oxyscelio convergens; **Location:** country: China; stateProvince: Zhejiang; locality: Mt Qingliangfeng, Zhejiang Prov., China; locationRemarks: label transliteration: "Zhejiang, Qingliangfeng, 2005.08.11, Shi Min"; [浙江清凉峰 2005.08.11 时敏]; decimalLatitude: 30.0703; decimalLongitude: 118.8944; georeferenceProtocol: Google Earth; georeferenceRemarks: GPS coords. adjusted to place within Zhejiang Prov.; **Identification:** identifiedBy: Norman F. Johnson; dateIdentified: 2012; **Event:** eventID: urn:lsid:biosci.ohio-state.edu:osuc_occurrences:SCAU__2011000682; samplingProtocol: none specified; eventDate: 2005-08-11; **Record Level:** modified: 2013-07-17T11:04:03Z; language: en; collectionID: urn:lsid:biocol.org:col:34252; collectionCode: Insects; basisOfRecord: PreservedSpecimen; source: http://hol.osu.edu/spmInfo.html?id=SCAU%202011000682
- **Type status:** Other material. **Occurrence:** catalogNumber: SCAU 2011000680; recordedBy: Shi Min; individualCount: 1; sex: female; lifeStage: adult; **Taxon:** taxonID: urn:lsid:biosci.ohio-state.edu:osuc_names:275500; scientificName: Oxyscelio convergens; **Location:** country: China; stateProvince: Zhejiang; locality: Mt Qingliangfeng, Zhejiang Prov., China; locationRemarks: label transliteration: "Zhejiang, Qingliangfeng, 2005.08.11, Shi Min"; [浙江清凉峰 2005.08.11 时敏]; decimalLatitude: 30.0703; decimalLongitude: 118.8944; georeferenceProtocol: Google Earth; georeferenceRemarks: GPS coords. adjusted to place within Zhejiang Prov.; **Identification:** identifiedBy: Norman F. Johnson; dateIdentified: 2012; **Event:** eventID: urn:lsid:biosci.ohio-state.edu:osuc_occurrences:SCAU__2011000680; samplingProtocol: none specified; eventDate: 2005-08-11; **Record Level:** modified: 2013-07-17T11:04:03Z; language: en; collectionID: urn:lsid:biocol.org:col:34252; collectionCode: Insects; basisOfRecord: PreservedSpecimen; source: http://hol.osu.edu/spmInfo.html?id=SCAU%202011000680
- **Type status:** Other material. **Occurrence:** catalogNumber: SCAU 200705962; recordedBy: Weng Li-Qiong; individualCount: 1; sex: female; lifeStage: adult; **Taxon:** taxonID: urn:lsid:biosci.ohio-state.edu:osuc_names:275500; scientificName: Oxyscelio convergens; **Location:** country: China; stateProvince: Hainan; locality: Wuzhishan National Nature Reserve, Hainan Prov. China; locationRemarks: label transliteration: "Hainan, Wuzhishan, 2007.5.16-18, Weng Liqiong"; [海南五指山水满，2007.5.16-18，翁丽琼]; decimalLatitude: 18.85; decimalLongitude: 109.65; georeferenceProtocol: GPS; **Identification:** identifiedBy: Norman F. Johnson; dateIdentified: 2012; **Event:** eventID: urn:lsid:biosci.ohio-state.edu:osuc_occurrences:SCAU__200705962; samplingProtocol: none specified; eventDate: 2007-05-16/18; **Record Level:** modified: 2013-07-17T11:03:12Z; language: en; collectionID: urn:lsid:biocol.org:col:34252; collectionCode: Insects; basisOfRecord: PreservedSpecimen; source: http://hol.osu.edu/spmInfo.html?id=SCAU%20200705962
- **Type status:** Other material. **Occurrence:** catalogNumber: SCAU 200705956; recordedBy: Weng Li-Qiong; individualCount: 1; sex: female; lifeStage: adult; **Taxon:** taxonID: urn:lsid:biosci.ohio-state.edu:osuc_names:275500; scientificName: Oxyscelio convergens; **Location:** country: China; stateProvince: Hainan; locality: Wuzhishan National Nature Reserve, Hainan Prov. China; locationRemarks: label transliteration: "Hainan, Wuzhishan, 2007.5.16-18, Weng Liqiong"; [海南五指山水满，2007.5.16-18，翁丽琼]; decimalLatitude: 18.85; decimalLongitude: 109.65; georeferenceProtocol: GPS; **Identification:** identifiedBy: Norman F. Johnson; dateIdentified: 2012; **Event:** eventID: urn:lsid:biosci.ohio-state.edu:osuc_occurrences:SCAU__200705956; samplingProtocol: none specified; eventDate: 2007-05-16/18; **Record Level:** modified: 2013-07-17T11:03:12Z; language: en; collectionID: urn:lsid:biocol.org:col:34252; collectionCode: Insects; basisOfRecord: PreservedSpecimen; source: http://hol.osu.edu/spmInfo.html?id=SCAU%20200705956
- **Type status:** Other material. **Occurrence:** catalogNumber: SCAU 2011000656; recordedBy: Xiao Bin; individualCount: 1; sex: male; lifeStage: adult; **Taxon:** taxonID: urn:lsid:biosci.ohio-state.edu:osuc_names:275500; scientificName: Oxyscelio convergens; **Location:** country: China; stateProvince: Hainan; locality: Mt Diaoluo, Hainan Prov., China; locationRemarks: label transliteration: "Hainan, Diao luo shan,2007.5.29, Xiao Bin "; [海南吊罗山, 2007.5.29, 肖斌]; decimalLatitude: 18.65; decimalLongitude: 109.8833; georeferenceProtocol: GPS; **Identification:** identifiedBy: Norman F. Johnson; dateIdentified: 2012; **Event:** eventID: urn:lsid:biosci.ohio-state.edu:osuc_occurrences:SCAU__2011000656; samplingProtocol: none specified; eventDate: 2007-05-29; **Record Level:** modified: 2013-07-17T11:04:02Z; language: en; collectionID: urn:lsid:biocol.org:col:34252; collectionCode: Insects; basisOfRecord: PreservedSpecimen; source: http://hol.osu.edu/spmInfo.html?id=SCAU%202011000656
- **Type status:** Other material. **Occurrence:** catalogNumber: SCAU 200706323; recordedBy: Weng Li-Qiong; individualCount: 1; sex: male; lifeStage: adult; **Taxon:** taxonID: urn:lsid:biosci.ohio-state.edu:osuc_names:275500; scientificName: Oxyscelio convergens; **Location:** country: China; stateProvince: Hainan; locality: Jianfengling National Nature Reserve, Hainan Prov., China; locationRemarks: label transliteration: "Hainan, Jianfengling, 2007.6.5-7, Weng Liqiong"; [海南尖峰岭，2007.6.5-7，翁丽琼]; decimalLatitude: 18.6833; decimalLongitude: 108.8167; georeferenceProtocol: GPS; **Identification:** identifiedBy: Norman F. Johnson; dateIdentified: 2012; **Event:** eventID: urn:lsid:biosci.ohio-state.edu:osuc_occurrences:SCAU__200706323; samplingProtocol: none specified; eventDate: 2007-06-05/07; **Record Level:** modified: 2013-07-17T11:03:13Z; language: en; collectionID: urn:lsid:biocol.org:col:34252; collectionCode: Insects; basisOfRecord: PreservedSpecimen; source: http://hol.osu.edu/spmInfo.html?id=SCAU%20200706323
- **Type status:** Other material. **Occurrence:** catalogNumber: SCAU 200706326; recordedBy: Weng Li-Qiong; individualCount: 1; sex: male; lifeStage: adult; **Taxon:** taxonID: urn:lsid:biosci.ohio-state.edu:osuc_names:275500; scientificName: Oxyscelio convergens; **Location:** country: China; stateProvince: Hainan; locality: Jianfengling National Nature Reserve, Hainan Prov., China; locationRemarks: label transliteration: "Hainan, Jianfengling, 2007.6.5-7, Weng Liqiong"; [海南尖峰岭, 2007.6.5-7, 翁丽琼]; decimalLatitude: 18.6833; decimalLongitude: 108.8167; georeferenceProtocol: GPS; **Identification:** identifiedBy: Norman F. Johnson; dateIdentified: 2012; **Event:** eventID: urn:lsid:biosci.ohio-state.edu:osuc_occurrences:SCAU__200706326; samplingProtocol: none specified; eventDate: 2007-06-05/07; **Record Level:** modified: 2013-07-17T11:03:13Z; language: en; collectionID: urn:lsid:biocol.org:col:34252; collectionCode: Insects; basisOfRecord: PreservedSpecimen; source: http://hol.osu.edu/spmInfo.html?id=SCAU%20200706326
- **Type status:** Other material. **Occurrence:** catalogNumber: SCAU 200706329; recordedBy: Weng Li-Qiong; individualCount: 1; sex: female; lifeStage: adult; **Taxon:** taxonID: urn:lsid:biosci.ohio-state.edu:osuc_names:275500; scientificName: Oxyscelio convergens; **Location:** country: China; stateProvince: Hainan; locality: Jianfengling National Nature Reserve, Hainan Prov., China; locationRemarks: label transliteration: "Hainan, Jianfengling, 2007.6.5-7, Weng Liqiong"; [海南尖峰岭, 2007.6.5-7, 翁丽琼]; decimalLatitude: 18.6833; decimalLongitude: 108.8167; georeferenceProtocol: GPS; **Identification:** identifiedBy: Norman F. Johnson; dateIdentified: 2012; **Event:** eventID: urn:lsid:biosci.ohio-state.edu:osuc_occurrences:SCAU__200706329; samplingProtocol: none specified; eventDate: 2007-06-05/07; **Record Level:** modified: 2013-07-17T11:03:13Z; language: en; collectionID: urn:lsid:biocol.org:col:34252; collectionCode: Insects; basisOfRecord: PreservedSpecimen; source: http://hol.osu.edu/spmInfo.html?id=SCAU%20200706329
- **Type status:** Other material. **Occurrence:** catalogNumber: SCAU 200706342; recordedBy: Weng Li-Qiong; individualCount: 1; sex: female; lifeStage: adult; **Taxon:** taxonID: urn:lsid:biosci.ohio-state.edu:osuc_names:275500; scientificName: Oxyscelio convergens; **Location:** country: China; stateProvince: Hainan; locality: Jianfengling National Nature Reserve, Hainan Prov., China; locationRemarks: label transliteration: "Hainan, Jianfengling, 2007.6.5-7, Weng Liqiong"; [海南尖峰岭, 2007.6.5-7, 翁丽琼]; decimalLatitude: 18.6833; decimalLongitude: 108.8167; georeferenceProtocol: GPS; **Identification:** identifiedBy: Norman F. Johnson; dateIdentified: 2012; **Event:** eventID: urn:lsid:biosci.ohio-state.edu:osuc_occurrences:SCAU__200706342; samplingProtocol: none specified; eventDate: 2007-06-05/07; **Record Level:** modified: 2013-07-17T11:03:14Z; language: en; collectionID: urn:lsid:biocol.org:col:34252; collectionCode: Insects; basisOfRecord: PreservedSpecimen; source: http://hol.osu.edu/spmInfo.html?id=SCAU%20200706342
- **Type status:** Other material. **Occurrence:** catalogNumber: SCAU 200706347; recordedBy: Weng Li-Qiong; individualCount: 1; sex: male; lifeStage: adult; **Taxon:** taxonID: urn:lsid:biosci.ohio-state.edu:osuc_names:275500; scientificName: Oxyscelio convergens; **Location:** country: China; stateProvince: Hainan; locality: Jianfengling National Nature Reserve, Hainan Prov., China; locationRemarks: label transliteration: "Hainan, Jianfengling, 2007.6.5-7, Weng Liqiong"; [海南尖峰岭, 2007.6.5-7, 翁丽琼]; decimalLatitude: 18.6833; decimalLongitude: 108.8167; georeferenceProtocol: GPS; **Identification:** identifiedBy: Norman F. Johnson; dateIdentified: 2012; **Event:** eventID: urn:lsid:biosci.ohio-state.edu:osuc_occurrences:SCAU__200706347; samplingProtocol: none specified; eventDate: 2007-06-05/07; **Record Level:** modified: 2013-07-17T11:03:14Z; language: en; collectionID: urn:lsid:biocol.org:col:34252; collectionCode: Insects; basisOfRecord: PreservedSpecimen; source: http://hol.osu.edu/spmInfo.html?id=SCAU%20200706347
- **Type status:** Other material. **Occurrence:** catalogNumber: SCAU 200706348; recordedBy: Weng Li-Qiong; individualCount: 1; sex: male; lifeStage: adult; **Taxon:** taxonID: urn:lsid:biosci.ohio-state.edu:osuc_names:275500; scientificName: Oxyscelio convergens; **Location:** country: China; stateProvince: Hainan; locality: Jianfengling National Nature Reserve, Hainan Prov., China; locationRemarks: label transliteration: "Hainan, Jianfengling, 2007.6.5-7, Weng Liqiong"; [海南尖峰岭, 2007.6.5-7, 翁丽琼]; decimalLatitude: 18.6833; decimalLongitude: 108.8167; georeferenceProtocol: GPS; **Identification:** identifiedBy: Norman F. Johnson; dateIdentified: 2012; **Event:** eventID: urn:lsid:biosci.ohio-state.edu:osuc_occurrences:SCAU__200706348; samplingProtocol: none specified; eventDate: 2007-06-05/07; **Record Level:** modified: 2013-07-17T11:03:14Z; language: en; collectionID: urn:lsid:biocol.org:col:34252; collectionCode: Insects; basisOfRecord: PreservedSpecimen; source: http://hol.osu.edu/spmInfo.html?id=SCAU%20200706348
- **Type status:** Other material. **Occurrence:** catalogNumber: SCAU 200706330; recordedBy: Weng Li-Qiong; individualCount: 1; sex: male; lifeStage: adult; **Taxon:** taxonID: urn:lsid:biosci.ohio-state.edu:osuc_names:275500; scientificName: Oxyscelio convergens; **Location:** country: China; stateProvince: Hainan; locality: Jianfengling National Nature Reserve, Hainan Prov., China; locationRemarks: label transliteration: "Hainan, Jianfengling, 2007.6.5-7, Weng Liqiong"; [海南尖峰岭, 2007.6.5-7, 翁丽琼]; decimalLatitude: 18.6833; decimalLongitude: 108.8167; georeferenceProtocol: GPS; **Identification:** identifiedBy: Norman F. Johnson; dateIdentified: 2012; **Event:** eventID: urn:lsid:biosci.ohio-state.edu:osuc_occurrences:SCAU__200706330; samplingProtocol: none specified; eventDate: 2007-06-05/07; **Record Level:** modified: 2013-07-17T11:03:13Z; language: en; collectionID: urn:lsid:biocol.org:col:34252; collectionCode: Insects; basisOfRecord: PreservedSpecimen; source: http://hol.osu.edu/spmInfo.html?id=SCAU%20200706330
- **Type status:** Other material. **Occurrence:** catalogNumber: SCAU 201010087; recordedBy: Yao Jie-Min; individualCount: 1; sex: male; lifeStage: adult; **Taxon:** taxonID: urn:lsid:biosci.ohio-state.edu:osuc_names:275500; scientificName: Oxyscelio convergens; **Location:** country: China; stateProvince: Hainan; locality: Jianfengling National Nature Reserve, Hainan Prov., China; locationRemarks: label transliteration: "Hainan, Jianfengling, 2007.10.23-25, Yao Jiemin"; [海南尖峰岭，2007.10.23-25，姚婕敏]; decimalLatitude: 18.6833; decimalLongitude: 108.8167; georeferenceProtocol: GPS; **Identification:** identifiedBy: Norman F. Johnson; dateIdentified: 2012; **Event:** eventID: urn:lsid:biosci.ohio-state.edu:osuc_occurrences:SCAU__201010087; samplingProtocol: none specified; eventDate: 2007-10-23/25; **Record Level:** modified: 2013-07-17T11:03:27Z; language: en; collectionID: urn:lsid:biocol.org:col:34252; collectionCode: Insects; basisOfRecord: PreservedSpecimen; source: http://hol.osu.edu/spmInfo.html?id=SCAU%20201010087
- **Type status:** Other material. **Occurrence:** catalogNumber: SCAU 201010092; recordedBy: Yao Jie-Min; individualCount: 1; sex: male; lifeStage: adult; **Taxon:** taxonID: urn:lsid:biosci.ohio-state.edu:osuc_names:275500; scientificName: Oxyscelio convergens; **Location:** country: China; stateProvince: Hainan; locality: Jianfengling National Nature Reserve, Hainan Prov., China; locationRemarks: label transliteration: "Hainan, Jianfengling, 2007.10.23-25, Yao Jiemin"; [海南尖峰岭，2007.10.23-25，姚婕敏]; decimalLatitude: 18.6833; decimalLongitude: 108.8167; georeferenceProtocol: GPS; **Identification:** identifiedBy: Norman F. Johnson; dateIdentified: 2012; **Event:** eventID: urn:lsid:biosci.ohio-state.edu:osuc_occurrences:SCAU__201010092; samplingProtocol: none specified; eventDate: 2007-10-23/25; **Record Level:** modified: 2013-07-17T11:03:27Z; language: en; collectionID: urn:lsid:biocol.org:col:34252; collectionCode: Insects; basisOfRecord: PreservedSpecimen; source: http://hol.osu.edu/spmInfo.html?id=SCAU%20201010092
- **Type status:** Other material. **Occurrence:** catalogNumber: SCAU 2011000266; recordedBy: Tan Jiang-Li; individualCount: 1; sex: male; lifeStage: adult; **Taxon:** taxonID: urn:lsid:biosci.ohio-state.edu:osuc_names:275500; scientificName: Oxyscelio convergens; **Location:** country: China; stateProvince: Hainan; locality: Jianfengling National Nature Reserve, Hainan Prov., China; locationRemarks: label transliteration: "Hainan, Jianfengling, 2008.11.18, Tan Jiangli"; [海南尖峰岭, 2008.11.18, 谭江丽]; decimalLatitude: 18.6833; decimalLongitude: 108.8167; georeferenceProtocol: GPS; **Identification:** identifiedBy: Norman F. Johnson; dateIdentified: 2012; **Event:** eventID: urn:lsid:biosci.ohio-state.edu:osuc_occurrences:SCAU__2011000266; samplingProtocol: none specified; eventDate: 2008-11-18; **Record Level:** modified: 2013-07-17T11:03:47Z; language: en; collectionID: urn:lsid:biocol.org:col:34252; collectionCode: Insects; basisOfRecord: PreservedSpecimen; source: http://hol.osu.edu/spmInfo.html?id=SCAU%202011000266
- **Type status:** Other material. **Occurrence:** catalogNumber: SCAU 2011000267; recordedBy: Tan Jiang-Li; individualCount: 1; sex: male; lifeStage: adult; **Taxon:** taxonID: urn:lsid:biosci.ohio-state.edu:osuc_names:275500; scientificName: Oxyscelio convergens; **Location:** country: China; stateProvince: Hainan; locality: Jianfengling National Nature Reserve, Hainan Prov., China; locationRemarks: label transliteration: "Hainan, Jianfengling, 2008.11.25, Tan Jiangli"; [海南尖峰岭, 2008.11.25, 谭江丽]; decimalLatitude: 18.6833; decimalLongitude: 108.8167; georeferenceProtocol: GPS; **Identification:** identifiedBy: Norman F. Johnson; dateIdentified: 2012; **Event:** eventID: urn:lsid:biosci.ohio-state.edu:osuc_occurrences:SCAU__2011000267; samplingProtocol: none specified; eventDate: 2008-11-25; **Record Level:** modified: 2013-07-17T11:03:48Z; language: en; collectionID: urn:lsid:biocol.org:col:34252; collectionCode: Insects; basisOfRecord: PreservedSpecimen; source: http://hol.osu.edu/spmInfo.html?id=SCAU%202011000267
- **Type status:** Other material. **Occurrence:** catalogNumber: SCAU 2011000327; recordedBy: Tang Pu; individualCount: 1; sex: male; lifeStage: adult; **Taxon:** taxonID: urn:lsid:biosci.ohio-state.edu:osuc_names:275500; scientificName: Oxyscelio convergens; **Location:** country: Taiwan; stateProvince: Taiwan; county: Pingtung; locality: Kenting National Forest Recreation Area, 21°57'N 120°48'E, Pingtung Co., Taiwan Prov., Taiwan; locationRemarks: label transliteration: "Taiwan, Pingdong, Kenting National Forest Park, Tang Po"; [台湾屏东垦丁国家森林公园 21°57'N 120°48'E, 2011.05.31, sweeping, 唐璞]; decimalLatitude: 21.95; decimalLongitude: 120.8; georeferenceProtocol: label; **Identification:** identifiedBy: Norman F. Johnson; dateIdentified: 2012; **Event:** eventID: urn:lsid:biosci.ohio-state.edu:osuc_occurrences:SCAU__2011000327; samplingProtocol: sweeping; eventDate: 2011-05-31; **Record Level:** modified: 2013-07-17T11:03:48Z; language: en; collectionID: urn:lsid:biocol.org:col:34252; collectionCode: Insects; basisOfRecord: PreservedSpecimen; source: http://hol.osu.edu/spmInfo.html?id=SCAU%202011000327
- **Type status:** Other material. **Occurrence:** catalogNumber: SCAU 2011000088; recordedBy: Jin Cheng-Yuan; individualCount: 1; sex: male; lifeStage: adult; **Taxon:** taxonID: urn:lsid:biosci.ohio-state.edu:osuc_names:275500; scientificName: Oxyscelio convergens; **Location:** country: China; stateProvince: Zhejiang; locality: Tianmushan National Nature Reserve, 30°20.56'N 119°26.03'E, 1200m, Mt. Xianrending, Zhejiang Prov., China; locationRemarks: label transliteration: "Zhejiang, Tianmushan, Xianrending, 2011.07.25-29, Jin Chengyuan"; [浙江天目山仙人顶 1200 m, 30°20.56'N 119°26.03'E, 2011.07.25-29, sweeping, 金程远]; decimalLatitude: 30.3427; decimalLongitude: 119.4338; georeferenceProtocol: label; **Identification:** identifiedBy: Norman F. Johnson; dateIdentified: 2012; **Event:** eventID: urn:lsid:biosci.ohio-state.edu:osuc_occurrences:SCAU__2011000088; samplingProtocol: sweeping; eventDate: 2011-07-25/29; **Record Level:** modified: 2013-07-17T11:03:34Z; language: en; collectionID: urn:lsid:biocol.org:col:34252; collectionCode: Insects; basisOfRecord: PreservedSpecimen; source: http://hol.osu.edu/spmInfo.html?id=SCAU%202011000088

#### Distribution

This species was previously known only from Taiwan. It is here recorded from Hebei in the north, Zhejiang in eastern China, and Hainan in the south. Link to dynamic distribution map: http://hol.osu.edu/map-large.html?id=275500

### Oxyscelio cordis

Burks, 2013

http://lsid.tdwg.org/urn:lsid:biosci.ohio-state.edu:osuc_concepts:275553

urn:lsid:zoobank.org:act:ED24C4B5-414C-4D6A-8713-14766B7DDB19

http://species-id.net/wiki/Oxyscelio_cordis

Oxyscelio cordis Burks et al. 2013: 14, 21, 87. Original description, keyed, placed in *crateris* species group.

#### Materials

- **Type status:** Other material. **Occurrence:** catalogNumber: SCAU 200906481; recordedBy: Wang Man-Man; individualCount: 1; sex: male; lifeStage: adult; **Taxon:** taxonID: urn:lsid:biosci.ohio-state.edu:osuc_names:275553; scientificName: Oxyscelio cordis; **Location:** country: China; stateProvince: Yunnan; county: Dehong Dai and Jingpo; locality: Tongbiguan Pass; locationRemarks: label transliteration: "Yunnan, Yingjiang, Tongbiguan, 2009.05.16, Wang Manman "; [云南盈江铜壁关, 2009.05.16, 王漫漫]; decimalLatitude: 24.25; decimalLongitude: 97.8167; georeferenceProtocol: GPS; **Identification:** identifiedBy: Norman F. Johnson; dateIdentified: 2012; **Event:** eventID: urn:lsid:biosci.ohio-state.edu:osuc_occurrences:SCAU__200906481; samplingProtocol: none specified; eventDate: 2009-05-16; **Record Level:** modified: 2013-07-17T11:03:20Z; language: en; collectionID: urn:lsid:biocol.org:col:34252; collectionCode: Insects; basisOfRecord: PreservedSpecimen; source: http://hol.osu.edu/spmInfo.html?id=SCAU%20200906481
- **Type status:** Other material. **Occurrence:** catalogNumber: SCAU 200906469; recordedBy: Wang Man-Man; individualCount: 1; sex: male; lifeStage: adult; **Taxon:** taxonID: urn:lsid:biosci.ohio-state.edu:osuc_names:275553; scientificName: Oxyscelio cordis; **Location:** country: China; stateProvince: Yunnan; county: Dehong Dai and Jingpo; locality: Tongbiguan Pass; locationRemarks: label transliteration: "Yunnan, Yingjiang, Tongbiguan (pass), 2009.05.16, Wang Manman "; [云南盈江铜壁关, 2009.05.16, 王漫漫]; decimalLatitude: 24.25; decimalLongitude: 97.8167; georeferenceProtocol: GPS; **Identification:** identifiedBy: Norman F. Johnson; dateIdentified: 2012; **Event:** eventID: urn:lsid:biosci.ohio-state.edu:osuc_occurrences:SCAU__200906469; samplingProtocol: none specified; eventDate: 2009-05-16; **Record Level:** modified: 2013-07-17T11:03:19Z; language: en; collectionID: urn:lsid:biocol.org:col:34252; collectionCode: Insects; basisOfRecord: PreservedSpecimen; source: http://hol.osu.edu/spmInfo.html?id=SCAU%20200906469
- **Type status:** Other material. **Occurrence:** catalogNumber: SCAU 200906462; recordedBy: Wang Man-Man; individualCount: 1; sex: male; lifeStage: adult; **Taxon:** taxonID: urn:lsid:biosci.ohio-state.edu:osuc_names:275553; scientificName: Oxyscelio cordis; **Location:** country: China; stateProvince: Yunnan; county: Dehong Dai and Jingpo; locality: Tongbiguan Pass; locationRemarks: label transliteration: "Yunnan, Yingjiang, Tongbiguan, 2009.05.16, Wang Manman "; [云南盈江铜壁关, 2009.05.16, 王漫漫]; decimalLatitude: 24.25; decimalLongitude: 97.8167; georeferenceProtocol: GPS; **Identification:** identifiedBy: Norman F. Johnson; dateIdentified: 2012; **Event:** eventID: urn:lsid:biosci.ohio-state.edu:osuc_occurrences:SCAU__200906462; samplingProtocol: none specified; eventDate: 2009-05-16; **Record Level:** modified: 2013-07-17T11:03:19Z; language: en; collectionID: urn:lsid:biocol.org:col:34252; collectionCode: Insects; basisOfRecord: PreservedSpecimen; source: http://hol.osu.edu/spmInfo.html?id=SCAU%20200906462
- **Type status:** Other material. **Occurrence:** catalogNumber: SCAU 200901544; recordedBy: Wang Man-Man; individualCount: 1; sex: female; lifeStage: adult; **Taxon:** taxonID: urn:lsid:biosci.ohio-state.edu:osuc_names:275553; scientificName: Oxyscelio cordis; **Location:** country: China; stateProvince: Yunnan; county: Dehong Dai and Jingpo; locality: Tongbiguan Pass; locationRemarks: label transliteration: "Yunnan, Yingjiang, Tongbiguan, 2009.05.17, Wang Manman "; [云南盈江铜壁关, 2009.05.17, 王漫漫]; decimalLatitude: 24.25; decimalLongitude: 97.8167; georeferenceProtocol: GPS; **Identification:** identifiedBy: Norman F. Johnson; dateIdentified: 2012; **Event:** eventID: urn:lsid:biosci.ohio-state.edu:osuc_occurrences:SCAU__200901544; samplingProtocol: none specified; eventDate: 2009-05-17; **Record Level:** modified: 2013-07-17T11:03:15Z; language: en; collectionID: urn:lsid:biocol.org:col:34252; collectionCode: Insects; basisOfRecord: PreservedSpecimen; source: http://hol.osu.edu/spmInfo.html?id=SCAU%20200901544

#### Distribution

*Oxyscelio cordis* was originally described from specimens collected in northern Thailand. It is here recorded from Yunnan, nearthe border with Myanmar. Link to dynamic distribution map: http://hol.osu.edu/map-large.html?id=275553

### Oxyscelio crebritas

Burks, 2013

http://lsid.tdwg.org/urn:lsid:biosci.ohio-state.edu:osuc_concepts:275371

urn:lsid:zoobank.org:act:82C94912-5D20-4C31-8447-1E6F470BFA53

http://species-id.net/wiki/Oxyscelio_crebritas

Oxyscelio crebritas Burks et al. 2013: 15, 22, 93. Original description, keyed, placed in crebritas species group.

#### Materials

- **Type status:** Other material. **Occurrence:** catalogNumber: SCAU 200705764; recordedBy: Liu Jing-Xian; individualCount: 1; sex: female; lifeStage: adult; **Taxon:** taxonID: urn:lsid:biosci.ohio-state.edu:osuc_names:275371; scientificName: Oxyscelio crebritas; **Location:** country: China; stateProvince: Hainan; locality: Wuzhishan National Nature Reserve; locationRemarks: label transliteration: "Hainan, Wuzhishan, Shuiman, 2007.5.16-18, Liu Jingxian"; [海南五指山水满，2007.5.16-18，刘经贤]; decimalLatitude: 18.85; decimalLongitude: 109.65; georeferenceProtocol: GPS; **Identification:** identifiedBy: Norman F. Johnson; dateIdentified: 2012; **Event:** eventID: urn:lsid:biosci.ohio-state.edu:osuc_occurrences:SCAU__200705764; samplingProtocol: none specified; eventDate: 2007-05-16/18; habitat: waterfall; **Record Level:** modified: 2013-07-17T11:03:11Z; language: en; collectionID: urn:lsid:biocol.org:col:34252; collectionCode: Insects; basisOfRecord: PreservedSpecimen; source: http://hol.osu.edu/spmInfo.html?id=SCAU%20200705764
- **Type status:** Other material. **Occurrence:** catalogNumber: SCAU 200705603; recordedBy: Zeng Jie; individualCount: 1; sex: female; lifeStage: adult; **Taxon:** taxonID: urn:lsid:biosci.ohio-state.edu:osuc_names:275371; scientificName: Oxyscelio crebritas; **Location:** country: China; stateProvince: Hainan; locality: Wuzhishan National Nature Reserve; locationRemarks: label transliteration: "Hainan, Wuzhishan, Shuiman, 2007.5.16-18, Zeng Jie"; [海南五指山水满，2007.5.16-18，曾洁]; decimalLatitude: 18.85; decimalLongitude: 109.65; georeferenceProtocol: GPS; **Identification:** identifiedBy: Norman F. Johnson; dateIdentified: 2012; **Event:** eventID: urn:lsid:biosci.ohio-state.edu:osuc_occurrences:SCAU__200705603; samplingProtocol: none specified; eventDate: 2007-05-16/18; habitat: waterfall; **Record Level:** modified: 2013-07-17T11:03:10Z; language: en; collectionID: urn:lsid:biocol.org:col:34252; collectionCode: Insects; basisOfRecord: PreservedSpecimen; source: http://hol.osu.edu/spmInfo.html?id=SCAU%20200705603
- **Type status:** Other material. **Occurrence:** catalogNumber: SCAU 2011000431; recordedBy: Weng Li-Qiong; individualCount: 1; sex: female; lifeStage: adult; **Taxon:** taxonID: urn:lsid:biosci.ohio-state.edu:osuc_names:275371; scientificName: Oxyscelio crebritas; **Location:** country: China; stateProvince: Hainan; locality: Jianfengling National Nature Reserve; locationRemarks: label transliteration: "Hainan, Jianfengling, 2007.6.5-7, Weng Liqiong"; [海南尖峰岭, 2007.6.5-7, 翁丽琼]; decimalLatitude: 18.6833; decimalLongitude: 108.8167; georeferenceProtocol: GPS; **Identification:** identifiedBy: Norman F. Johnson; dateIdentified: 2012; **Event:** eventID: urn:lsid:biosci.ohio-state.edu:osuc_occurrences:SCAU__2011000431; samplingProtocol: none specified; eventDate: 2007-06-05/07; **Record Level:** modified: 2013-07-17T11:03:51Z; language: en; collectionID: urn:lsid:biocol.org:col:34252; collectionCode: Insects; basisOfRecord: PreservedSpecimen; source: http://hol.osu.edu/spmInfo.html?id=SCAU%202011000431
- **Type status:** Other material. **Occurrence:** catalogNumber: SCAU 2011000025; recordedBy: Tang Pu; individualCount: 1; sex: female; lifeStage: adult; **Taxon:** taxonID: urn:lsid:biosci.ohio-state.edu:osuc_names:275371; scientificName: Oxyscelio crebritas; **Location:** country: Taiwan; stateProvince: Taiwan; county: Taitung; locality: Shouqia; locationRemarks: characters for the town should be 壽峠; label transliteration: "Taiwan, Taidon sheng, Shouka, Tang Po"; [台湾台东县寿卡, 22°14'N 120°50'E, sweeping, 唐璞]; verbatimCoordinates: 22°14'N 120°50'E; decimalLatitude: 22.2333; decimalLongitude: 120.8333; georeferenceProtocol: label; **Identification:** identifiedBy: Norman F. Johnson; dateIdentified: 2012; **Event:** eventID: urn:lsid:biosci.ohio-state.edu:osuc_occurrences:SCAU__2011000025; samplingProtocol: sweeping; **Record Level:** modified: 2013-07-17T11:03:27Z; language: en; collectionID: urn:lsid:biocol.org:col:34252; collectionCode: Insects; basisOfRecord: PreservedSpecimen; source: http://hol.osu.edu/spmInfo.html?id=SCAU%202011000025

#### Distribution

This species is widespread through southeast Asia, extending to Nepal in the northwest and Sulawesi and Taiwan in the east. The new data provide an additional record for Taiwan and documents the presence of the species in Hainan. Link to dynamic distribution map: http://hol.osu.edu/map-large.html?id=275371

### Oxyscelio cuculli

Burks, 2013

http://lsid.tdwg.org/urn:lsid:biosci.ohio-state.edu:osuc_concepts:275485

urn:lsid:zoobank.org:act:D09D8C28-2F8A-4E65-A1B6-D442FB123BF9

http://species-id.net/wiki/Oxyscelio_cuculli

Oxyscelio cuculli Burks et al. 2013: 15, 22, 100. Original description, keyed, placed in *cuculli* species group.

#### Materials

- **Type status:** Other material. **Occurrence:** catalogNumber: SCAU 2011000624; recordedBy: Zhang Hong-Ying; individualCount: 1; sex: female; lifeStage: adult; **Taxon:** taxonID: urn:lsid:biosci.ohio-state.edu:osuc_names:275485; scientificName: Oxyscelio cuculli; **Location:** country: China; stateProvince: Zhejiang; locality: Mt Qingliangfeng; locationRemarks: label transliteration: "Zhejiang, Qingliangfeng, 2005.08.09, Zhang Hongying"; [浙江清凉峰 2005.08.09 张红英]; decimalLatitude: 30.0703; decimalLongitude: 118.8944; georeferenceProtocol: Google Earth; georeferenceRemarks: GPS coords. adjusted to place within Zhejiang Prov.; **Identification:** identifiedBy: Norman F. Johnson; dateIdentified: 2012; **Event:** eventID: urn:lsid:biosci.ohio-state.edu:osuc_occurrences:SCAU__2011000624; samplingProtocol: none specified; eventDate: 2005-08-09; **Record Level:** modified: 2013-07-17T11:03:59Z; language: en; collectionID: urn:lsid:biocol.org:col:34252; collectionCode: Insects; basisOfRecord: PreservedSpecimen; source: http://hol.osu.edu/spmInfo.html?id=SCAU%202011000624
- **Type status:** Other material. **Occurrence:** catalogNumber: SCAU 2011000611; recordedBy: Zhang Hong-Ying; individualCount: 1; sex: male; lifeStage: adult; **Taxon:** taxonID: urn:lsid:biosci.ohio-state.edu:osuc_names:275485; scientificName: Oxyscelio cuculli; **Location:** country: China; stateProvince: Zhejiang; locality: Mt Qingliangfeng; locationRemarks: label transliteration: "Zhejiang, Qingliangfeng, 2005.08.09, Zhang Hongying"; [浙江清凉峰 2005.08.09 张红英]; decimalLatitude: 30.0703; decimalLongitude: 118.8944; georeferenceProtocol: Google Earth; georeferenceRemarks: GPS coords. adjusted to place within Zhejiang Prov.; **Identification:** identifiedBy: Norman F. Johnson; dateIdentified: 2012; **Event:** eventID: urn:lsid:biosci.ohio-state.edu:osuc_occurrences:SCAU__2011000611; samplingProtocol: none specified; eventDate: 2005-08-09; **Record Level:** modified: 2013-07-17T11:03:57Z; language: en; collectionID: urn:lsid:biocol.org:col:34252; collectionCode: Insects; basisOfRecord: PreservedSpecimen; source: http://hol.osu.edu/spmInfo.html?id=SCAU%202011000611
- **Type status:** Other material. **Occurrence:** catalogNumber: SCAU 2011000406; recordedBy: Shi Min; individualCount: 1; sex: female; lifeStage: adult; **Taxon:** taxonID: urn:lsid:biosci.ohio-state.edu:osuc_names:275485; scientificName: Oxyscelio cuculli; **Location:** country: China; stateProvince: Hebei; county: Zhangjiakou; locality: Mt Dongling; locationRemarks: label transliteration: "Hebei, Zhang jia kou, Donglingshan, 2005.08.11, Shi Min"; [河北张家口东灵山, 2005.08.11, 时敏]; decimalLatitude: 40.0333; decimalLongitude: 115.45; georeferenceProtocol: GPS; **Identification:** identifiedBy: Norman F. Johnson; dateIdentified: 2012; **Event:** eventID: urn:lsid:biosci.ohio-state.edu:osuc_occurrences:SCAU__2011000406; samplingProtocol: none specified; eventDate: 2005-08-11; **Record Level:** modified: 2013-07-17T11:03:50Z; language: en; collectionID: urn:lsid:biocol.org:col:34252; collectionCode: Insects; basisOfRecord: PreservedSpecimen; source: http://hol.osu.edu/spmInfo.html?id=SCAU%202011000406
- **Type status:** Other material. **Occurrence:** catalogNumber: SCAU 2011000402; recordedBy: Shi Min; individualCount: 1; sex: female; lifeStage: adult; **Taxon:** taxonID: urn:lsid:biosci.ohio-state.edu:osuc_names:275485; scientificName: Oxyscelio cuculli; **Location:** country: China; stateProvince: Hebei; county: Zhangjiakou; locality: Mt Dongling; locationRemarks: label transliteration: "Hebei, Zhang jia kou, Donglingshan, 2005.08.11, Shi Min"; [河北张家口东灵山, 2005.08.11, 时敏]; decimalLatitude: 40.0333; decimalLongitude: 115.45; georeferenceProtocol: GPS; **Identification:** identifiedBy: Norman F. Johnson; dateIdentified: 2012; **Event:** eventID: urn:lsid:biosci.ohio-state.edu:osuc_occurrences:SCAU__2011000402; samplingProtocol: none specified; eventDate: 2005-08-11; **Record Level:** modified: 2013-07-17T11:03:49Z; language: en; collectionID: urn:lsid:biocol.org:col:34252; collectionCode: Insects; basisOfRecord: PreservedSpecimen; source: http://hol.osu.edu/spmInfo.html?id=SCAU%202011000402
- **Type status:** Other material. **Occurrence:** catalogNumber: SCAU 2011000401; recordedBy: Shi Min; individualCount: 1; sex: male; lifeStage: adult; **Taxon:** taxonID: urn:lsid:biosci.ohio-state.edu:osuc_names:275485; scientificName: Oxyscelio cuculli; **Location:** country: China; stateProvince: Hebei; county: Zhangjiakou; locality: Mt Dongling; locationRemarks: label transliteration: "Hebei, Zhang jia kou, Donglingshan, 2005.08.11, Shi Min"; [河北张家口东灵山, 2005.08.11, 时敏]; decimalLatitude: 40.0333; decimalLongitude: 115.45; georeferenceProtocol: GPS; **Identification:** identifiedBy: Norman F. Johnson; dateIdentified: 2012; **Event:** eventID: urn:lsid:biosci.ohio-state.edu:osuc_occurrences:SCAU__2011000401; samplingProtocol: none specified; eventDate: 2005-08-11; **Record Level:** modified: 2013-07-17T11:03:49Z; language: en; collectionID: urn:lsid:biocol.org:col:34252; collectionCode: Insects; basisOfRecord: PreservedSpecimen; source: http://hol.osu.edu/spmInfo.html?id=SCAU%202011000401
- **Type status:** Other material. **Occurrence:** catalogNumber: SCAU 200906089; recordedBy: Liu Jing-Xian, et al.; individualCount: 1; sex: female; lifeStage: adult; **Taxon:** taxonID: urn:lsid:biosci.ohio-state.edu:osuc_names:275485; scientificName: Oxyscelio cuculli; **Location:** country: China; stateProvince: Hainan; locality: Bawangling National Nature Reserve; locationRemarks: label transliteration: "Hainan, Bawangling, 2006.07.07-11, Liu Jingxian et al."; [海南霸王岭, 2006.07.07-11, 刘经贤等]; decimalLatitude: 19.1167; decimalLongitude: 109.05; georeferenceProtocol: GPS; **Identification:** identifiedBy: Norman F. Johnson; dateIdentified: 2012; **Event:** eventID: urn:lsid:biosci.ohio-state.edu:osuc_occurrences:SCAU__200906089; samplingProtocol: none specified; eventDate: 2006-07-07/11; **Record Level:** modified: 2013-07-17T11:03:17Z; language: en; collectionID: urn:lsid:biocol.org:col:34252; collectionCode: Insects; basisOfRecord: PreservedSpecimen; source: http://hol.osu.edu/spmInfo.html?id=SCAU%20200906089
- **Type status:** Other material. **Occurrence:** catalogNumber: SCAU 2011000098; recordedBy: Jin Cheng-Yuan; individualCount: 1; sex: female; lifeStage: adult; **Taxon:** taxonID: urn:lsid:biosci.ohio-state.edu:osuc_names:275485; scientificName: Oxyscelio cuculli; **Location:** country: China; stateProvince: Zhejiang; locality: Tianmushan National Nature Reserve, Mt. Xianrending; verbatimElevation: 1200 m; locationRemarks: label transliteration: "Zhejiang, Tianmushan, Xianrending, 2011.07.25-29, Jin Chengyuan"; [浙江天目山仙人顶 1200 m, 30°20.56'N 119°26.03'E, 2011.07.25-29,YPT, 金程远]; verbatimCoordinates: 30°20.56'N 119°26.03'E; decimalLatitude: 30.3427; decimalLongitude: 119.4338; georeferenceProtocol: label; **Identification:** identifiedBy: Norman F. Johnson; dateIdentified: 2012; **Event:** eventID: urn:lsid:biosci.ohio-state.edu:osuc_occurrences:SCAU__2011000098; samplingProtocol: yellow pan trap; eventDate: 2011-07-25/29; **Record Level:** modified: 2013-07-17T11:03:38Z; language: en; collectionID: urn:lsid:biocol.org:col:34252; collectionCode: Insects; basisOfRecord: PreservedSpecimen; source: http://hol.osu.edu/spmInfo.html?id=SCAU%202011000098
- **Type status:** Other material. **Occurrence:** catalogNumber: SCAU 2011000096; recordedBy: Jin Cheng-Yuan; individualCount: 1; sex: female; lifeStage: adult; **Taxon:** taxonID: urn:lsid:biosci.ohio-state.edu:osuc_names:275485; scientificName: Oxyscelio cuculli; **Location:** country: China; stateProvince: Zhejiang; locality: Tianmushan National Nature Reserve, Mt. Xianrending; verbatimElevation: 1200 m; locationRemarks: label transliteration: "Zhejiang, Tianmushan, Xianrending, 2011.07.25-29, Jin Chengyuan"; [浙江天目山仙人顶 1200 m, 30°20.56'N 119°26.03'E, 2011.07.25-29, YPT, 金程远]; verbatimCoordinates: 30°20.56'N 119°26.03'E; decimalLatitude: 30.3427; decimalLongitude: 119.4338; georeferenceProtocol: label; **Identification:** identifiedBy: Norman F. Johnson; dateIdentified: 2012; **Event:** eventID: urn:lsid:biosci.ohio-state.edu:osuc_occurrences:SCAU__2011000096; samplingProtocol: yellow pan trap; eventDate: 2011-07-25/29; **Record Level:** modified: 2013-07-17T11:03:37Z; language: en; collectionID: urn:lsid:biocol.org:col:34252; collectionCode: Insects; basisOfRecord: PreservedSpecimen; source: http://hol.osu.edu/spmInfo.html?id=SCAU%202011000096
- **Type status:** Other material. **Occurrence:** catalogNumber: SCAU 2011000095; recordedBy: Jin Cheng-Yuan; individualCount: 1; sex: female; lifeStage: adult; **Taxon:** taxonID: urn:lsid:biosci.ohio-state.edu:osuc_names:275485; scientificName: Oxyscelio cuculli; **Location:** country: China; stateProvince: Zhejiang; locality: Tianmushan National Nature Reserve, Mt. Xianrending; verbatimElevation: 1200 m; locationRemarks: label transliteration: "Zhejiang, Tianmushan, Xianrending, 2011.07.25-29, Jin Chengyuan"; [浙江天目山仙人顶 1200 m, 30°20.56'N 119°26.03'E, 2011.07.25-29, YPT, 金程远]; verbatimCoordinates: 30°20.56'N 119°26.03'E; decimalLatitude: 30.3427; decimalLongitude: 119.4338; georeferenceProtocol: label; **Identification:** identifiedBy: Norman F. Johnson; dateIdentified: 2012; **Event:** eventID: urn:lsid:biosci.ohio-state.edu:osuc_occurrences:SCAU__2011000095; samplingProtocol: yellow pan trap; eventDate: 2011-07-25/29; **Record Level:** modified: 2013-07-17T11:03:37Z; language: en; collectionID: urn:lsid:biocol.org:col:34252; collectionCode: Insects; basisOfRecord: PreservedSpecimen; source: http://hol.osu.edu/spmInfo.html?id=SCAU%202011000095

#### Distribution

Like *Oxyscelio crebritas*, this species is widespread through southeast Asia. The new records document it from Hainan, Zhejiang, and as far north as Hebei. Link to dynamic distribution map: http://hol.osu.edu/map-large.html?id=275485

### Oxyscelio dermatoglyphes

Burks, 2013

http://lsid.tdwg.org/urn:lsid:biosci.ohio-state.edu:osuc_concepts:275502

urn:lsid:zoobank.org:act:BF3D2E8D-3434-4B3B-88AA-70122522496D

http://species-id.net/wiki/Oxyscelio_dermatoglyphes

Oxyscelio dermatoglyphes Burks et al. 2013: 16, 22, 113. Original description, keyed, placed in *florus* species group.

#### Materials

- **Type status:** Other material. **Occurrence:** catalogNumber: SCAU 200906115; recordedBy: Liu Jing-Xian, et al.; individualCount: 1; sex: male; lifeStage: adult; **Taxon:** taxonID: urn:lsid:biosci.ohio-state.edu:osuc_names:275502; scientificName: Oxyscelio dermatoglyphes; **Location:** country: China; stateProvince: Hainan; locality: Bawangling National Nature Reserve, Hainan Prov., China; locationRemarks: label transliteration: "Hainan, Bawangling, 2006.07.07-11, Liu Jingxian et al."; [海南霸王岭, 2006.07.07-11, 刘经贤等]; decimalLatitude: 19.1167; decimalLongitude: 109.05; georeferenceProtocol: GPS; **Identification:** identifiedBy: Norman F. Johnson; dateIdentified: 2012; **Event:** eventID: urn:lsid:biosci.ohio-state.edu:osuc_occurrences:SCAU__200906115; samplingProtocol: none specified; eventDate: 2006-07-07/11; **Record Level:** modified: 2013-07-17T11:03:18Z; language: en; collectionID: urn:lsid:biocol.org:col:34252; collectionCode: Insects; basisOfRecord: PreservedSpecimen; source: http://hol.osu.edu/spmInfo.html?id=SCAU%20200906115
- **Type status:** Other material. **Occurrence:** catalogNumber: SCAU 2011000087; recordedBy: Chen Hua-Yan; individualCount: 1; sex: male; lifeStage: adult; **Taxon:** taxonID: urn:lsid:biosci.ohio-state.edu:osuc_names:275502; scientificName: Oxyscelio dermatoglyphes; **Location:** country: China; stateProvince: Zhejiang; locality: Tianmushan National Nature Reserve, Mt. Xianrending; verbatimElevation: 1200 m; locationRemarks: label transliteration: "Zhejiang, Tianmushan, Xianrending, 2011.07.25-29, Chen Huayan"; [浙江天目山仙人顶 1200 m, 30°20.56'N 119°26.03'E, 2011.07.25-29, sweeping, 陈华燕]; verbatimCoordinates: 30°20.56'N 119°26.03'E; decimalLatitude: 30.3427; decimalLongitude: 119.4338; georeferenceProtocol: label; **Identification:** identifiedBy: Norman F. Johnson; dateIdentified: 2012; **Event:** eventID: urn:lsid:biosci.ohio-state.edu:osuc_occurrences:SCAU__2011000087; samplingProtocol: sweeping; eventDate: 2011-07-25/29; **Record Level:** modified: 2013-07-17T11:03:34Z; language: en; collectionID: urn:lsid:biocol.org:col:34252; collectionCode: Insects; basisOfRecord: PreservedSpecimen; source: http://hol.osu.edu/spmInfo.html?id=SCAU%202011000087
- **Type status:** Other material. **Occurrence:** catalogNumber: SCAU 2011000082; recordedBy: Chen Hua-Yan; individualCount: 1; sex: male; lifeStage: adult; **Taxon:** taxonID: urn:lsid:biosci.ohio-state.edu:osuc_names:275502; scientificName: Oxyscelio dermatoglyphes; **Location:** country: China; stateProvince: Zhejiang; locality: Tianmushan National Nature Reserve, Mt. Xianrending; verbatimElevation: 1200 m; locationRemarks: label transliteration: "Zhejiang, Tianmushan, Xianrending, 2011.07.25-29, Chen Huayan"; [浙江天目山仙人顶 1200 m, 30°20.56'N 119°26.03'E, 2011.07.25-29, sweeping, 陈华燕]; verbatimCoordinates: 30°20.56'N 119°26.03'E; decimalLatitude: 30.3427; decimalLongitude: 119.4338; georeferenceProtocol: label; **Identification:** identifiedBy: Norman F. Johnson; dateIdentified: 2012; **Event:** eventID: urn:lsid:biosci.ohio-state.edu:osuc_occurrences:SCAU__2011000082; samplingProtocol: sweeping; eventDate: 2011-07-25/29; **Record Level:** modified: 2013-07-17T11:03:33Z; language: en; collectionID: urn:lsid:biocol.org:col:34252; collectionCode: Insects; basisOfRecord: PreservedSpecimen; source: http://hol.osu.edu/spmInfo.html?id=SCAU%202011000082
- **Type status:** Other material. **Occurrence:** catalogNumber: SCAU 2011000080; recordedBy: Chen Hua-Yan; individualCount: 1; sex: male; lifeStage: adult; **Taxon:** taxonID: urn:lsid:biosci.ohio-state.edu:osuc_names:275502; scientificName: Oxyscelio dermatoglyphes; **Location:** country: China; stateProvince: Zhejiang; locality: Tianmushan National Nature Reserve, Mt. Xianrending; verbatimElevation: 1200 m; locationRemarks: label transliteration: "Zhejiang, Tianmushan, Xianrending, 2011.07.25-29, Chen Huayan"; [浙江天目山仙人顶 1200 m, 30°20.56'N 119°26.03'E, 2011.07.25-29, sweeping, 陈华燕]; verbatimCoordinates: 30°20.56'N 119°26.03'E; decimalLatitude: 30.3427; decimalLongitude: 119.4338; georeferenceProtocol: label; **Identification:** identifiedBy: Norman F. Johnson; dateIdentified: 2012; **Event:** eventID: urn:lsid:biosci.ohio-state.edu:osuc_occurrences:SCAU__2011000080; samplingProtocol: sweeping; eventDate: 2011-07-25/29; **Record Level:** modified: 2013-07-17T11:03:32Z; language: en; collectionID: urn:lsid:biocol.org:col:34252; collectionCode: Insects; basisOfRecord: PreservedSpecimen; source: http://hol.osu.edu/spmInfo.html?id=SCAU%202011000080

#### Distribution

This species was known previously only from Taiwan. Its distribution is now seen to include Zhejiang in eastern China and Hainan in the southernmost part of the country. Link to dynamic distribution map: http://hol.osu.edu/map-large.html?id=275502

### Oxyscelio doumao

Burks, 2013

http://lsid.tdwg.org/urn:lsid:biosci.ohio-state.edu:osuc_concepts:305771

urn:lsid:zoobank.org:act:FB6BAA50-D0F3-4F7B-B04E-7AF33F722FBC

http://species-id.net/wiki/Oxyscelio_doumao

Oxyscelio doumao Burks et al. 2013: 15, 23, 117. Original description, keyed, placed in *cuculli* species group.

#### Materials

- **Type status:** Other material. **Occurrence:** catalogNumber: SCAU 200907314; recordedBy: Fan Wu-Qing; individualCount: 1; sex: male; lifeStage: adult; **Taxon:** taxonID: urn:lsid:biosci.ohio-state.edu:osuc_names:305771; scientificName: Oxyscelio doumao; **Location:** country: China; stateProvince: Zhejiang; locality: Mt Fengyang; locationRemarks: label transliteration: "Zhejiang, Fengyangshan, 2003.08.18, Fan Wuqing"; [浙江凤阳山, 2003.08.18, 范武青]; decimalLatitude: 27.9333; decimalLongitude: 119.2; georeferenceProtocol: GPS; **Identification:** identifiedBy: Norman F. Johnson; dateIdentified: 2012; **Event:** eventID: urn:lsid:biosci.ohio-state.edu:osuc_occurrences:SCAU__200907314; samplingProtocol: none specified; eventDate: 2003-08-18; **Record Level:** modified: 2013-07-17T11:03:21Z; language: en; collectionID: urn:lsid:biocol.org:col:34252; collectionCode: Insects; basisOfRecord: PreservedSpecimen; source: http://hol.osu.edu/spmInfo.html?id=SCAU%20200907314
- **Type status:** Other material. **Occurrence:** catalogNumber: SCAU 200905889; recordedBy: Ma Juan-Juan; individualCount: 1; sex: female; lifeStage: adult; **Taxon:** taxonID: urn:lsid:biosci.ohio-state.edu:osuc_names:305771; scientificName: Oxyscelio doumao; **Location:** country: China; stateProvince: Guangdong; locality: Nanling National Nature Reserve; locationRemarks: label transliteration: "Guangdong, Nanling, 2004.07.09-18, Ma Juanjuan"; [广东南岭 2004.07.09-18, 马娟娟]; decimalLatitude: 24.9; decimalLongitude: 113; georeferenceProtocol: GPS; **Identification:** identifiedBy: Norman F. Johnson; dateIdentified: 2012; **Event:** eventID: urn:lsid:biosci.ohio-state.edu:osuc_occurrences:SCAU__200905889; samplingProtocol: none specified; eventDate: 2004-07-09/18; **Record Level:** modified: 2013-07-17T11:03:17Z; language: en; collectionID: urn:lsid:biocol.org:col:34252; collectionCode: Insects; basisOfRecord: PreservedSpecimen; source: http://hol.osu.edu/spmInfo.html?id=SCAU%20200905889
- **Type status:** Other material. **Occurrence:** catalogNumber: SCAU 2011000604; recordedBy: Shi Min; individualCount: 1; sex: female; lifeStage: adult; **Taxon:** taxonID: urn:lsid:biosci.ohio-state.edu:osuc_names:305771; scientificName: Oxyscelio doumao; **Location:** country: China; stateProvince: Shaanxi; locality: Zibaishan National Forest Park; verbatimElevation: 1632 m; locationRemarks: label transliteration: "Shaanxi, Ziboshan, 2004.08.03, Shi Min"; [陕西紫柏山, 1632m, 2004.08.03, 时敏]; decimalLatitude: 33.7089; decimalLongitude: 106.7525; georeferenceProtocol: Google Earth; **Identification:** identifiedBy: Norman F. Johnson; dateIdentified: 2012; **Event:** eventID: urn:lsid:biosci.ohio-state.edu:osuc_occurrences:SCAU__2011000604; samplingProtocol: none specified; eventDate: 2004-08-03; **Record Level:** modified: 2013-07-17T11:03:57Z; language: en; collectionID: urn:lsid:biocol.org:col:34252; collectionCode: Insects; basisOfRecord: PreservedSpecimen; source: http://hol.osu.edu/spmInfo.html?id=SCAU%202011000604
- **Type status:** Other material. **Occurrence:** catalogNumber: SCAU 2011000722; recordedBy: Fan Wu-Qing; individualCount: 1; sex: female; lifeStage: adult; **Taxon:** taxonID: urn:lsid:biosci.ohio-state.edu:osuc_names:305771; scientificName: Oxyscelio doumao; **Location:** country: China; stateProvince: Zhejiang; locality: Mt Fengyang; locationRemarks: label transliteration: "Zhejiang, Fengyangshan, 2008.07.30, Liu Jingxian"; [浙江凤阳山, 1000m, 2008.07.30, 刘经贤]; decimalLatitude: 27.9333; decimalLongitude: 119.2; georeferenceProtocol: GPS; **Identification:** identifiedBy: Norman F. Johnson; dateIdentified: 2012; **Event:** eventID: urn:lsid:biosci.ohio-state.edu:osuc_occurrences:SCAU__2011000722; samplingProtocol: none specified; eventDate: 2008-07-30; **Record Level:** modified: 2013-07-17T11:04:04Z; language: en; collectionID: urn:lsid:biocol.org:col:34252; collectionCode: Insects; basisOfRecord: PreservedSpecimen; source: http://hol.osu.edu/spmInfo.html?id=SCAU%202011000722
- **Type status:** Other material. **Occurrence:** catalogNumber: SCAU 2011000759; recordedBy: Fan Wu-Qing; individualCount: 1; sex: female; lifeStage: adult; **Taxon:** taxonID: urn:lsid:biosci.ohio-state.edu:osuc_names:305771; scientificName: Oxyscelio doumao; **Location:** country: China; stateProvince: Zhejiang; locality: Mt Fengyang; locationRemarks: label transliteration: "Zhejiang, Fengyangshan, 2008.07.30, Liu Jingxian"; [浙江凤阳山, 1000m, 2008.07.30, 刘经贤]; decimalLatitude: 27.9333; decimalLongitude: 119.2; georeferenceProtocol: GPS; **Identification:** identifiedBy: Norman F. Johnson; dateIdentified: 2012; **Event:** eventID: urn:lsid:biosci.ohio-state.edu:osuc_occurrences:SCAU__2011000759; samplingProtocol: none specified; eventDate: 2008-07-30; **Record Level:** modified: 2013-07-17T11:04:05Z; language: en; collectionID: urn:lsid:biocol.org:col:34252; collectionCode: Insects; basisOfRecord: PreservedSpecimen; source: http://hol.osu.edu/spmInfo.html?id=SCAU%202011000759
- **Type status:** Other material. **Occurrence:** catalogNumber: SCAU 2011000159; recordedBy: Xu Zai-Fu, et al.; individualCount: 1; sex: female; lifeStage: adult; **Taxon:** taxonID: urn:lsid:biosci.ohio-state.edu:osuc_names:305771; scientificName: Oxyscelio doumao; **Location:** country: China; stateProvince: Guangxi Zhuang; locality: Longshan Nature Reserve; verbatimElevation: 370 m; locationRemarks: label transliteration: "Guangxi, Longshan Nature Reserve, 2011.07.01-02, Xu Zaifu et al."; [广西龙山自然保护区 370 m, 23°24.727'N 108°31.918'E, 2011.07.01-02, YPT, 许再福等]; verbatimCoordinates: 23°24.727'N 108°31.918'E; decimalLatitude: 23.4121; decimalLongitude: 108.532; georeferenceProtocol: label; **Identification:** identifiedBy: Norman F. Johnson; dateIdentified: 2012; **Event:** eventID: urn:lsid:biosci.ohio-state.edu:osuc_occurrences:SCAU__2011000159; samplingProtocol: yellow pan trap; eventDate: 2011-07-01/02; **Record Level:** modified: 2013-07-17T11:03:46Z; language: en; collectionID: urn:lsid:biocol.org:col:34252; collectionCode: Insects; basisOfRecord: PreservedSpecimen; source: http://hol.osu.edu/spmInfo.html?id=SCAU%202011000159
- **Type status:** Other material. **Occurrence:** catalogNumber: SCAU 2011000079; recordedBy: Chen Hua-Yan; individualCount: 1; sex: male; lifeStage: adult; **Taxon:** taxonID: urn:lsid:biosci.ohio-state.edu:osuc_names:305771; scientificName: Oxyscelio doumao; **Location:** country: China; stateProvince: Zhejiang; locality: Tianmushan National Nature Reserve, Mt. Xianrending; verbatimElevation: 1200 m; locationRemarks: label transliteration: "Zhejiang, Tianmushan, Xianrending, 2011.07.25-29, Chen Huayan"; [浙江天目山仙人顶 1200 m, 30°20.56'N 119°26.03'E, 2011.07.25-29, sweeping, 陈华燕]; verbatimCoordinates: 30°20.56'N 119°26.03'E; decimalLatitude: 30.3427; decimalLongitude: 119.4338; georeferenceProtocol: label; **Identification:** identifiedBy: Norman F. Johnson; dateIdentified: 2012; **Event:** eventID: urn:lsid:biosci.ohio-state.edu:osuc_occurrences:SCAU__2011000079; samplingProtocol: sweeping; eventDate: 2011-07-25/29; **Record Level:** modified: 2013-07-17T11:03:32Z; language: en; collectionID: urn:lsid:biocol.org:col:34252; collectionCode: Insects; basisOfRecord: PreservedSpecimen; source: http://hol.osu.edu/spmInfo.html?id=SCAU%202011000079
- **Type status:** Other material. **Occurrence:** catalogNumber: SCAU 2011000076; recordedBy: Chen Hua-Yan; individualCount: 1; sex: male; lifeStage: adult; **Taxon:** taxonID: urn:lsid:biosci.ohio-state.edu:osuc_names:305771; scientificName: Oxyscelio doumao; **Location:** country: China; stateProvince: Zhejiang; locality: Tianmushan National Nature Reserve, Mt. Xianrending; verbatimElevation: 1200 m; locationRemarks: label transliteration: "Zhejiang, Tianmushan, Xianrending, 2011.07.25-29, Chen Huayan"; [浙江天目山仙人顶 1200 m, 30°20.56'N 119°26.03'E, 2011.07.25-29, sweeping, 陈华燕]; verbatimCoordinates: 30°20.56'N 119°26.03'E; decimalLatitude: 30.3427; decimalLongitude: 119.4338; georeferenceProtocol: label; **Identification:** identifiedBy: Norman F. Johnson; dateIdentified: 2012; **Event:** eventID: urn:lsid:biosci.ohio-state.edu:osuc_occurrences:SCAU__2011000076; samplingProtocol: sweeping; eventDate: 2011-07-25/29; **Record Level:** modified: 2013-07-17T11:03:31Z; language: en; collectionID: urn:lsid:biocol.org:col:34252; collectionCode: Insects; basisOfRecord: PreservedSpecimen; source: http://hol.osu.edu/spmInfo.html?id=SCAU%202011000076
- **Type status:** Other material. **Occurrence:** catalogNumber: SCAU 2011000114; recordedBy: Chen Hua-Yan; individualCount: 1; sex: female; lifeStage: adult; **Taxon:** taxonID: urn:lsid:biosci.ohio-state.edu:osuc_names:305771; scientificName: Oxyscelio doumao; **Location:** country: China; stateProvince: Zhejiang; locality: Tianmushan National Nature Reserve, Mt. Xianrending; verbatimElevation: 1200 m; locationRemarks: label transliteration: "Zhejiang, Tianmushan, Xianrending, 2011.07.25-29, Chen Huayan"; [浙江天目山仙人顶 1200 m, 30°20.56'N 119°26.03'E, 2011.07.25-29, YPT, 陈华燕]; verbatimCoordinates: 30°20.56'N 119°26.03'E; decimalLatitude: 30.3427; decimalLongitude: 119.4338; georeferenceProtocol: label; **Identification:** identifiedBy: Norman F. Johnson; dateIdentified: 2012; **Event:** eventID: urn:lsid:biosci.ohio-state.edu:osuc_occurrences:SCAU__2011000114; samplingProtocol: yellow pan trap; eventDate: 2011-07-25/29; **Record Level:** modified: 2013-07-17T11:03:41Z; language: en; collectionID: urn:lsid:biocol.org:col:34252; collectionCode: Insects; basisOfRecord: PreservedSpecimen; source: http://hol.osu.edu/spmInfo.html?id=SCAU%202011000114
- **Type status:** Other material. **Occurrence:** catalogNumber: SCAU 2011000113; recordedBy: Chen Hua-Yan; individualCount: 1; sex: female; lifeStage: adult; **Taxon:** taxonID: urn:lsid:biosci.ohio-state.edu:osuc_names:305771; scientificName: Oxyscelio doumao; **Location:** country: China; stateProvince: Zhejiang; locality: Tianmushan National Nature Reserve, Mt. Xianrending; verbatimElevation: 1200 m; locationRemarks: label transliteration: "Zhejiang, Tianmushan, Xianrending, 2011.07.25-29, Chen Huayan"; [浙江天目山仙人顶 1200 m, 30°20.56'N 119°26.03'E, 2011.07.25-29, YPT, 陈华燕]; verbatimCoordinates: 30°20.56'N 119°26.03'E; decimalLatitude: 30.3427; decimalLongitude: 119.4338; georeferenceProtocol: label; **Identification:** identifiedBy: Norman F. Johnson; dateIdentified: 2012; **Event:** eventID: urn:lsid:biosci.ohio-state.edu:osuc_occurrences:SCAU__2011000113; samplingProtocol: yellow pan trap; eventDate: 2011-07-25/29; **Record Level:** modified: 2013-07-17T11:03:41Z; language: en; collectionID: urn:lsid:biocol.org:col:34252; collectionCode: Insects; basisOfRecord: PreservedSpecimen; source: http://hol.osu.edu/spmInfo.html?id=SCAU%202011000113
- **Type status:** Other material. **Occurrence:** catalogNumber: SCAU 2011000110; recordedBy: Chen Hua-Yan; individualCount: 1; sex: female; lifeStage: adult; **Taxon:** taxonID: urn:lsid:biosci.ohio-state.edu:osuc_names:305771; scientificName: Oxyscelio doumao; **Location:** country: China; stateProvince: Zhejiang; locality: Tianmushan National Nature Reserve, Mt. Xianrending; verbatimElevation: 1200 m; locationRemarks: label transliteration: "Zhejiang, Tianmushan, Xianrending, 2011.07.25-29, Chen Huayan"; [浙江天目山仙人顶 1200 m, 30°20.56'N 119°26.03'E, 2011.07.25-29, YPT, 陈华燕]; verbatimCoordinates: 30°20.56'N 119°26.03'E; decimalLatitude: 30.3427; decimalLongitude: 119.4338; georeferenceProtocol: label; **Identification:** identifiedBy: Norman F. Johnson; dateIdentified: 2012; **Event:** eventID: urn:lsid:biosci.ohio-state.edu:osuc_occurrences:SCAU__2011000110; samplingProtocol: yellow pan trap; eventDate: 2011-07-25/29; **Record Level:** modified: 2013-07-17T11:03:40Z; language: en; collectionID: urn:lsid:biocol.org:col:34252; collectionCode: Insects; basisOfRecord: PreservedSpecimen; source: http://hol.osu.edu/spmInfo.html?id=SCAU%202011000110
- **Type status:** Other material. **Occurrence:** catalogNumber: SCAU 2011000049; recordedBy: Jin Cheng-Yuan; individualCount: 1; sex: male; lifeStage: adult; **Taxon:** taxonID: urn:lsid:biosci.ohio-state.edu:osuc_names:305771; scientificName: Oxyscelio doumao; **Location:** country: China; stateProvince: Zhejiang; locality: Tianmushan National Nature Reserve, Mt. Xianrending; verbatimElevation: 1200 m; locationRemarks: label transliteration: "Zhejiang, Tianmushan, Xianrending, 2011.07.25-29, Jin Chengyuan"; [浙江天目山仙人顶 1200 m, 30°20.56'N 119°26.03'E, 2011.07.25-29, sweeping, 金程远]; verbatimCoordinates: 30°20.56'N 119°26.03'E; decimalLatitude: 30.3427; decimalLongitude: 119.4338; georeferenceProtocol: label; **Identification:** identifiedBy: Norman F. Johnson; dateIdentified: 2012; **Event:** eventID: urn:lsid:biosci.ohio-state.edu:osuc_occurrences:SCAU__2011000049; samplingProtocol: sweeping; eventDate: 2011-07-25/29; **Record Level:** modified: 2013-07-17T11:03:28Z; language: en; collectionID: urn:lsid:biocol.org:col:34252; collectionCode: Insects; basisOfRecord: PreservedSpecimen; source: http://hol.osu.edu/spmInfo.html?id=SCAU%202011000049
- **Type status:** Other material. **Occurrence:** catalogNumber: SCAU 2011000118; recordedBy: Chen Hua-Yan; individualCount: 1; sex: female; lifeStage: adult; **Taxon:** taxonID: urn:lsid:biosci.ohio-state.edu:osuc_names:305771; scientificName: Oxyscelio doumao; **Location:** country: China; stateProvince: Zhejiang; locality: Tianmushan National Nature Reserve, Mt. Xianrending; verbatimElevation: 1200 m; locationRemarks: label transliteration: "Zhejiang, Tianmushan, Xianrending, 2011.07.25-29, Chen Huayan"; [浙江天目山仙人顶 1200 m, 30°20.56'N 119°26.03'E, 2011.07.25-29, YPT, 陈华燕]; verbatimCoordinates: 30°20.56'N 119°26.03'E; decimalLatitude: 30.3427; decimalLongitude: 119.4338; georeferenceProtocol: label; **Identification:** identifiedBy: Norman F. Johnson; dateIdentified: 2012; **Event:** eventID: urn:lsid:biosci.ohio-state.edu:osuc_occurrences:SCAU__2011000118; samplingProtocol: yellow pan trap; eventDate: 2011-07-25/29; **Record Level:** modified: 2013-07-17T11:03:42Z; language: en; collectionID: urn:lsid:biocol.org:col:34252; collectionCode: Insects; basisOfRecord: PreservedSpecimen; source: http://hol.osu.edu/spmInfo.html?id=SCAU%202011000118
- **Type status:** Other material. **Occurrence:** catalogNumber: SCAU 2011000097; recordedBy: Jin Cheng-Yuan; individualCount: 1; sex: female; lifeStage: adult; **Taxon:** taxonID: urn:lsid:biosci.ohio-state.edu:osuc_names:305771; scientificName: Oxyscelio doumao; **Location:** country: China; stateProvince: Zhejiang; locality: Tianmushan National Nature Reserve, Mt. Xianrending; verbatimElevation: 1200 m; locationRemarks: label transliteration: "Zhejiang, Tianmushan, Xianrending, 2011.07.25-29, Jin Chengyuan"; [浙江天目山仙人顶 1200 m, 30°20.56'N 119°26.03'E, 2011.07.25-29, YPT, 金程远]; verbatimCoordinates: 30°20.56'N 119°26.03'E; decimalLatitude: 30.3427; decimalLongitude: 119.4338; georeferenceProtocol: label; **Identification:** identifiedBy: Norman F. Johnson; dateIdentified: 2012; **Event:** eventID: urn:lsid:biosci.ohio-state.edu:osuc_occurrences:SCAU__2011000097; samplingProtocol: yellow pan trap; eventDate: 2011-07-25/29; **Record Level:** modified: 2013-07-17T11:03:37Z; language: en; collectionID: urn:lsid:biocol.org:col:34252; collectionCode: Insects; basisOfRecord: PreservedSpecimen; source: http://hol.osu.edu/spmInfo.html?id=SCAU%202011000097

#### Distribution

*Oxyscelio doumao* is known only from China. When originally described it was known only from Sichuan. The new data indicate that it is much more widespread and is found in Shaanxi, Guangxi, Guangdong and Zhejiang. Link to dynamic distribution map: http://hol.osu.edu/map-large.html?id=305771

### Oxyscelio florus

Kononova, 2007

http://lsid.tdwg.org/urn:lsid:biosci.ohio-state.edu:osuc_concepts:243848

urn:lsid:zoobank.org:act:6836FE9A-1218-4498-B609-A99CA8539908

http://species-id.net/wiki/Oxyscelio_florus

Oxyscelio florum Kononova and Fursov 2007: 62. Original description.

#### Materials

- **Type status:** Other material. **Occurrence:** catalogNumber: SCAU 200907308; recordedBy: Fan Wu-Qing; individualCount: 1; sex: female; lifeStage: adult; **Taxon:** taxonID: urn:lsid:biosci.ohio-state.edu:osuc_names:243848; scientificName: Oxyscelio florus; **Location:** country: China; stateProvince: Zhejiang; locality: Mt Fengyang; locationRemarks: label transliteration: "Zhejiang, Fengyangshan, 2003.08.07, Fan Wuqing"; [浙江凤阳山, 2003.08.07, 范武青]; decimalLatitude: 27.9333; decimalLongitude: 119.2; georeferenceProtocol: GPS; **Identification:** identifiedBy: Norman F. Johnson; dateIdentified: 2012; **Event:** eventID: urn:lsid:biosci.ohio-state.edu:osuc_occurrences:SCAU__200907308; samplingProtocol: none specified; eventDate: 2003-08-07; **Record Level:** modified: 2013-07-17T11:03:20Z; language: en; collectionID: urn:lsid:biocol.org:col:34252; collectionCode: Insects; basisOfRecord: PreservedSpecimen; source: http://hol.osu.edu/spmInfo.html?id=SCAU%20200907308
- **Type status:** Other material. **Occurrence:** catalogNumber: SCAU 200907355; recordedBy: Liu Jing-Xian; individualCount: 1; sex: female; lifeStage: adult; **Taxon:** taxonID: urn:lsid:biosci.ohio-state.edu:osuc_names:243848; scientificName: Oxyscelio florus; **Location:** country: China; stateProvince: Zhejiang; locality: Mt Baishanzu; locationRemarks: label transliteration: "Zhejiang, Baishanzu, 2003.08.15, Liu Jingxian"; [浙江百山祖 2003.08.15 刘经贤]; decimalLatitude: 27.75; decimalLongitude: 119.2; georeferenceProtocol: GPS; **Identification:** identifiedBy: Norman F. Johnson; dateIdentified: 2012; **Event:** eventID: urn:lsid:biosci.ohio-state.edu:osuc_occurrences:SCAU__200907355; samplingProtocol: none specified; eventDate: 2003-08-15; **Record Level:** modified: 2013-07-17T11:03:21Z; language: en; collectionID: urn:lsid:biocol.org:col:34252; collectionCode: Insects; basisOfRecord: PreservedSpecimen; source: http://hol.osu.edu/spmInfo.html?id=SCAU%20200907355
- **Type status:** Other material. **Occurrence:** catalogNumber: SCAU 200907363; recordedBy: Liu Jing-Xian; individualCount: 1; sex: female; lifeStage: adult; **Taxon:** taxonID: urn:lsid:biosci.ohio-state.edu:osuc_names:243848; scientificName: Oxyscelio florus; **Location:** country: China; stateProvince: Zhejiang; locality: Mt Baishanzu; locationRemarks: label transliteration: "Zhejiang, Baishanzu, 2003.08.15, Liu Jingxian"; [浙江百山祖 2003.08.15 刘经贤]; decimalLatitude: 27.75; decimalLongitude: 119.2; georeferenceProtocol: GPS; **Identification:** identifiedBy: Norman F. Johnson; dateIdentified: 2012; **Event:** eventID: urn:lsid:biosci.ohio-state.edu:osuc_occurrences:SCAU__200907363; samplingProtocol: none specified; eventDate: 2003-08-15; **Record Level:** modified: 2013-07-17T11:03:21Z; language: en; collectionID: urn:lsid:biocol.org:col:34252; collectionCode: Insects; basisOfRecord: PreservedSpecimen; source: http://hol.osu.edu/spmInfo.html?id=SCAU%20200907363
- **Type status:** Other material. **Occurrence:** catalogNumber: SCAU 200909191; recordedBy: Xu Zai-Fu; individualCount: 1; sex: female; lifeStage: adult; **Taxon:** taxonID: urn:lsid:biosci.ohio-state.edu:osuc_names:243848; scientificName: Oxyscelio florus; **Location:** country: China; stateProvince: Guangdong; locality: Nanling National Nature Reserve; locationRemarks: label transliteration: "Guangdong, Nanling, 2004.02.04, Xu Zaifu"; [广东南岭 2004.02.04, 许再福]; decimalLatitude: 24.9; decimalLongitude: 113; georeferenceProtocol: GPS; **Identification:** identifiedBy: Norman F. Johnson; dateIdentified: 2012; **Event:** eventID: urn:lsid:biosci.ohio-state.edu:osuc_occurrences:SCAU__200909191; samplingProtocol: none specified; eventDate: 2004-02-04; **Record Level:** modified: 2013-07-17T11:03:22Z; language: en; collectionID: urn:lsid:biocol.org:col:34252; collectionCode: Insects; basisOfRecord: PreservedSpecimen; source: http://hol.osu.edu/spmInfo.html?id=SCAU%20200909191
- **Type status:** Other material. **Occurrence:** catalogNumber: SCAU 200909011; recordedBy: Liu Jing-Xian; individualCount: 1; sex: female; lifeStage: adult; **Taxon:** taxonID: urn:lsid:biosci.ohio-state.edu:osuc_names:243848; scientificName: Oxyscelio florus; **Location:** country: China; stateProvince: Guangdong; locality: Nanling National Nature Reserve; locationRemarks: label transliteration: "Guangdong, Nanling, 2004.08.04, Liu Jingxian"; [广东南岭, 2004.08.04, 刘经贤等]; decimalLatitude: 24.9; decimalLongitude: 113; georeferenceProtocol: GPS; **Identification:** identifiedBy: Norman F. Johnson; dateIdentified: 2012; **Event:** eventID: urn:lsid:biosci.ohio-state.edu:osuc_occurrences:SCAU__200909011; samplingProtocol: none specified; eventDate: 2004-08-04; **Record Level:** modified: 2013-07-17T11:03:22Z; language: en; collectionID: urn:lsid:biocol.org:col:34252; collectionCode: Insects; basisOfRecord: PreservedSpecimen; source: http://hol.osu.edu/spmInfo.html?id=SCAU%20200909011
- **Type status:** Other material. **Occurrence:** catalogNumber: SCAU 2011000468; recordedBy: Shi Min; individualCount: 1; sex: female; lifeStage: adult; **Taxon:** taxonID: urn:lsid:biosci.ohio-state.edu:osuc_names:243848; scientificName: Oxyscelio florus; **Location:** country: China; stateProvince: Zhejiang; locality: Mt Qingliangfeng; locationRemarks: label transliteration: "Zhejiang, Qingliangfeng, 2005.08.09, Shi Min"; [浙江清凉峰 2005.08.09 时敏]; decimalLatitude: 30.0703; decimalLongitude: 118.8944; georeferenceProtocol: Google Earth; georeferenceRemarks: GPS coords. adjusted to place within Zhejiang Prov.; **Identification:** identifiedBy: Norman F. Johnson; dateIdentified: 2012; **Event:** eventID: urn:lsid:biosci.ohio-state.edu:osuc_occurrences:SCAU__2011000468; samplingProtocol: none specified; eventDate: 2005-08-09; **Record Level:** modified: 2013-07-17T11:03:53Z; language: en; collectionID: urn:lsid:biocol.org:col:34252; collectionCode: Insects; basisOfRecord: PreservedSpecimen; source: http://hol.osu.edu/spmInfo.html?id=SCAU%202011000468
- **Type status:** Other material. **Occurrence:** catalogNumber: SCAU 2011000466; recordedBy: Shi Min; individualCount: 1; sex: female; lifeStage: adult; **Taxon:** taxonID: urn:lsid:biosci.ohio-state.edu:osuc_names:243848; scientificName: Oxyscelio florus; **Location:** country: China; stateProvince: Zhejiang; locality: Mt Qingliangfeng; locationRemarks: label transliteration: "Zhejiang, Qingliangfeng, 2005.08.09, Shi Min"; [浙江清凉峰 2005.08.09 时敏]; decimalLatitude: 30.0703; decimalLongitude: 118.8944; georeferenceProtocol: Google Earth; georeferenceRemarks: GPS coords. adjusted to place within Zhejiang Prov.; **Identification:** identifiedBy: Norman F. Johnson; dateIdentified: 2012; **Event:** eventID: urn:lsid:biosci.ohio-state.edu:osuc_occurrences:SCAU__2011000466; samplingProtocol: none specified; eventDate: 2005-08-09; **Record Level:** modified: 2013-07-17T11:03:53Z; language: en; collectionID: urn:lsid:biocol.org:col:34252; collectionCode: Insects; basisOfRecord: PreservedSpecimen; source: http://hol.osu.edu/spmInfo.html?id=SCAU%202011000466
- **Type status:** Other material. **Occurrence:** catalogNumber: SCAU 2011000628; recordedBy: Zhang Hong-Ying; individualCount: 1; sex: female; lifeStage: adult; **Taxon:** taxonID: urn:lsid:biosci.ohio-state.edu:osuc_names:243848; scientificName: Oxyscelio florus; **Location:** country: China; stateProvince: Zhejiang; locality: Mt Qingliangfeng; locationRemarks: label transliteration: "Zhejiang, Qingliangfeng, 2005.08.09, Zhang Hongying"; [浙江清凉峰 2005.08.09 张红英]; decimalLatitude: 30.0703; decimalLongitude: 118.8944; georeferenceProtocol: Google Earth; georeferenceRemarks: GPS coords. adjusted to place within Zhejiang Prov.; **Identification:** identifiedBy: Norman F. Johnson; dateIdentified: 2012; **Event:** eventID: urn:lsid:biosci.ohio-state.edu:osuc_occurrences:SCAU__2011000628; samplingProtocol: none specified; eventDate: 2005-08-09; **Record Level:** modified: 2013-07-17T11:04:00Z; language: en; collectionID: urn:lsid:biocol.org:col:34252; collectionCode: Insects; basisOfRecord: PreservedSpecimen; source: http://hol.osu.edu/spmInfo.html?id=SCAU%202011000628
- **Type status:** Other material. **Occurrence:** catalogNumber: SCAU 2011000629; recordedBy: Zhang Hong-Ying; individualCount: 1; sex: female; lifeStage: adult; **Taxon:** taxonID: urn:lsid:biosci.ohio-state.edu:osuc_names:243848; scientificName: Oxyscelio florus; **Location:** country: China; stateProvince: Zhejiang; locality: Mt Qingliangfeng; locationRemarks: label transliteration: "Zhejiang, Qingliangfeng, 2005.08.09, Zhang Hongying"; [浙江清凉峰 2005.08.09 张红英]; decimalLatitude: 30.0703; decimalLongitude: 118.8944; georeferenceProtocol: Google Earth; georeferenceRemarks: GPS coords. adjusted to place within Zhejiang Prov.; **Identification:** identifiedBy: Norman F. Johnson; dateIdentified: 2012; **Event:** eventID: urn:lsid:biosci.ohio-state.edu:osuc_occurrences:SCAU__2011000629; samplingProtocol: none specified; eventDate: 2005-08-09; **Record Level:** modified: 2013-07-17T11:04:00Z; language: en; collectionID: urn:lsid:biocol.org:col:34252; collectionCode: Insects; basisOfRecord: PreservedSpecimen; source: http://hol.osu.edu/spmInfo.html?id=SCAU%202011000629
- **Type status:** Other material. **Occurrence:** catalogNumber: SCAU 2011000630; recordedBy: Zhang Hong-Ying; individualCount: 1; sex: female; lifeStage: adult; **Taxon:** taxonID: urn:lsid:biosci.ohio-state.edu:osuc_names:243848; scientificName: Oxyscelio florus; **Location:** country: China; stateProvince: Zhejiang; locality: Mt Qingliangfeng; locationRemarks: label transliteration: "Zhejiang, Qingliangfeng, 2005.08.09, Zhang Hongying"; [浙江清凉峰 2005.08.09 张红英]; decimalLatitude: 30.0703; decimalLongitude: 118.8944; georeferenceProtocol: Google Earth; georeferenceRemarks: GPS coords. adjusted to place within Zhejiang Prov.; **Identification:** identifiedBy: Norman F. Johnson; dateIdentified: 2012; **Event:** eventID: urn:lsid:biosci.ohio-state.edu:osuc_occurrences:SCAU__2011000630; samplingProtocol: none specified; eventDate: 2005-08-09; **Record Level:** modified: 2013-07-17T11:04:00Z; language: en; collectionID: urn:lsid:biocol.org:col:34252; collectionCode: Insects; basisOfRecord: PreservedSpecimen; source: http://hol.osu.edu/spmInfo.html?id=SCAU%202011000630
- **Type status:** Other material. **Occurrence:** catalogNumber: SCAU 2011000632; recordedBy: Zhang Hong-Ying; individualCount: 1; sex: female; lifeStage: adult; **Taxon:** taxonID: urn:lsid:biosci.ohio-state.edu:osuc_names:243848; scientificName: Oxyscelio florus; **Location:** country: China; stateProvince: Zhejiang; locality: Mt Qingliangfeng; locationRemarks: label transliteration: "Zhejiang, Qingliangfeng, 2005.08.09, Zhang Hongying"; [浙江清凉峰 2005.08.09 张红英]; decimalLatitude: 30.0703; decimalLongitude: 118.8944; georeferenceProtocol: Google Earth; georeferenceRemarks: GPS coords. adjusted to place within Zhejiang Prov.; **Identification:** identifiedBy: Norman F. Johnson; dateIdentified: 2012; **Event:** eventID: urn:lsid:biosci.ohio-state.edu:osuc_occurrences:SCAU__2011000632; samplingProtocol: none specified; eventDate: 2005-08-09; **Record Level:** modified: 2013-07-17T11:04:01Z; language: en; collectionID: urn:lsid:biocol.org:col:34252; collectionCode: Insects; basisOfRecord: PreservedSpecimen; source: http://hol.osu.edu/spmInfo.html?id=SCAU%202011000632
- **Type status:** Other material. **Occurrence:** catalogNumber: SCAU 2011000634; recordedBy: Zhang Hong-Ying; individualCount: 1; sex: female; lifeStage: adult; **Taxon:** taxonID: urn:lsid:biosci.ohio-state.edu:osuc_names:243848; scientificName: Oxyscelio florus; **Location:** country: China; stateProvince: Zhejiang; locality: Mt Qingliangfeng; locationRemarks: label transliteration: "Zhejiang, Qingliangfeng, 2005.08.09, Zhang Hongying"; [浙江清凉峰 2005.08.09 张红英]; decimalLatitude: 30.0703; decimalLongitude: 118.8944; georeferenceProtocol: Google Earth; georeferenceRemarks: GPS coords. adjusted to place within Zhejiang Prov.; **Identification:** identifiedBy: Norman F. Johnson; dateIdentified: 2012; **Event:** eventID: urn:lsid:biosci.ohio-state.edu:osuc_occurrences:SCAU__2011000634; samplingProtocol: none specified; eventDate: 2005-08-09; **Record Level:** modified: 2013-07-17T11:04:01Z; language: en; collectionID: urn:lsid:biocol.org:col:34252; collectionCode: Insects; basisOfRecord: PreservedSpecimen; source: http://hol.osu.edu/spmInfo.html?id=SCAU%202011000634
- **Type status:** Other material. **Occurrence:** catalogNumber: SCAU 2011000510; recordedBy: Zhang Hong-Ying; individualCount: 1; sex: female; lifeStage: adult; **Taxon:** taxonID: urn:lsid:biosci.ohio-state.edu:osuc_names:243848; scientificName: Oxyscelio florus; **Location:** country: China; stateProvince: Zhejiang; locality: Mt Qingliangfeng; locationRemarks: label transliteration: "Zhejiang, Qingliangfeng, 2005.08.10, Zhang Hongying"; [浙江清凉峰 2005.08.10 张红英]; decimalLatitude: 30.0703; decimalLongitude: 118.8944; georeferenceProtocol: Google Earth; georeferenceRemarks: GPS coords. adjusted to place within Zhejiang Prov.; **Identification:** identifiedBy: Norman F. Johnson; dateIdentified: 2012; **Event:** eventID: urn:lsid:biosci.ohio-state.edu:osuc_occurrences:SCAU__2011000510; samplingProtocol: none specified; eventDate: 2005-08-10; **Record Level:** modified: 2013-07-17T11:03:55Z; language: en; collectionID: urn:lsid:biocol.org:col:34252; collectionCode: Insects; basisOfRecord: PreservedSpecimen; source: http://hol.osu.edu/spmInfo.html?id=SCAU%202011000510
- **Type status:** Other material. **Occurrence:** catalogNumber: SCAU 2011000736; recordedBy: Shi Min; individualCount: 1; sex: female; lifeStage: adult; **Taxon:** taxonID: urn:lsid:biosci.ohio-state.edu:osuc_names:243848; scientificName: Oxyscelio florus; **Location:** country: China; stateProvince: Zhejiang; locality: Mt Qingliangfeng; locationRemarks: label transliteration: "Zhejiang, Qingliangfeng, 2005.08.10, Shi Min"; [浙江清凉峰 2005.08.10 时敏]; decimalLatitude: 30.0703; decimalLongitude: 118.8944; georeferenceProtocol: Google Earth; georeferenceRemarks: GPS coords. adjusted to place within Zhejiang Prov.; **Identification:** identifiedBy: Norman F. Johnson; dateIdentified: 2012; **Event:** eventID: urn:lsid:biosci.ohio-state.edu:osuc_occurrences:SCAU__2011000736; samplingProtocol: none specified; eventDate: 2005-08-10; **Record Level:** modified: 2013-07-17T11:04:05Z; language: en; collectionID: urn:lsid:biocol.org:col:34252; collectionCode: Insects; basisOfRecord: PreservedSpecimen; source: http://hol.osu.edu/spmInfo.html?id=SCAU%202011000736
- **Type status:** Other material. **Occurrence:** catalogNumber: SCAU 2011000420; recordedBy: Shi Min; individualCount: 1; sex: female; lifeStage: adult; **Taxon:** taxonID: urn:lsid:biosci.ohio-state.edu:osuc_names:243848; scientificName: Oxyscelio florus; **Location:** country: China; stateProvince: Hebei; county: Zhangjiakou; locality: Mt Dongling; locationRemarks: label transliteration: "Hebei, Zhang jia kou, Donglingshan, 2005.08.11, Shi Min"; [河北张家口东灵山, 2005.08.11, 时敏]; decimalLatitude: 40.0333; decimalLongitude: 115.45; georeferenceProtocol: GPS; **Identification:** identifiedBy: Norman F. Johnson; dateIdentified: 2012; **Event:** eventID: urn:lsid:biosci.ohio-state.edu:osuc_occurrences:SCAU__2011000420; samplingProtocol: none specified; eventDate: 2005-08-11; **Record Level:** modified: 2013-07-17T11:03:51Z; language: en; collectionID: urn:lsid:biocol.org:col:34252; collectionCode: Insects; basisOfRecord: PreservedSpecimen; source: http://hol.osu.edu/spmInfo.html?id=SCAU%202011000420
- **Type status:** Other material. **Occurrence:** catalogNumber: SCAU 2011000578; recordedBy: Shi Min; individualCount: 1; sex: female; lifeStage: adult; **Taxon:** taxonID: urn:lsid:biosci.ohio-state.edu:osuc_names:243848; scientificName: Oxyscelio florus; **Location:** country: China; stateProvince: Zhejiang; locality: Mt Qingliangfeng; locationRemarks: label transliteration: "Zhejiang, Qingliangfeng, 2005.08.11, Shi Min"; [浙江清凉峰 2005.08.11 时敏]; decimalLatitude: 30.0703; decimalLongitude: 118.8944; georeferenceProtocol: Google Earth; georeferenceRemarks: GPS coords. adjusted to place within Zhejiang Prov.; **Identification:** identifiedBy: Norman F. Johnson; dateIdentified: 2012; **Event:** eventID: urn:lsid:biosci.ohio-state.edu:osuc_occurrences:SCAU__2011000578; samplingProtocol: none specified; eventDate: 2005-08-11; **Record Level:** modified: 2013-07-17T11:03:55Z; language: en; collectionID: urn:lsid:biocol.org:col:34252; collectionCode: Insects; basisOfRecord: PreservedSpecimen; source: http://hol.osu.edu/spmInfo.html?id=SCAU%202011000578
- **Type status:** Other material. **Occurrence:** catalogNumber: SCAU 2011000737; recordedBy: Zhang Hong-Ying; individualCount: 1; sex: female; lifeStage: adult; **Taxon:** taxonID: urn:lsid:biosci.ohio-state.edu:osuc_names:243848; scientificName: Oxyscelio florus; **Location:** country: China; stateProvince: Zhejiang; locality: Mt Qingliangfeng; locationRemarks: label transliteration: "Zhejiang, Qingliangfeng, 2005.08.12, Zhang Hongying"; [浙江清凉峰 2005.08.12 张红英]; decimalLatitude: 30.0703; decimalLongitude: 118.8944; georeferenceProtocol: Google Earth; georeferenceRemarks: GPS coords. adjusted to place within Zhejiang Prov.; **Identification:** identifiedBy: Norman F. Johnson; dateIdentified: 2012; **Event:** eventID: urn:lsid:biosci.ohio-state.edu:osuc_occurrences:SCAU__2011000737; samplingProtocol: none specified; eventDate: 2005-08-12; **Record Level:** modified: 2013-07-17T11:04:05Z; language: en; collectionID: urn:lsid:biocol.org:col:34252; collectionCode: Insects; basisOfRecord: PreservedSpecimen; source: http://hol.osu.edu/spmInfo.html?id=SCAU%202011000737
- **Type status:** Other material. **Occurrence:** catalogNumber: SCAU 200907945; recordedBy: Xu Zai-Fu, et al.; individualCount: 1; sex: female; lifeStage: adult; **Taxon:** taxonID: urn:lsid:biosci.ohio-state.edu:osuc_names:243848; scientificName: Oxyscelio florus; **Location:** country: China; stateProvince: Guangdong; locality: Chebaling National Nature Reserve; locationRemarks: label transliteration: "Guangdong, Chebaling, 2008.07.23-28, Xu Zaifu et al."; [广东车八岭, 2008.07.23-28, 许再福等]; decimalLatitude: 24.7167; decimalLongitude: 114.2333; georeferenceProtocol: GPS; **Identification:** identifiedBy: Norman F. Johnson; dateIdentified: 2012; **Event:** eventID: urn:lsid:biosci.ohio-state.edu:osuc_occurrences:SCAU__200907945; samplingProtocol: none specified; eventDate: 2008-07-23/28; **Record Level:** modified: 2013-07-17T11:03:21Z; language: en; collectionID: urn:lsid:biocol.org:col:34252; collectionCode: Insects; basisOfRecord: PreservedSpecimen; source: http://hol.osu.edu/spmInfo.html?id=SCAU%20200907945
- **Type status:** Other material. **Occurrence:** catalogNumber: SCAU 200907966; recordedBy: Xu Zai-Fu, et al.; individualCount: 1; sex: female; lifeStage: adult; **Taxon:** taxonID: urn:lsid:biosci.ohio-state.edu:osuc_names:243848; scientificName: Oxyscelio florus; **Location:** country: China; stateProvince: Guangdong; locality: Chebaling National Nature Reserve; locationRemarks: label transliteration: "Guangdong, Chebaling, 2008.07.23-28, Xu Zaifu et al."; [广东车八岭, 2008.07.23-28, 许再福等]; decimalLatitude: 24.7167; decimalLongitude: 114.2333; georeferenceProtocol: GPS; **Identification:** identifiedBy: Norman F. Johnson; dateIdentified: 2012; **Event:** eventID: urn:lsid:biosci.ohio-state.edu:osuc_occurrences:SCAU__200907966; samplingProtocol: none specified; eventDate: 2008-07-23/28; **Record Level:** modified: 2013-07-17T11:03:22Z; language: en; collectionID: urn:lsid:biocol.org:col:34252; collectionCode: Insects; basisOfRecord: PreservedSpecimen; source: http://hol.osu.edu/spmInfo.html?id=SCAU%20200907966
- **Type status:** Other material. **Occurrence:** catalogNumber: SCAU 2010100360; recordedBy: Chen Hua-Yan; individualCount: 1; sex: female; lifeStage: adult; **Taxon:** taxonID: urn:lsid:biosci.ohio-state.edu:osuc_names:243848; scientificName: Oxyscelio florus; **Location:** country: China; stateProvince: Guangdong; locality: Nanling National Nature Reserve; locationRemarks: label transliteration: "Guangdong, Nanling, 2010.08.08-17, Chen Huayan"; [广东南岭, 2010.08.08-17, 陈华燕]; decimalLatitude: 24.9; decimalLongitude: 113; georeferenceProtocol: GPS; **Identification:** identifiedBy: Norman F. Johnson; dateIdentified: 2012; **Event:** eventID: urn:lsid:biosci.ohio-state.edu:osuc_occurrences:SCAU__2010100360; samplingProtocol: none specified; eventDate: 2010-08-08/17; **Record Level:** modified: 2013-07-17T11:03:26Z; language: en; collectionID: urn:lsid:biocol.org:col:34252; collectionCode: Insects; basisOfRecord: PreservedSpecimen; source: http://hol.osu.edu/spmInfo.html?id=SCAU%202010100360
- **Type status:** Other material. **Occurrence:** catalogNumber: SCAU 2010100359; recordedBy: Chen Hua-Yan; individualCount: 1; sex: female; lifeStage: adult; **Taxon:** taxonID: urn:lsid:biosci.ohio-state.edu:osuc_names:243848; scientificName: Oxyscelio florus; **Location:** country: China; stateProvince: Guangdong; locality: Nanling National Nature Reserve; locationRemarks: label transliteration: "Guangdong, Nanling, 2010.08.08-17, Chen Huayan"; [广东南岭, 2010.08.08-17, 陈华燕]; decimalLatitude: 24.9; decimalLongitude: 113; georeferenceProtocol: GPS; **Identification:** identifiedBy: Norman F. Johnson; dateIdentified: 2012; **Event:** eventID: urn:lsid:biosci.ohio-state.edu:osuc_occurrences:SCAU__2010100359; samplingProtocol: none specified; eventDate: 2010-08-08/17; **Record Level:** modified: 2013-07-17T11:03:26Z; language: en; collectionID: urn:lsid:biocol.org:col:34252; collectionCode: Insects; basisOfRecord: PreservedSpecimen; source: http://hol.osu.edu/spmInfo.html?id=SCAU%202010100359
- **Type status:** Other material. **Occurrence:** catalogNumber: SCAU 2010100355; recordedBy: Chen Hua-Yan; individualCount: 1; sex: female; lifeStage: adult; **Taxon:** taxonID: urn:lsid:biosci.ohio-state.edu:osuc_names:243848; scientificName: Oxyscelio florus; **Location:** country: China; stateProvince: Guangdong; locality: Nanling National Nature Reserve; locationRemarks: label transliteration: "Guangdong, Nanling, 2010.08.08-17, Chen Huayan"; [广东南岭, 2010.08.08-17, 陈华燕]; decimalLatitude: 24.9; decimalLongitude: 113; georeferenceProtocol: GPS; **Identification:** identifiedBy: Norman F. Johnson; dateIdentified: 2012; **Event:** eventID: urn:lsid:biosci.ohio-state.edu:osuc_occurrences:SCAU__2010100355; samplingProtocol: none specified; eventDate: 2010-08-08/17; **Record Level:** modified: 2013-07-17T11:03:25Z; language: en; collectionID: urn:lsid:biocol.org:col:34252; collectionCode: Insects; basisOfRecord: PreservedSpecimen; source: http://hol.osu.edu/spmInfo.html?id=SCAU%202010100355
- **Type status:** Other material. **Occurrence:** catalogNumber: SCAU TEMP; recordedBy: Chen Hua-Yan; individualCount: 1; sex: female; lifeStage: adult; **Taxon:** taxonID: urn:lsid:biosci.ohio-state.edu:osuc_names:243848; scientificName: Oxyscelio florus; **Location:** country: China; stateProvince: Guangdong; locality: Nanling National Nature Reserve; locationRemarks: label transliteration: "Guangdong, Nanling, 2010.08.08-17, Chen Huayan"; [广东南岭自然保护区, 2010.08.08-17, 陈华燕]; decimalLatitude: 24.9; decimalLongitude: 113; georeferenceProtocol: GPS; **Identification:** identifiedBy: Norman F. Johnson; dateIdentified: 2012; **Event:** eventID: urn:lsid:biosci.ohio-state.edu:osuc_occurrences:SCAU__TEMP; samplingProtocol: none specified; eventDate: 2010-08-08/17; **Record Level:** modified: 2013-07-17T11:04:06Z; language: en; collectionID: urn:lsid:biocol.org:col:34252; collectionCode: Insects; basisOfRecord: PreservedSpecimen; source: http://hol.osu.edu/spmInfo.html?id=SCAU%20TEMP
- **Type status:** Other material. **Occurrence:** catalogNumber: SCAU 2010100357; recordedBy: Chen Hua-Yan; individualCount: 1; sex: female; lifeStage: adult; **Taxon:** taxonID: urn:lsid:biosci.ohio-state.edu:osuc_names:243848; scientificName: Oxyscelio florus; **Location:** country: China; stateProvince: Guangdong; locality: Nanling National Nature Reserve; locationRemarks: label transliteration: "Guangdong, Nanling, 2010.08.08-17, Chen Huayan"; [广东南岭, 2010.08.08-17, 陈华燕]; decimalLatitude: 24.9; decimalLongitude: 113; georeferenceProtocol: GPS; **Identification:** identifiedBy: Norman F. Johnson; dateIdentified: 2012; **Event:** eventID: urn:lsid:biosci.ohio-state.edu:osuc_occurrences:SCAU__2010100357; samplingProtocol: none specified; eventDate: 2010-08-08/17; **Record Level:** modified: 2013-07-17T11:03:25Z; language: en; collectionID: urn:lsid:biocol.org:col:34252; collectionCode: Insects; basisOfRecord: PreservedSpecimen; source: http://hol.osu.edu/spmInfo.html?id=SCAU%202010100357
- **Type status:** Other material. **Occurrence:** catalogNumber: SCAU 2010100168; recordedBy: Chen Hua-Yan; individualCount: 1; sex: female; lifeStage: adult; **Taxon:** taxonID: urn:lsid:biosci.ohio-state.edu:osuc_names:243848; scientificName: Oxyscelio florus; **Location:** country: China; stateProvince: Hunan; locality: Mangshan Nature Reserve; locationRemarks: label transliteration: "Hunan, Mangshan, 2010.VIII.13, Chen Huayan"; [湖南莽山自然保护区, 2010.VIII.13, 陈华燕]; decimalLatitude: 24.9333; decimalLongitude: 112.8167; georeferenceProtocol: GPS; georeferenceRemarks: GPS longitude minutes are incorrect (49 not 69); **Identification:** identifiedBy: Norman F. Johnson; dateIdentified: 2012; **Event:** eventID: urn:lsid:biosci.ohio-state.edu:osuc_occurrences:SCAU__2010100168; samplingProtocol: none specified; eventDate: 2010-08-13; **Record Level:** modified: 2013-07-17T11:03:24Z; language: en; collectionID: urn:lsid:biocol.org:col:34252; collectionCode: Insects; basisOfRecord: PreservedSpecimen; source: http://hol.osu.edu/spmInfo.html?id=SCAU%202010100168
- **Type status:** Other material. **Occurrence:** catalogNumber: SCAU 2010100166; recordedBy: Chen Hua-Yan; individualCount: 1; sex: female; lifeStage: adult; **Taxon:** taxonID: urn:lsid:biosci.ohio-state.edu:osuc_names:243848; scientificName: Oxyscelio florus; **Location:** country: China; stateProvince: Hunan; locality: Mangshan Nature Reserve; locationRemarks: label transliteration: "Hunan, Mangshan, 2010.VIII.13, Chen Huayan"; [湖南莽山自然保护区, 2010.VIII.13, 陈华燕]; decimalLatitude: 24.9333; decimalLongitude: 112.8167; georeferenceProtocol: GPS; georeferenceRemarks: GPS longitude minutes are incorrect (49 not 69); **Identification:** identifiedBy: Norman F. Johnson; dateIdentified: 2012; **Event:** eventID: urn:lsid:biosci.ohio-state.edu:osuc_occurrences:SCAU__2010100166; samplingProtocol: none specified; eventDate: 2010-08-13; **Record Level:** modified: 2013-07-17T11:03:24Z; language: en; collectionID: urn:lsid:biocol.org:col:34252; collectionCode: Insects; basisOfRecord: PreservedSpecimen; source: http://hol.osu.edu/spmInfo.html?id=SCAU%202010100166
- **Type status:** Other material. **Occurrence:** catalogNumber: SCAU 2010100157; recordedBy: Chen Hua-Yan; individualCount: 1; sex: female; lifeStage: adult; **Taxon:** taxonID: urn:lsid:biosci.ohio-state.edu:osuc_names:243848; scientificName: Oxyscelio florus; **Location:** country: China; stateProvince: Hunan; locality: Mangshan Nature Reserve; locationRemarks: label transliteration: "Hunan, Mangshan, 2010.VIII.13, Chen Huayan"; [湖南莽山自然保护区, 2010.VIII.13, 陈华燕]; decimalLatitude: 24.9333; decimalLongitude: 112.8167; georeferenceProtocol: GPS; georeferenceRemarks: GPS longitude minutes are incorrect (49 not 69); **Identification:** identifiedBy: Norman F. Johnson; dateIdentified: 2012; **Event:** eventID: urn:lsid:biosci.ohio-state.edu:osuc_occurrences:SCAU__2010100157; samplingProtocol: none specified; eventDate: 2010-08-13; **Record Level:** modified: 2013-07-17T11:03:23Z; language: en; collectionID: urn:lsid:biocol.org:col:34252; collectionCode: Insects; basisOfRecord: PreservedSpecimen; source: http://hol.osu.edu/spmInfo.html?id=SCAU%202010100157
- **Type status:** Other material. **Occurrence:** catalogNumber: SCAU 2010100152; recordedBy: Chen Hua-Yan; individualCount: 1; sex: female; lifeStage: adult; **Taxon:** taxonID: urn:lsid:biosci.ohio-state.edu:osuc_names:243848; scientificName: Oxyscelio florus; **Location:** country: China; stateProvince: Hunan; locality: Mangshan Nature Reserve; locationRemarks: label transliteration: "Hunan, Mangshan, 2010.VIII.13, Chen Huayan"; [湖南莽山自然保护区, 2010.VIII.13, 陈华燕]; decimalLatitude: 24.9333; decimalLongitude: 112.8167; georeferenceProtocol: GPS; georeferenceRemarks: GPS longitude minutes are incorrect (49 not 69); **Identification:** identifiedBy: Norman F. Johnson; dateIdentified: 2012; **Event:** eventID: urn:lsid:biosci.ohio-state.edu:osuc_occurrences:SCAU__2010100152; samplingProtocol: none specified; eventDate: 2010-08-13; **Record Level:** modified: 2013-07-17T11:03:23Z; language: en; collectionID: urn:lsid:biocol.org:col:34252; collectionCode: Insects; basisOfRecord: PreservedSpecimen; source: http://hol.osu.edu/spmInfo.html?id=SCAU%202010100152
- **Type status:** Other material. **Occurrence:** catalogNumber: SCAU 2011000081; recordedBy: Chen Hua-Yan; individualCount: 1; sex: female; lifeStage: adult; **Taxon:** taxonID: urn:lsid:biosci.ohio-state.edu:osuc_names:243848; scientificName: Oxyscelio florus; **Location:** country: China; stateProvince: Zhejiang; locality: Tianmushan National Nature Reserve, Mt. Xianrending; verbatimLocality: 1200 m; locationRemarks: label transliteration: "Zhejiang, Tianmushan, Xianrending, 2011.07.25-29, Chen Huayan"; [浙江天目山仙人顶 1200 m, 30°20.56'N 119°26.03'E, 2011.07.25-29, sweeping, 陈华燕]; verbatimCoordinates: 30°20.56'N 119°26.03'E; decimalLatitude: 30.3427; decimalLongitude: 119.4338; georeferenceProtocol: label; **Identification:** identifiedBy: Norman F. Johnson; dateIdentified: 2012; **Event:** eventID: urn:lsid:biosci.ohio-state.edu:osuc_occurrences:SCAU__2011000081; samplingProtocol: sweeping; eventDate: 2011-07-25/29; **Record Level:** modified: 2013-07-17T11:03:32Z; language: en; collectionID: urn:lsid:biocol.org:col:34252; collectionCode: Insects; basisOfRecord: PreservedSpecimen; source: http://hol.osu.edu/spmInfo.html?id=SCAU%202011000081
- **Type status:** Other material. **Occurrence:** catalogNumber: SCAU 2011000071; recordedBy: Chen Hua-Yan; individualCount: 1; sex: female; lifeStage: adult; **Taxon:** taxonID: urn:lsid:biosci.ohio-state.edu:osuc_names:243848; scientificName: Oxyscelio florus; **Location:** country: China; stateProvince: Zhejiang; locality: Tianmushan National Nature Reserve, Mt. Xianrending; verbatimLocality: 1200 m; locationRemarks: label transliteration: "Zhejiang, Tianmushan, Xianrending, 2011.07.25-29, Chen Huayan"; [浙江天目山仙人顶 1200 m, 30°20.56'N 119°26.03'E, 2011.07.25-29, sweeping, 陈华燕]; verbatimCoordinates: 30°20.56'N 119°26.03'E; decimalLatitude: 30.3427; decimalLongitude: 119.4338; georeferenceProtocol: label; **Identification:** identifiedBy: Norman F. Johnson; dateIdentified: 2012; **Event:** eventID: urn:lsid:biosci.ohio-state.edu:osuc_occurrences:SCAU__2011000071; samplingProtocol: sweeping; eventDate: 2011-07-25/29; **Record Level:** modified: 2013-07-17T11:03:30Z; language: en; collectionID: urn:lsid:biocol.org:col:34252; collectionCode: Insects; basisOfRecord: PreservedSpecimen; source: http://hol.osu.edu/spmInfo.html?id=SCAU%202011000071
- **Type status:** Other material. **Occurrence:** catalogNumber: SCAU 2011000052; recordedBy: Jin Cheng-Yuan; individualCount: 1; sex: female; lifeStage: adult; **Taxon:** taxonID: urn:lsid:biosci.ohio-state.edu:osuc_names:243848; scientificName: Oxyscelio florus; **Location:** country: China; stateProvince: Zhejiang; locality: Tianmushan National Nature Reserve, Mt. Xianrending; verbatimLocality: 1200 m; locationRemarks: label transliteration: "Zhejiang, Tianmushan, Xianrending, 2011.07.25-29, Jin Chengyuan"; [浙江天目山仙人顶 1200 m, 30°20.56'N 119°26.03'E, 2011.07.25-29, sweeping, 金程远]; verbatimCoordinates: 30°20.56'N 119°26.03'E; decimalLatitude: 30.3427; decimalLongitude: 119.4338; georeferenceProtocol: label; **Identification:** identifiedBy: Norman F. Johnson; dateIdentified: 2012; **Event:** eventID: urn:lsid:biosci.ohio-state.edu:osuc_occurrences:SCAU__2011000052; samplingProtocol: sweeping; eventDate: 2011-07-25/29; **Record Level:** modified: 2013-07-17T11:03:29Z; language: en; collectionID: urn:lsid:biocol.org:col:34252; collectionCode: Insects; basisOfRecord: PreservedSpecimen; source: http://hol.osu.edu/spmInfo.html?id=SCAU%202011000052

#### Distribution

*Oxyscelio florus* was originally described from Japan, and the recent redescription of Burks et al. 2013 had only Japanese specimens. The new data greatly extend the distribution of the species: it occurs widely in China from Hebei in the north, to Zhejiang, and to southern Hunan and Guangdong in the south. Link to dynamic distribution map：http://hol.osu.edu/map-large.html?id=243848

### Oxyscelio granorum

Burks, 2013

http://lsid.tdwg.org/urn:lsid:biosci.ohio-state.edu:osuc_concepts:275482

urn:lsid:zoobank.org:act:A531A356-5AB2-4314-A0A8-727D9BAC04F3

http://species-id.net/wiki/Oxyscelio_granorum

Oxyscelio granorum Burks et al. 2013: 15, 24, 144. Original description, keyed, placed in *cuculli* species group.

#### Materials

- **Type status:** Other material. **Occurrence:** catalogNumber: SCAU 2011000474; recordedBy: Shi Min; individualCount: 1; sex: male; lifeStage: adult; **Taxon:** taxonID: urn:lsid:biosci.ohio-state.edu:osuc_names:275482; scientificName: Oxyscelio granorum; **Location:** country: China; stateProvince: Shaanxi; locality: Liping National Forest Park; locationRemarks: label transliteration: "Shaanxi, Liping Forest Park, 2004.07.22, Shi Min"; [陕西黎坪森林公园, 2004.07.22, 时敏]; decimalLatitude: 32.8428; decimalLongitude: 106.6047; georeferenceProtocol: Google Earth; georeferenceRemarks: derived from https://plus.google.com/109967535777522360617/about?gl=us&hl=en; **Identification:** identifiedBy: Norman F. Johnson; dateIdentified: 2012; **Event:** eventID: urn:lsid:biosci.ohio-state.edu:osuc_occurrences:SCAU__2011000474; samplingProtocol: none specified; eventDate: 2004-07-22; **Record Level:** modified: 2013-07-17T11:03:54Z; language: en; collectionID: urn:lsid:biocol.org:col:34252; collectionCode: Insects; basisOfRecord: PreservedSpecimen; source: http://hol.osu.edu/spmInfo.html?id=SCAU%202011000474
- **Type status:** Other material. **Occurrence:** catalogNumber: SCAU 2011000078; recordedBy: Chen Hua-Yan; individualCount: 1; sex: female; lifeStage: adult; **Taxon:** taxonID: urn:lsid:biosci.ohio-state.edu:osuc_names:275482; scientificName: Oxyscelio granorum; **Location:** country: China; stateProvince: Zhejiang; locality: Tianmushan National Nature Reserve, Mt. Xianrending; verbatimLocality: 1200 m; locationRemarks: label transliteration: "Zhejiang, Tianmushan, Xianrending, 2011.07.25-29, Chen Huayan"; [浙江天目山仙人顶 1200 m, 30°20.56'N 119°26.03'E, 2011.07.25-29, sweeping, 陈华燕]; verbatimCoordinates: 30°20.56'N 119°26.03'E; decimalLatitude: 30.3427; decimalLongitude: 119.4338; georeferenceProtocol: label; **Identification:** identifiedBy: Norman F. Johnson; dateIdentified: 2012; **Event:** eventID: urn:lsid:biosci.ohio-state.edu:osuc_occurrences:SCAU__2011000078; samplingProtocol: sweeping; eventDate: 2011-07-25/29; **Record Level:** modified: 2013-07-17T11:03:32Z; language: en; collectionID: urn:lsid:biocol.org:col:34252; collectionCode: Insects; basisOfRecord: PreservedSpecimen; source: http://hol.osu.edu/spmInfo.html?id=SCAU%202011000078

#### Distribution

This species was originally described from specimens distributed widely in the tropics of southeast Asia. The Chinese specimens extend the distribution far to the north, to Shaanxi in northwest China and Zhejiang in eastern China. Link to dynamic distribution map: http://hol.osu.edu/map-large.html?id=275482

### Oxyscelio intermedietas

Burks, 2013

http://lsid.tdwg.org/urn:lsid:biosci.ohio-state.edu:osuc_concepts:275481

urn:lsid:zoobank.org:act:5902A8F2-DCD3-4FEE-9C14-AC346622EA2A

http://species-id.net/wiki/Oxyscelio_intermedietas

Oxyscelio intermedietas Burks et al. 2013: 15, 24, 153. Original description, placed in *cuculli* species group.

#### Materials

- **Type status:** Other material. **Occurrence:** catalogNumber: SCAU 200905895; recordedBy: Ma Juan-Juan; individualCount: 1; sex: male; lifeStage: adult; **Taxon:** taxonID: urn:lsid:biosci.ohio-state.edu:osuc_names:275481; scientificName: Oxyscelio intermedietas; **Location:** country: China; stateProvince: Guangdong; locality: Nanling National Nature Reserve, Guangdong Prov., China; locationRemarks: label transliteration: "Guangdong, Nanling, 2004.07.09-18, Ma Juanjuan"; [广东南岭, 2004.07.09-18, 马娟娟]; decimalLatitude: 24.9; decimalLongitude: 113; georeferenceProtocol: GPS; **Identification:** identifiedBy: Norman F. Johnson; dateIdentified: 2012; **Event:** eventID: urn:lsid:biosci.ohio-state.edu:osuc_occurrences:SCAU__200905895; samplingProtocol: none specified; eventDate: 2004-07-09/18; **Record Level:** modified: 2013-07-17T11:03:17Z; language: en; collectionID: urn:lsid:biocol.org:col:34252; collectionCode: Insects; basisOfRecord: PreservedSpecimen; source: http://hol.osu.edu/spmInfo.html?id=SCAU%20200905895
- **Type status:** Other material. **Occurrence:** catalogNumber: SCAU 2011000605; recordedBy: Shi Min; individualCount: 1; sex: female; lifeStage: adult; **Taxon:** taxonID: urn:lsid:biosci.ohio-state.edu:osuc_names:275481; scientificName: Oxyscelio intermedietas; **Location:** country: China; stateProvince: Shaanxi; locality: Zibaishan National Forest Park; verbatimLocality: 1632 m; locationRemarks: label transliteration: "Shaanxi, Ziboshan, 2004.08.03, Shi Min"; [陕西紫柏山, 1632m, 2004.08.03, 时敏]; decimalLatitude: 33.7089; decimalLongitude: 106.7525; georeferenceProtocol: Google Earth; **Identification:** identifiedBy: Norman F. Johnson; dateIdentified: 2012; **Event:** eventID: urn:lsid:biosci.ohio-state.edu:osuc_occurrences:SCAU__2011000605; samplingProtocol: none specified; eventDate: 2004-08-03; **Record Level:** modified: 2013-07-17T11:03:57Z; language: en; collectionID: urn:lsid:biocol.org:col:34252; collectionCode: Insects; basisOfRecord: PreservedSpecimen; source: http://hol.osu.edu/spmInfo.html?id=SCAU%202011000605
- **Type status:** Other material. **Occurrence:** catalogNumber: SCAU 2011000603; recordedBy: Shi Min; individualCount: 1; sex: male; lifeStage: adult; **Taxon:** taxonID: urn:lsid:biosci.ohio-state.edu:osuc_names:275481; scientificName: Oxyscelio intermedietas; **Location:** country: China; stateProvince: Shaanxi; locality: Zibaishan National Forest Park; verbatimLocality: 1632 m; locationRemarks: label transliteration: "Shaanxi, Ziboshan, 2004.08.03, Shi Min"; [陕西紫柏山, 1632m, 2004.08.03, 时敏]; decimalLatitude: 33.7089; decimalLongitude: 106.7525; georeferenceProtocol: Google Earth; **Identification:** identifiedBy: Norman F. Johnson; dateIdentified: 2012; **Event:** eventID: urn:lsid:biosci.ohio-state.edu:osuc_occurrences:SCAU__2011000603; samplingProtocol: none specified; eventDate: 2004-08-03; **Record Level:** modified: 2013-07-17T11:03:57Z; language: en; collectionID: urn:lsid:biocol.org:col:34252; collectionCode: Insects; basisOfRecord: PreservedSpecimen; source: http://hol.osu.edu/spmInfo.html?id=SCAU%202011000603
- **Type status:** Other material. **Occurrence:** catalogNumber: SCAU 2011000647; recordedBy: Shi Min; individualCount: 1; sex: male; lifeStage: adult; **Taxon:** taxonID: urn:lsid:biosci.ohio-state.edu:osuc_names:275481; scientificName: Oxyscelio intermedietas; **Location:** country: China; stateProvince: Zhejiang; locality: Gutianshan National Nature Reserve; locationRemarks: label transliteration: "Zhejiang, Gutianshan, 2005.07.03, Shi Min"; [浙江古田山, 2005.07.03, 时敏]; decimalLatitude: 29.2636; decimalLongitude: 118.1339; georeferenceProtocol: GEOnet; **Identification:** identifiedBy: Norman F. Johnson; dateIdentified: 2012; **Event:** eventID: urn:lsid:biosci.ohio-state.edu:osuc_occurrences:SCAU__2011000647; samplingProtocol: none specified; eventDate: 2005-07-03; **Record Level:** modified: 2013-07-17T11:04:02Z; language: en; collectionID: urn:lsid:biocol.org:col:34252; collectionCode: Insects; basisOfRecord: PreservedSpecimen; source: http://hol.osu.edu/spmInfo.html?id=SCAU%202011000647
- **Type status:** Other material. **Occurrence:** catalogNumber: SCAU 2011000381; recordedBy: Liu Jing-Xian; individualCount: 1; sex: male; lifeStage: adult; **Taxon:** taxonID: urn:lsid:biosci.ohio-state.edu:osuc_names:275481; scientificName: Oxyscelio intermedietas; **Location:** country: China; stateProvince: Hebei; locality: Xiaowutaishan National Natural Reserve; locationRemarks: label transliteration: "Hebei, Xiaowutaishan, 2005.08.23, Liu Jingxian"; [河北小五台山, 2005.08.23, 刘经贤]; decimalLatitude: 39.8697; decimalLongitude: 115.0653; georeferenceProtocol: Google Earth; georeferenceRemarks: derived from http://www.xiaowutai.cn/_d272489469.htm; **Identification:** identifiedBy: Norman F. Johnson; dateIdentified: 2012; **Event:** eventID: urn:lsid:biosci.ohio-state.edu:osuc_occurrences:SCAU__2011000381; samplingProtocol: none specified; eventDate: 2005-08-23; **Record Level:** modified: 2013-07-17T11:03:49Z; language: en; collectionID: urn:lsid:biocol.org:col:34252; collectionCode: Insects; basisOfRecord: PreservedSpecimen; source: http://hol.osu.edu/spmInfo.html?id=SCAU%202011000381
- **Type status:** Other material. **Occurrence:** catalogNumber: SCAU 2011000382; recordedBy: Liu Jing-Xian; individualCount: 1; sex: male; lifeStage: adult; **Taxon:** taxonID: urn:lsid:biosci.ohio-state.edu:osuc_names:275481; scientificName: Oxyscelio intermedietas; **Location:** country: China; stateProvince: Hebei; locality: Xiaowutaishan National Natural Reserve; locationRemarks: label transliteration: "Hebei, Xiaowutaishan, 2005.08.23, Liu Jingxian"; [河北小五台山, 2005.08.23, 刘经贤]; decimalLatitude: 39.8697; decimalLongitude: 115.0653; georeferenceProtocol: Google Earth; georeferenceRemarks: derived from http://www.xiaowutai.cn/_d272489469.htm; **Identification:** identifiedBy: Norman F. Johnson; dateIdentified: 2012; **Event:** eventID: urn:lsid:biosci.ohio-state.edu:osuc_occurrences:SCAU__2011000382; samplingProtocol: none specified; eventDate: 2005-08-23; **Record Level:** modified: 2013-07-17T11:03:49Z; language: en; collectionID: urn:lsid:biocol.org:col:34252; collectionCode: Insects; basisOfRecord: PreservedSpecimen; source: http://hol.osu.edu/spmInfo.html?id=SCAU%202011000382
- **Type status:** Other material. **Occurrence:** catalogNumber: SCAU 200906114; recordedBy: Liu Jing-Xian, et al.; individualCount: 1; sex: male; lifeStage: adult; **Taxon:** taxonID: urn:lsid:biosci.ohio-state.edu:osuc_names:275481; scientificName: Oxyscelio intermedietas; **Location:** country: China; stateProvince: Hainan; locality: Bawangling National Nature Reserve; locationRemarks: label transliteration: "Hainan, Bawangling, 2006.07.07-11, Liu Jingxian et al."; [海南霸王岭, 2006.07.07-11, 刘经贤等]; decimalLatitude: 19.1167; decimalLongitude: 109.05; georeferenceProtocol: GPS; **Identification:** identifiedBy: Norman F. Johnson; dateIdentified: 2012; **Event:** eventID: urn:lsid:biosci.ohio-state.edu:osuc_occurrences:SCAU__200906114; samplingProtocol: none specified; eventDate: 2006-07-07/11; **Record Level:** modified: 2013-07-17T11:03:18Z; language: en; collectionID: urn:lsid:biocol.org:col:34252; collectionCode: Insects; basisOfRecord: PreservedSpecimen; source: http://hol.osu.edu/spmInfo.html?id=SCAU%20200906114
- **Type status:** Other material. **Occurrence:** catalogNumber: SCAU 200706682; recordedBy: Liu Jing-Xian; individualCount: 1; sex: female; lifeStage: adult; **Taxon:** taxonID: urn:lsid:biosci.ohio-state.edu:osuc_names:275481; scientificName: Oxyscelio intermedietas; **Location:** country: China; stateProvince: Hainan; locality: Mt Diaoluoshan; locationRemarks: label transliteration: "Hainan, Diaoluoshan, 2007.5.28-30, Liu Jingxian"; [海南吊罗山 2007.5.28-30，刘经贤]; decimalLatitude: 18.65; decimalLongitude: 109.8833; georeferenceProtocol: GPS; **Identification:** identifiedBy: Norman F. Johnson; dateIdentified: 2012; **Event:** eventID: urn:lsid:biosci.ohio-state.edu:osuc_occurrences:SCAU__200706682; samplingProtocol: none specified; eventDate: 2007-05-28/30; **Record Level:** modified: 2013-07-17T11:03:15Z; language: en; collectionID: urn:lsid:biocol.org:col:34252; collectionCode: Insects; basisOfRecord: PreservedSpecimen; source: http://hol.osu.edu/spmInfo.html?id=SCAU%20200706682
- **Type status:** Other material. **Occurrence:** catalogNumber: SCAU 200706657; recordedBy: Liu Jing-Xian; individualCount: 1; sex: female; lifeStage: adult; **Taxon:** taxonID: urn:lsid:biosci.ohio-state.edu:osuc_names:275481; scientificName: Oxyscelio intermedietas; **Location:** country: China; stateProvince: Hainan; locality: Mt Diaoluoshan; locationRemarks: label transliteration: "Hainan, Diaoluoshan,2007.5.28-30, Liu Jingxian"; [海南吊罗山 2007.5.28-30，刘经贤]; decimalLatitude: 18.65; decimalLongitude: 109.8833; georeferenceProtocol: GPS; **Identification:** identifiedBy: Norman F. Johnson; dateIdentified: 2012; **Event:** eventID: urn:lsid:biosci.ohio-state.edu:osuc_occurrences:SCAU__200706657; samplingProtocol: none specified; eventDate: 2007-05-28/30; **Record Level:** modified: 2013-07-17T11:03:15Z; language: en; collectionID: urn:lsid:biocol.org:col:34252; collectionCode: Insects; basisOfRecord: PreservedSpecimen; source: http://hol.osu.edu/spmInfo.html?id=SCAU%20200706657
- **Type status:** Other material. **Occurrence:** catalogNumber: SCAU 2011000719; recordedBy: Fan Wu-Qing; individualCount: 1; sex: female; lifeStage: adult; **Taxon:** taxonID: urn:lsid:biosci.ohio-state.edu:osuc_names:275481; scientificName: Oxyscelio intermedietas; **Location:** country: China; stateProvince: Zhejiang; locality: Mt Fengyang; locationRemarks: label transliteration: "Zhejiang, Fengyangshan, 2008.07.30, Liu Jingxian"; [浙江凤阳山, 1000m, 2008.07.30, 刘经贤]; decimalLatitude: 27.9333; decimalLongitude: 119.2; georeferenceProtocol: GPS; **Identification:** identifiedBy: Norman F. Johnson; dateIdentified: 2012; **Event:** eventID: urn:lsid:biosci.ohio-state.edu:osuc_occurrences:SCAU__2011000719; samplingProtocol: none specified; eventDate: 2008-07-30; **Record Level:** modified: 2013-07-17T11:04:04Z; language: en; collectionID: urn:lsid:biocol.org:col:34252; collectionCode: Insects; basisOfRecord: PreservedSpecimen; source: http://hol.osu.edu/spmInfo.html?id=SCAU%202011000719
- **Type status:** Other material. **Occurrence:** catalogNumber: SCAU 2011000253; recordedBy: Chen Hua-Yan; individualCount: 1; sex: female; lifeStage: adult; **Taxon:** taxonID: urn:lsid:biosci.ohio-state.edu:osuc_names:275481; scientificName: Oxyscelio intermedietas; **Location:** country: China; stateProvince: Hainan; locality: Mt Yinggeling; locationRemarks: label transliteration: "Hainan, Yinggeshan, 2008.11.16 Chen Huayan"; [海南鹦哥岭, 2008.11.16, 陈华燕]; decimalLatitude: 18.8167; decimalLongitude: 109.1833; georeferenceProtocol: GPS; **Identification:** identifiedBy: Norman F. Johnson; dateIdentified: 2012; **Event:** eventID: urn:lsid:biosci.ohio-state.edu:osuc_occurrences:SCAU__2011000253; samplingProtocol: none specified; eventDate: 2008-11-16; **Record Level:** modified: 2013-07-17T11:03:47Z; language: en; collectionID: urn:lsid:biocol.org:col:34252; collectionCode: Insects; basisOfRecord: PreservedSpecimen; source: http://hol.osu.edu/spmInfo.html?id=SCAU%202011000253
- **Type status:** Other material. **Occurrence:** catalogNumber: SCAU 2011000255; recordedBy: Chen Hua-Yan; individualCount: 1; sex: female; lifeStage: adult; **Taxon:** taxonID: urn:lsid:biosci.ohio-state.edu:osuc_names:275481; scientificName: Oxyscelio intermedietas; **Location:** country: China; stateProvince: Hainan; locality: Mt Yinggeling; locationRemarks: label transliteration: "Hainan, Yinggeshan, 2008.11.16, Chen Huayan"; [海南鹦哥岭, 2008.11.16, 陈华燕]; decimalLatitude: 18.8167; decimalLongitude: 109.1833; georeferenceProtocol: GPS; **Identification:** identifiedBy: Norman F. Johnson; dateIdentified: 2012; **Event:** eventID: urn:lsid:biosci.ohio-state.edu:osuc_occurrences:SCAU__2011000255; samplingProtocol: none specified; eventDate: 2008-11-16; **Record Level:** modified: 2013-07-17T11:03:47Z; language: en; collectionID: urn:lsid:biocol.org:col:34252; collectionCode: Insects; basisOfRecord: PreservedSpecimen; source: http://hol.osu.edu/spmInfo.html?id=SCAU%202011000255
- **Type status:** Other material. **Occurrence:** catalogNumber: SCAU 2011000039; recordedBy: Wang Man-Man; individualCount: 1; sex: female; lifeStage: adult; **Taxon:** taxonID: urn:lsid:biosci.ohio-state.edu:osuc_names:275481; scientificName: Oxyscelio intermedietas; **Location:** country: China; stateProvince: Hainan; locality: Bawangling National Nature Reserve; locationRemarks: label transliteration: "Hainan, Bawangling, 2008.11.26, Wang Manman"; [海南霸王岭, 2008.11.26, 王漫漫]; decimalLatitude: 19.1167; decimalLongitude: 109.05; georeferenceProtocol: GPS; **Identification:** identifiedBy: Norman F. Johnson; dateIdentified: 2012; **Event:** eventID: urn:lsid:biosci.ohio-state.edu:osuc_occurrences:SCAU__2011000039; samplingProtocol: none specified; eventDate: 2008-11-26; **Record Level:** modified: 2013-07-17T11:03:28Z; language: en; collectionID: urn:lsid:biocol.org:col:34252; collectionCode: Insects; basisOfRecord: PreservedSpecimen; source: http://hol.osu.edu/spmInfo.html?id=SCAU%202011000039
- **Type status:** Other material. **Occurrence:** catalogNumber: SCAU 2011000040; recordedBy: Wang Man-Man; individualCount: 1; sex: female; lifeStage: adult; **Taxon:** taxonID: urn:lsid:biosci.ohio-state.edu:osuc_names:275481; scientificName: Oxyscelio intermedietas; **Location:** country: China; stateProvince: Hainan; locality: Bawangling National Nature Reserve; locationRemarks: label transliteration: "Hainan, Bawangling, 2008.11.26, Wang Manman"; [海南霸王岭, 2008.11.26, 王漫漫]; decimalLatitude: 19.1167; decimalLongitude: 109.05; georeferenceProtocol: GPS; **Identification:** identifiedBy: Norman F. Johnson; dateIdentified: 2012; **Event:** eventID: urn:lsid:biosci.ohio-state.edu:osuc_occurrences:SCAU__2011000040; samplingProtocol: none specified; eventDate: 2008-11-26; **Record Level:** modified: 2013-07-17T11:03:28Z; language: en; collectionID: urn:lsid:biocol.org:col:34252; collectionCode: Insects; basisOfRecord: PreservedSpecimen; source: http://hol.osu.edu/spmInfo.html?id=SCAU%202011000040
- **Type status:** Other material. **Occurrence:** catalogNumber: SCAU 200906479; recordedBy: Wang Man-Man; individualCount: 1; sex: male; lifeStage: adult; **Taxon:** taxonID: urn:lsid:biosci.ohio-state.edu:osuc_names:275481; scientificName: Oxyscelio intermedietas; **Location:** country: China; stateProvince: Yunnan; county: Dehong Dai and Jingpo; locality: Tongbiguan Pass; locationRemarks: label transliteration: "Yunnan, Yingjiang, Tongbiguan, 2009.05.16, Wang Manman "; [云南盈江铜壁关, 2009.05.16, 王漫漫]; decimalLatitude: 24.25; decimalLongitude: 97.8167; georeferenceProtocol: GPS; **Identification:** identifiedBy: Norman F. Johnson; dateIdentified: 2012; **Event:** eventID: urn:lsid:biosci.ohio-state.edu:osuc_occurrences:SCAU__200906479; samplingProtocol: none specified; eventDate: 2008-11-16; **Record Level:** modified: 2013-07-17T11:03:20Z; language: en; collectionID: urn:lsid:biocol.org:col:34252; collectionCode: Insects; basisOfRecord: PreservedSpecimen; source: http://hol.osu.edu/spmInfo.html?id=SCAU%20200906479
- **Type status:** Other material. **Occurrence:** catalogNumber: SCAU 200906461; recordedBy: Wang Man-Man; individualCount: 1; sex: female; lifeStage: adult; **Taxon:** taxonID: urn:lsid:biosci.ohio-state.edu:osuc_names:275481; scientificName: Oxyscelio intermedietas; **Location:** country: China; stateProvince: Yunnan; county: Dehong Dai and Jingpo; locality: Tongbiguan Pass; locationRemarks: label transliteration: "Yunnan, Yingjiang, Tongbiguan, 2009.05.16, Wang Manman "; [云南盈江铜壁关, 2009.05.16, 王漫漫]; decimalLatitude: 24.25; decimalLongitude: 97.8167; georeferenceProtocol: GPS; **Identification:** identifiedBy: Norman F. Johnson; dateIdentified: 2012; **Event:** eventID: urn:lsid:biosci.ohio-state.edu:osuc_occurrences:SCAU__200906461; samplingProtocol: none specified; eventDate: 2008-11-16; **Record Level:** modified: 2013-07-17T11:03:19Z; language: en; collectionID: urn:lsid:biocol.org:col:34252; collectionCode: Insects; basisOfRecord: PreservedSpecimen; source: http://hol.osu.edu/spmInfo.html?id=SCAU%20200906461
- **Type status:** Other material. **Occurrence:** catalogNumber: SCAU 2011000115; recordedBy: Chen Hua-Yan; individualCount: 1; sex: female; lifeStage: adult; **Taxon:** taxonID: urn:lsid:biosci.ohio-state.edu:osuc_names:275481; scientificName: Oxyscelio intermedietas; **Location:** country: China; stateProvince: Zhejiang; locality: Tianmushan National Nature Reserve, Mt. Xianrending; verbatimElevation: 1200 m; locationRemarks: label transliteration: "Zhejiang, Tianmushan, Xianrending, 2011.07.25-29, Chen Huayan"; [浙江天目山仙人顶 1200 m, 30°20.56'N 119°26.03'E, 2011.07.25-29, YPT, 陈华燕]; verbatimCoordinates: 30°20.56'N 119°26.03'E; decimalLatitude: 30.3427; decimalLongitude: 119.4338; georeferenceProtocol: label; **Identification:** identifiedBy: Norman F. Johnson; dateIdentified: 2012; **Event:** eventID: urn:lsid:biosci.ohio-state.edu:osuc_occurrences:SCAU__2011000115; samplingProtocol: yellow pan trap; eventDate: 2011-07-25/29; **Record Level:** modified: 2013-07-17T11:03:41Z; language: en; collectionID: urn:lsid:biocol.org:col:34252; collectionCode: Insects; basisOfRecord: PreservedSpecimen; source: http://hol.osu.edu/spmInfo.html?id=SCAU%202011000115
- **Type status:** Other material. **Occurrence:** catalogNumber: SCAU 2011000112; recordedBy: Chen Hua-Yan; individualCount: 1; sex: female; lifeStage: adult; **Taxon:** taxonID: urn:lsid:biosci.ohio-state.edu:osuc_names:275481; scientificName: Oxyscelio intermedietas; **Location:** country: China; stateProvince: Zhejiang; locality: Tianmushan National Nature Reserve, Mt. Xianrending; verbatimElevation: 1200 m; locationRemarks: label transliteration: "Zhejiang, Tianmushan, Xianrending, 2011.07.25-29, Chen Huayan"; [浙江天目山仙人顶 1200 m, 30°20.56'N 119°26.03'E, 2011.07.25-29, YPT, 陈华燕]; verbatimCoordinates: 30°20.56'N 119°26.03'E; decimalLatitude: 30.3427; decimalLongitude: 119.4338; georeferenceProtocol: label; **Identification:** identifiedBy: Norman F. Johnson; dateIdentified: 2012; **Event:** eventID: urn:lsid:biosci.ohio-state.edu:osuc_occurrences:SCAU__2011000112; samplingProtocol: yellow pan trap; eventDate: 2011-07-25/29; **Record Level:** modified: 2013-07-17T11:03:41Z; language: en; collectionID: urn:lsid:biocol.org:col:34252; collectionCode: Insects; basisOfRecord: PreservedSpecimen; source: http://hol.osu.edu/spmInfo.html?id=SCAU%202011000112
- **Type status:** Other material. **Occurrence:** catalogNumber: SCAU 2011000111; recordedBy: Chen Hua-Yan; individualCount: 1; sex: female; lifeStage: adult; **Taxon:** taxonID: urn:lsid:biosci.ohio-state.edu:osuc_names:275481; scientificName: Oxyscelio intermedietas; **Location:** country: China; stateProvince: Zhejiang; locality: Tianmushan National Nature Reserve, Mt. Xianrending; verbatimElevation: 1200 m; locationRemarks: label transliteration: "Zhejiang, Tianmushan, Xianrending, 2011.07.25-29, Chen Huayan"; [浙江天目山仙人顶 1200 m, 30°20.56'N 119°26.03'E, 2011.07.25-29, YPT, 陈华燕]; verbatimCoordinates: 30°20.56'N 119°26.03'E; decimalLatitude: 30.3427; decimalLongitude: 119.4338; georeferenceProtocol: label; **Identification:** identifiedBy: Norman F. Johnson; dateIdentified: 2012; **Event:** eventID: urn:lsid:biosci.ohio-state.edu:osuc_occurrences:SCAU__2011000111; samplingProtocol: yellow pan trap; eventDate: 2011-07-25/29; **Record Level:** modified: 2013-07-17T11:03:40Z; language: en; collectionID: urn:lsid:biocol.org:col:34252; collectionCode: Insects; basisOfRecord: PreservedSpecimen; source: http://hol.osu.edu/spmInfo.html?id=SCAU%202011000111
- **Type status:** Other material. **Occurrence:** catalogNumber: SCAU 2011000117; recordedBy: Chen Hua-Yan; individualCount: 1; sex: female; lifeStage: adult; **Taxon:** taxonID: urn:lsid:biosci.ohio-state.edu:osuc_names:275481; scientificName: Oxyscelio intermedietas; **Location:** country: China; stateProvince: Zhejiang; locality: Tianmushan National Nature Reserve, Mt. Xianrending; verbatimElevation: 1200 m; locationRemarks: label transliteration: "Zhejiang, Tianmushan, Xianrending, 2011.07.25-29, Chen Huayan"; [浙江天目山仙人顶 1200 m, 30°20.56'N 119°26.03'E, 2011.07.25-29, YPT, 陈华燕]; verbatimCoordinates: 30°20.56'N 119°26.03'E; decimalLatitude: 30.3427; decimalLongitude: 119.4338; georeferenceProtocol: label; **Identification:** identifiedBy: Norman F. Johnson; dateIdentified: 2012; **Event:** eventID: urn:lsid:biosci.ohio-state.edu:osuc_occurrences:SCAU__2011000117; samplingProtocol: yellow pan trap; eventDate: 2011-07-25/29; **Record Level:** modified: 2013-07-17T11:03:42Z; language: en; collectionID: urn:lsid:biocol.org:col:34252; collectionCode: Insects; basisOfRecord: PreservedSpecimen; source: http://hol.osu.edu/spmInfo.html?id=SCAU%202011000117
- **Type status:** Other material. **Occurrence:** catalogNumber: SCAU 2011000116; recordedBy: Chen Hua-Yan; individualCount: 1; sex: female; lifeStage: adult; **Taxon:** taxonID: urn:lsid:biosci.ohio-state.edu:osuc_names:275481; scientificName: Oxyscelio intermedietas; **Location:** country: China; stateProvince: Zhejiang; locality: Tianmushan National Nature Reserve, Mt. Xianrending; verbatimElevation: 1200 m; locationRemarks: label transliteration: "Zhejiang, Tianmushan, Xianrending, 2011.07.25-29, Chen Huayan"; [浙江天目山仙人顶 1200 m, 30°20.56'N 119°26.03'E, 2011.07.25-29, YPT, 陈华燕]; verbatimCoordinates: 30°20.56'N 119°26.03'E; decimalLatitude: 30.3427; decimalLongitude: 119.4338; georeferenceProtocol: label; **Identification:** identifiedBy: Norman F. Johnson; dateIdentified: 2012; **Event:** eventID: urn:lsid:biosci.ohio-state.edu:osuc_occurrences:SCAU__2011000116; samplingProtocol: yellow pan trap; eventDate: 2011-07-25/29; **Record Level:** modified: 2013-07-17T11:03:41Z; language: en; collectionID: urn:lsid:biocol.org:col:34252; collectionCode: Insects; basisOfRecord: PreservedSpecimen; source: http://hol.osu.edu/spmInfo.html?id=SCAU%202011000116

#### Distribution

Originally this species was known to be widespread from Nepal to southeast Asia. These first records from China document it in Shaanxi, Hebei, Zhejiang, Yunnan, Guangdong, and Hainan. Link to dynamic distribution map: http://hol.osu.edu/map-large.html?id=275481

### Oxyscelio jugi

Burks, 2013

http://lsid.tdwg.org/urn:lsid:biosci.ohio-state.edu:osuc_concepts:275571

urn:lsid:zoobank.org:act:2D8F217A-8522-4F42-B41C-9038D798B6E7

http://species-id.net/wiki/Oxyscelio_jugi

Oxyscelio jugi Burks et al. 2013: 15, 24, 25, 158. Original description, keyed, placed in *crebritas* species group.

#### Materials

- **Type status:** Other material. **Occurrence:** catalogNumber: SCAU 200906128; recordedBy: Liu Jing-Xian, et al.; individualCount: 1; sex: female; lifeStage: adult; **Taxon:** taxonID: urn:lsid:biosci.ohio-state.edu:osuc_names:275571; scientificName: Oxyscelio jugi; **Location:** country: China; stateProvince: Hainan; locality: Bawangling National Nature Reserve; locationRemarks: label transliteration: "Hainan, Bawangling, 2006.07.07-11, Liu Jingxian et al."; [海南霸王岭, 2006.07.07-11, 刘经贤等]; decimalLatitude: 19.1167; decimalLongitude: 109.05; georeferenceProtocol: GPS; **Identification:** identifiedBy: Norman F. Johnson; dateIdentified: 2012; **Event:** eventID: urn:lsid:biosci.ohio-state.edu:osuc_occurrences:SCAU__200906128; samplingProtocol: none specified; eventDate: 2006-07-07/11; **Record Level:** modified: 2013-07-17T11:03:18Z; language: en; collectionID: urn:lsid:biocol.org:col:34252; collectionCode: Insects; basisOfRecord: PreservedSpecimen; source: http://hol.osu.edu/spmInfo.html?id=SCAU%20200906128
- **Type status:** Other material. **Occurrence:** catalogNumber: SCAU 200906473; recordedBy: Wang Man-Man; individualCount: 1; sex: male; lifeStage: adult; **Taxon:** taxonID: urn:lsid:biosci.ohio-state.edu:osuc_names:275571; scientificName: Oxyscelio jugi; **Location:** country: China; stateProvince: Yunnan; county: Dehong Dai and Jingpo; locality: Tongbiguan Pass; locationRemarks: label transliteration: "Yunnan, Yingjiang, Tongbiguan, 2009.05.16, Wang Manman "; [云南盈江铜壁关, 2009.05.16, 王漫漫]; decimalLatitude: 24.25; decimalLongitude: 97.8167; georeferenceProtocol: GPS; **Identification:** identifiedBy: Norman F. Johnson; dateIdentified: 2012; **Event:** eventID: urn:lsid:biosci.ohio-state.edu:osuc_occurrences:SCAU__200906473; samplingProtocol: none specified; eventDate: 2009-05-16; **Record Level:** modified: 2013-07-17T11:03:19Z; language: en; collectionID: urn:lsid:biocol.org:col:34252; collectionCode: Insects; basisOfRecord: PreservedSpecimen; source: http://hol.osu.edu/spmInfo.html?id=SCAU%20200906473
- **Type status:** Other material. **Occurrence:** catalogNumber: SCAU 200906477; recordedBy: Wang Man-Man; individualCount: 1; sex: male; lifeStage: adult; **Taxon:** taxonID: urn:lsid:biosci.ohio-state.edu:osuc_names:275571; scientificName: Oxyscelio jugi; **Location:** country: China; stateProvince: Yunnan; county: Dehong Dai and Jingpo; locality: Tongbiguan Pass; locationRemarks: label transliteration: "Yunnan, Yingjiang, Tongbiguan, 2009.05.16, Wang Manman "; [云南盈江铜壁关, 2009.05.16, 王漫漫]; decimalLatitude: 24.25; decimalLongitude: 97.8167; georeferenceProtocol: GPS; **Identification:** identifiedBy: Norman F. Johnson; dateIdentified: 2012; **Event:** eventID: urn:lsid:biosci.ohio-state.edu:osuc_occurrences:SCAU__200906477; samplingProtocol: none specified; eventDate: 2009-05-16; **Record Level:** modified: 2013-07-17T11:03:20Z; language: en; collectionID: urn:lsid:biocol.org:col:34252; collectionCode: Insects; basisOfRecord: PreservedSpecimen; source: http://hol.osu.edu/spmInfo.html?id=SCAU%20200906477

#### Distribution

*Oxyscelio jugi* is found widely in southeast Asia. Not surprisingly, its range also extends into the nearby regions of Yunnan and Hainan. Link to dynamic distribution map: http://hol.osu.edu/map-large.html?id=275571

### Oxyscelio kramatos

Burks, 2013

http://lsid.tdwg.org/urn:lsid:biosci.ohio-state.edu:osuc_concepts:305703

urn:lsid:zoobank.org:act:F06E950E-9DE5-4330-A32A-F6C42BE982C6

http://species-id.net/wiki/Oxyscelio_kramatos

Oxyscelio kramatos Burks et al. 2013: 15, 24, 25, 163. Original description, keyed, placed in *cuculli* species group.

#### Materials

- **Type status:** Other material. **Occurrence:** catalogNumber: SCAU 200905882; recordedBy: Ma Juan-Juan; individualCount: 1; sex: female; lifeStage: adult; **Taxon:** taxonID: urn:lsid:biosci.ohio-state.edu:osuc_names:305703; scientificName: Oxyscelio kramatos; **Location:** country: China; stateProvince: Guangdong; locality: Nanling National Nature Reserve; locationRemarks: label transliteration: "Guangdong, Nanling, 2004.07.09-18, Ma Juanjuan"; [广东南岭 2004.07.09-18, 马娟娟]; decimalLatitude: 24.9; decimalLongitude: 113; georeferenceProtocol: GPS; **Identification:** identifiedBy: Norman F. Johnson; dateIdentified: 2012; **Event:** eventID: urn:lsid:biosci.ohio-state.edu:osuc_occurrences:SCAU__200905882; samplingProtocol: none specified; eventDate: 2004-07-09/18; **Record Level:** modified: 2013-07-17T11:03:17Z; language: en; collectionID: urn:lsid:biocol.org:col:34252; collectionCode: Insects; basisOfRecord: PreservedSpecimen; source: http://hol.osu.edu/spmInfo.html?id=SCAU%20200905882
- **Type status:** Other material. **Occurrence:** catalogNumber: SCAU 2011000514; recordedBy: Zhang Hong-Ying; individualCount: 1; sex: female; lifeStage: adult; **Taxon:** taxonID: urn:lsid:biosci.ohio-state.edu:osuc_names:305703; scientificName: Oxyscelio kramatos; **Location:** country: China; stateProvince: Zhejiang; locality: Mt Qingliangfeng; locationRemarks: label transliteration: "Zhejiang, Qingliangfeng, 2005.08.10, Zhang Hongying"; [浙江清凉峰 2005.08.10 张红英]; decimalLatitude: 30.0703; decimalLongitude: 118.8944; georeferenceProtocol: Google Earth; georeferenceRemarks: GPS coords. adjusted to place within Zhejiang Prov.; **Identification:** identifiedBy: Norman F. Johnson; dateIdentified: 2012; **Event:** eventID: urn:lsid:biosci.ohio-state.edu:osuc_occurrences:SCAU__2011000514; samplingProtocol: none specified; eventDate: 2005-08-10; **Record Level:** modified: 2013-07-17T11:03:55Z; language: en; collectionID: urn:lsid:biocol.org:col:34252; collectionCode: Insects; basisOfRecord: PreservedSpecimen; source: http://hol.osu.edu/spmInfo.html?id=SCAU%202011000514
- **Type status:** Other material. **Occurrence:** catalogNumber: SCAU 2011000732; recordedBy: Shi Min; individualCount: 1; sex: female; lifeStage: adult; **Taxon:** taxonID: urn:lsid:biosci.ohio-state.edu:osuc_names:305703; scientificName: Oxyscelio kramatos; **Location:** country: China; stateProvince: Zhejiang; locality: Mt Qingliangfeng; locationRemarks: label transliteration: "Zhejiang, Qingliangfeng, 2005.08.10, Shi Min"; [浙江清凉峰 2005.08.10 时敏]; decimalLatitude: 30.0703; decimalLongitude: 118.8944; georeferenceProtocol: Google Earth; georeferenceRemarks: GPS coords. adjusted to place within Zhejiang Prov.; **Identification:** identifiedBy: Norman F. Johnson; dateIdentified: 2012; **Event:** eventID: urn:lsid:biosci.ohio-state.edu:osuc_occurrences:SCAU__2011000732; samplingProtocol: none specified; eventDate: 2005-08-10; **Record Level:** modified: 2013-07-17T11:04:04Z; language: en; collectionID: urn:lsid:biocol.org:col:34252; collectionCode: Insects; basisOfRecord: PreservedSpecimen; source: http://hol.osu.edu/spmInfo.html?id=SCAU%202011000732
- **Type status:** Other material. **Occurrence:** catalogNumber: SCAU 200603485; recordedBy: Zhang Hong-Ying; individualCount: 1; sex: female; lifeStage: adult; **Taxon:** taxonID: urn:lsid:biosci.ohio-state.edu:osuc_names:305703; scientificName: Oxyscelio kramatos; **Location:** country: China; stateProvince: Zhejiang; county: Hangzhou; locality: Mt Qingliangfeng; locationRemarks: label transliteration: "Zhejiang, Qingliangfeng, 2005.8.12, Zhang Hongying"; [浙江临安清凉峰, 2005.8.12, 张红英]; decimalLatitude: 30.0703; decimalLongitude: 118.8944; georeferenceProtocol: Google Earth; georeferenceRemarks: GPS coords. adjusted to place within Lin'an City; **Identification:** identifiedBy: Norman F. Johnson; dateIdentified: 2012; **Event:** eventID: urn:lsid:biosci.ohio-state.edu:osuc_occurrences:SCAU__200603485; samplingProtocol: none specified; eventDate: 2005-08-12; **Record Level:** modified: 2013-07-17T11:03:10Z; language: en; collectionID: urn:lsid:biocol.org:col:34252; collectionCode: Insects; basisOfRecord: PreservedSpecimen; source: http://hol.osu.edu/spmInfo.html?id=SCAU%20200603485
- **Type status:** Other material. **Occurrence:** catalogNumber: SCAU 2011000721; recordedBy: Fan Wu-Qing; individualCount: 1; sex: female; lifeStage: adult; **Taxon:** taxonID: urn:lsid:biosci.ohio-state.edu:osuc_names:305703; scientificName: Oxyscelio kramatos; **Location:** country: China; stateProvince: Zhejiang; locality: Mt Fengyang; locationRemarks: label transliteration: "Zhejiang, Fengyangshan, 2008.07.30, Fan Wuqing"; [浙江凤阳山, 1000m, 2008.07.30, 范武青]; decimalLatitude: 27.9333; decimalLongitude: 119.2; georeferenceProtocol: GPS; **Identification:** identifiedBy: Norman F. Johnson; dateIdentified: 2012; **Event:** eventID: urn:lsid:biosci.ohio-state.edu:osuc_occurrences:SCAU__2011000721; samplingProtocol: none specified; eventDate: 2008-07-30; **Record Level:** modified: 2013-07-17T11:04:04Z; language: en; collectionID: urn:lsid:biocol.org:col:34252; collectionCode: Insects; basisOfRecord: PreservedSpecimen; source: http://hol.osu.edu/spmInfo.html?id=SCAU%202011000721
- **Type status:** Other material. **Occurrence:** catalogNumber: SCAU 2011000761; recordedBy: Fan Wu-Qing; individualCount: 1; sex: female; lifeStage: adult; **Taxon:** taxonID: urn:lsid:biosci.ohio-state.edu:osuc_names:305703; scientificName: Oxyscelio kramatos; **Location:** country: China; stateProvince: Zhejiang; locality: Mt Fengyang; locationRemarks: label transliteration: "Zhejiang, Fengyangshan,... , Fan Wuqing"; [浙江凤阳山, 1000m, 2008.07.30, 范武青]; decimalLatitude: 27.9333; decimalLongitude: 119.2; georeferenceProtocol: GPS; **Identification:** identifiedBy: Norman F. Johnson; dateIdentified: 2012; **Event:** eventID: urn:lsid:biosci.ohio-state.edu:osuc_occurrences:SCAU__2011000761; samplingProtocol: none specified; eventDate: 2008-07-30; **Record Level:** modified: 2013-07-17T11:04:05Z; language: en; collectionID: urn:lsid:biocol.org:col:34252; collectionCode: Insects; basisOfRecord: PreservedSpecimen; source: http://hol.osu.edu/spmInfo.html?id=SCAU%202011000761
- **Type status:** Other material. **Occurrence:** catalogNumber: SCAU 2011000263; recordedBy: Tang Pu; individualCount: 1; sex: female; lifeStage: adult; **Taxon:** taxonID: urn:lsid:biosci.ohio-state.edu:osuc_names:305703; scientificName: Oxyscelio kramatos; **Location:** country: Taiwan; stateProvince: Kaohsiung; locality: Shoushan National Nature Park; verbatimElevation: 120 m; locationRemarks: label transliteration: "Taiwan, Gaoxiong, Shou shan, Tang Pu"; [台湾高雄寿山, 1200m, 22.65°N 120'27°E, 2011.05.29, sweeping, 唐璞]; verbatimCoordinates: 22.65°N 120.27°E; decimalLatitude: 22.65; decimalLongitude: 120.27; georeferenceProtocol: label; **Identification:** identifiedBy: Norman F. Johnson; dateIdentified: 2012; **Event:** eventID: urn:lsid:biosci.ohio-state.edu:osuc_occurrences:SCAU__2011000263; samplingProtocol: sweeping; eventDate: 2011-05-29; **Record Level:** modified: 2013-07-17T11:03:47Z; language: en; collectionID: urn:lsid:biocol.org:col:34252; collectionCode: Insects; basisOfRecord: PreservedSpecimen; source: http://hol.osu.edu/spmInfo.html?id=SCAU%202011000263
- **Type status:** Other material. **Occurrence:** catalogNumber: SCAU 2011000158; recordedBy: Xu Zai-Fu, et al.; individualCount: 1; sex: female; lifeStage: adult; **Taxon:** taxonID: urn:lsid:biosci.ohio-state.edu:osuc_names:305703; scientificName: Oxyscelio kramatos; **Location:** country: China; stateProvince: Guangxi Zhuang; locality: Longshan Nature Reserve; verbatimElevation: 370 m; locationRemarks: label transliteration: "Guangxi, Longshan Nature Reserve, 2011.07.01-02, Xu Zaifu et al."; [广西龙山自然保护区 370 m, 23°24.727'N 108°31.918'E, 2011.07.01-02, YPT, 许再福等]; verbatimCoordinates: 23°24.727'N 108°31.918'E; decimalLatitude: 23.4121; decimalLongitude: 108.532; georeferenceProtocol: label; **Identification:** identifiedBy: Norman F. Johnson; dateIdentified: 2012; **Event:** eventID: urn:lsid:biosci.ohio-state.edu:osuc_occurrences:SCAU__2011000158; samplingProtocol: yellow pan trap; eventDate: 2011-07-01/02; **Record Level:** modified: 2013-07-17T11:03:46Z; language: en; collectionID: urn:lsid:biocol.org:col:34252; collectionCode: Insects; basisOfRecord: PreservedSpecimen; source: http://hol.osu.edu/spmInfo.html?id=SCAU%202011000158
- **Type status:** Other material. **Occurrence:** catalogNumber: SCAU 2011000084; recordedBy: Chen Hua-Yan; individualCount: 1; sex: male; lifeStage: adult; **Taxon:** taxonID: urn:lsid:biosci.ohio-state.edu:osuc_names:305703; scientificName: Oxyscelio kramatos; **Location:** country: China; stateProvince: Zhejiang; locality: Tianmushan National Nature Reserve, Mt. Xianrending; verbatimElevation: 1200 m; locationRemarks: label transliteration: "Zhejiang, Tianmushan, Xianrending, 2011.07.25-29, Chen Huayan"; [浙江天目山仙人顶 1200 m, 30°20.56'N 119°26.03'E, 2011.07.25-29, sweeping, 陈华燕]; verbatimCoordinates: 30°20.56'N 119°26.03'E; decimalLatitude: 30.3427; decimalLongitude: 119.4338; georeferenceProtocol: label; **Identification:** identifiedBy: Norman F. Johnson; dateIdentified: 2012; **Event:** eventID: urn:lsid:biosci.ohio-state.edu:osuc_occurrences:SCAU__2011000084; samplingProtocol: sweeping; eventDate: 2011-07-25/29; **Record Level:** modified: 2013-07-17T11:03:33Z; language: en; collectionID: urn:lsid:biocol.org:col:34252; collectionCode: Insects; basisOfRecord: PreservedSpecimen; source: http://hol.osu.edu/spmInfo.html?id=SCAU%202011000084
- **Type status:** Other material. **Occurrence:** catalogNumber: SCAU 2011000077; recordedBy: Chen Hua-Yan; individualCount: 1; sex: female; lifeStage: adult; **Taxon:** taxonID: urn:lsid:biosci.ohio-state.edu:osuc_names:305703; scientificName: Oxyscelio kramatos; **Location:** country: China; stateProvince: Zhejiang; locality: Tianmushan National Nature Reserve, Mt. Xianrending; verbatimElevation: 1200 m; locationRemarks: label transliteration: "Zhejiang, Tianmushan, Xianrending, 2011.07.25-29, Chen Huayan"; [浙江天目山仙人顶 1200 m, 30°20.56'N 119°26.03'E, 2011.07.25-29, sweeping, 陈华燕]; verbatimCoordinates: 30°20.56'N 119°26.03'E; decimalLatitude: 30.3427; decimalLongitude: 119.4338; georeferenceProtocol: label; **Identification:** identifiedBy: Norman F. Johnson; dateIdentified: 2012; **Event:** eventID: urn:lsid:biosci.ohio-state.edu:osuc_occurrences:SCAU__2011000077; samplingProtocol: sweeping; eventDate: 2011-07-25/29; **Record Level:** modified: 2013-07-17T11:03:31Z; language: en; collectionID: urn:lsid:biocol.org:col:34252; collectionCode: Insects; basisOfRecord: PreservedSpecimen; source: http://hol.osu.edu/spmInfo.html?id=SCAU%202011000077
- **Type status:** Other material. **Occurrence:** catalogNumber: SCAU 2011000075; recordedBy: Chen Hua-Yan; individualCount: 1; sex: female; lifeStage: adult; **Taxon:** taxonID: urn:lsid:biosci.ohio-state.edu:osuc_names:305703; scientificName: Oxyscelio kramatos; **Location:** country: China; stateProvince: Zhejiang; locality: Tianmushan National Nature Reserve, Mt. Xianrending; verbatimElevation: 1200 m; locationRemarks: label transliteration: "Zhejiang,Tianmushan, Xianrending, 2011.07.25-29, Chen Huayan"; [浙江天目山仙人顶 1200 m, 30°20.56'N 119°26.03'E, 2011.07.25-29, sweeping, 陈华燕]; verbatimCoordinates: 30°20.56'N 119°26.03'E; decimalLatitude: 30.3427; decimalLongitude: 119.4338; georeferenceProtocol: label; **Identification:** identifiedBy: Norman F. Johnson; dateIdentified: 2012; **Event:** eventID: urn:lsid:biosci.ohio-state.edu:osuc_occurrences:SCAU__2011000075; samplingProtocol: sweeping; eventDate: 2011-07-25/29; **Record Level:** modified: 2013-07-17T11:03:31Z; language: en; collectionID: urn:lsid:biocol.org:col:34252; collectionCode: Insects; basisOfRecord: PreservedSpecimen; source: http://hol.osu.edu/spmInfo.html?id=SCAU%202011000075
- **Type status:** Other material. **Occurrence:** catalogNumber: SCAU 2011000074; recordedBy: Chen Hua-Yan; individualCount: 1; sex: female; lifeStage: adult; **Taxon:** taxonID: urn:lsid:biosci.ohio-state.edu:osuc_names:305703; scientificName: Oxyscelio kramatos; **Location:** country: China; stateProvince: Zhejiang; locality: Tianmushan National Nature Reserve, Mt. Xianrending; verbatimElevation: 1200 m; locationRemarks: label transliteration: "Zhejiang, Tianmushan, Xianrending, 2011.07.25-29, Chen Huayan"; [浙江天目山仙人顶 1200 m, 30°20.56'N 119°26.03'E, 2011.07.25-29, sweeping, 陈华燕]; verbatimCoordinates: 30°20.56'N 119°26.03'E; decimalLatitude: 30.3427; decimalLongitude: 119.4338; georeferenceProtocol: label; **Identification:** identifiedBy: Norman F. Johnson; dateIdentified: 2012; **Event:** eventID: urn:lsid:biosci.ohio-state.edu:osuc_occurrences:SCAU__2011000074; samplingProtocol: sweeping; eventDate: 2011-07-25/29; **Record Level:** modified: 2013-07-17T11:03:31Z; language: en; collectionID: urn:lsid:biocol.org:col:34252; collectionCode: Insects; basisOfRecord: PreservedSpecimen; source: http://hol.osu.edu/spmInfo.html?id=SCAU%202011000074
- **Type status:** Other material. **Occurrence:** catalogNumber: SCAU 2011000094; recordedBy: Chen Hua-Yan; individualCount: 1; sex: female; lifeStage: adult; **Taxon:** taxonID: urn:lsid:biosci.ohio-state.edu:osuc_names:305703; scientificName: Oxyscelio kramatos; **Location:** country: China; stateProvince: Zhejiang; locality: Tianmushan National Nature Reserve, Mt. Xianrending; verbatimElevation: 1200 m; locationRemarks: label transliteration: "Zhejiang, Tianmushan, Xianrending, 2011.07.25-29, Chen Huayan"; [浙江天目山仙人顶 1200 m, 30°20.56'N 119°26.03'E, 2011.07.25-29, sweeping, 陈华燕]; verbatimCoordinates: 30°20.56'N 119°26.03'E; decimalLatitude: 30.3427; decimalLongitude: 119.4338; georeferenceProtocol: label; **Identification:** identifiedBy: Norman F. Johnson; dateIdentified: 2012; **Event:** eventID: urn:lsid:biosci.ohio-state.edu:osuc_occurrences:SCAU__2011000094; samplingProtocol: sweeping; eventDate: 2011-07-25/29; **Record Level:** modified: 2013-07-17T11:03:37Z; language: en; collectionID: urn:lsid:biocol.org:col:34252; collectionCode: Insects; basisOfRecord: PreservedSpecimen; source: http://hol.osu.edu/spmInfo.html?id=SCAU%202011000094
- **Type status:** Other material. **Occurrence:** catalogNumber: SCAU 2011000091; recordedBy: Chen Hua-Yan; individualCount: 1; sex: female; lifeStage: adult; **Taxon:** taxonID: urn:lsid:biosci.ohio-state.edu:osuc_names:305703; scientificName: Oxyscelio kramatos; **Location:** country: China; stateProvince: Zhejiang; locality: Tianmushan National Nature Reserve, Mt. Xianrending; verbatimElevation: 1200 m; locationRemarks: label transliteration: "Zhejiang, Tianmushan, Xianrending, 2011.07.25-29, Chen Huayan"; [浙江天目山仙人顶 1200 m, 30°20.56'N 119°26.03'E, 2011.07.25-29, sweeping, 陈华燕]; verbatimCoordinates: 30°20.56'N 119°26.03'E; decimalLatitude: 30.3427; decimalLongitude: 119.4338; georeferenceProtocol: label; **Identification:** identifiedBy: Norman F. Johnson; dateIdentified: 2012; **Event:** eventID: urn:lsid:biosci.ohio-state.edu:osuc_occurrences:SCAU__2011000091; samplingProtocol: sweeping; eventDate: 2011-07-25/29; **Record Level:** modified: 2013-07-17T11:03:35Z; language: en; collectionID: urn:lsid:biocol.org:col:34252; collectionCode: Insects; basisOfRecord: PreservedSpecimen; source: http://hol.osu.edu/spmInfo.html?id=SCAU%202011000091
- **Type status:** Other material. **Occurrence:** catalogNumber: SCAU 2011000070; recordedBy: Chen Hua-Yan; individualCount: 1; sex: female; lifeStage: adult; **Taxon:** taxonID: urn:lsid:biosci.ohio-state.edu:osuc_names:305703; scientificName: Oxyscelio kramatos; **Location:** country: China; stateProvince: Zhejiang; locality: Tianmushan National Nature Reserve, Mt. Xianrending; verbatimElevation: 1200 m; locationRemarks: label transliteration: "Zhejiang,Tianmushan, Xianrending, 2011.07.25-29, Chen Huayan"; [浙江天目山仙人顶 1200 m, 30°20.56'N 119°26.03'E, 2011.07.25-29, sweeping, 陈华燕]; verbatimCoordinates: 30°20.56'N 119°26.03'E; decimalLatitude: 30.3427; decimalLongitude: 119.4338; georeferenceProtocol: label; **Identification:** identifiedBy: Norman F. Johnson; dateIdentified: 2012; **Event:** eventID: urn:lsid:biosci.ohio-state.edu:osuc_occurrences:SCAU__2011000070; samplingProtocol: sweeping; eventDate: 2011-07-25/29; **Record Level:** modified: 2013-07-17T11:03:30Z; language: en; collectionID: urn:lsid:biocol.org:col:34252; collectionCode: Insects; basisOfRecord: PreservedSpecimen; source: http://hol.osu.edu/spmInfo.html?id=SCAU%202011000070
- **Type status:** Other material. **Occurrence:** catalogNumber: SCAU 2011000103; recordedBy: Jin Cheng-Yuan; individualCount: 1; sex: female; lifeStage: adult; **Taxon:** taxonID: urn:lsid:biosci.ohio-state.edu:osuc_names:305703; scientificName: Oxyscelio kramatos; **Location:** country: China; stateProvince: Zhejiang; locality: Tianmushan National Nature Reserve, Mt. Xianrending; verbatimElevation: 1200 m; locationRemarks: label transliteration: "Zhejiang, Tianmushan, Xianrending, 2011.07.25-29, Jin Chengyuan"; [浙江天目山仙人顶 1200 m, 30°20.56'N 119°26.03'E, 2011.07.25-29, YPT, 金程远]; verbatimCoordinates: 30°20.56'N 119°26.03'E; decimalLatitude: 30.3427; decimalLongitude: 119.4338; georeferenceProtocol: label; **Identification:** identifiedBy: Norman F. Johnson; dateIdentified: 2012; **Event:** eventID: urn:lsid:biosci.ohio-state.edu:osuc_occurrences:SCAU__2011000103; samplingProtocol: yellow pan trap; eventDate: 2011-07-25/29; **Record Level:** modified: 2013-07-17T11:03:39Z; language: en; collectionID: urn:lsid:biocol.org:col:34252; collectionCode: Insects; basisOfRecord: PreservedSpecimen; source: http://hol.osu.edu/spmInfo.html?id=SCAU%202011000103
- **Type status:** Other material. **Occurrence:** catalogNumber: SCAU 2011000099; recordedBy: Jin Cheng-Yuan; individualCount: 1; sex: female; lifeStage: adult; **Taxon:** taxonID: urn:lsid:biosci.ohio-state.edu:osuc_names:305703; scientificName: Oxyscelio kramatos; **Location:** country: China; stateProvince: Zhejiang; locality: Tianmushan National Nature Reserve, Mt. Xianrending; verbatimElevation: 1200 m; locationRemarks: label transliteration: "Zhejiang,Tianmushan, Xianrending, 2011.07.25-29, Jin Chengyuan"; [浙江天目山仙人顶 1200 m, 30°20.56'N 119°26.03'E, 2011.07.25-29, YPT, 金程远]; verbatimCoordinates: 30°20.56'N 119°26.03'E; decimalLatitude: 30.3427; decimalLongitude: 119.4338; georeferenceProtocol: label; **Identification:** identifiedBy: Norman F. Johnson; dateIdentified: 2012; **Event:** eventID: urn:lsid:biosci.ohio-state.edu:osuc_occurrences:SCAU__2011000099; samplingProtocol: yellow pan trap; eventDate: 2011-07-25/29; **Record Level:** modified: 2013-07-17T11:03:38Z; language: en; collectionID: urn:lsid:biocol.org:col:34252; collectionCode: Insects; basisOfRecord: PreservedSpecimen; source: http://hol.osu.edu/spmInfo.html?id=SCAU%202011000099
- **Type status:** Other material. **Occurrence:** catalogNumber: SCAU 2011000093; recordedBy: Chen Hua-Yan; individualCount: 1; sex: female; lifeStage: adult; **Taxon:** taxonID: urn:lsid:biosci.ohio-state.edu:osuc_names:305703; scientificName: Oxyscelio kramatos; **Location:** country: China; stateProvince: Zhejiang; locality: Tianmushan National Nature Reserve, Mt. Xianrending; verbatimElevation: 1200 m; locationRemarks: label transliteration: "Zhejiang,Tianmushan, Xianrending, 2011.07.25-29, Chen Huayan"; [浙江天目山仙人顶 1200 m, 30°20.56'N 119°26.03'E, 2011.07.25-29, sweeping, 陈华燕]; verbatimCoordinates: 30°20.56'N 119°26.03'E; decimalLatitude: 30.3427; decimalLongitude: 119.4338; georeferenceProtocol: label; **Identification:** identifiedBy: Norman F. Johnson; dateIdentified: 2012; **Event:** eventID: urn:lsid:biosci.ohio-state.edu:osuc_occurrences:SCAU__2011000093; samplingProtocol: sweeping; eventDate: 2011-07-25/29; **Record Level:** modified: 2013-07-17T11:03:36Z; language: en; collectionID: urn:lsid:biocol.org:col:34252; collectionCode: Insects; basisOfRecord: PreservedSpecimen; source: http://hol.osu.edu/spmInfo.html?id=SCAU%202011000093
- **Type status:** Other material. **Occurrence:** catalogNumber: SCAU 2011000089; recordedBy: Chen Hua-Yan; individualCount: 1; sex: female; lifeStage: adult; **Taxon:** taxonID: urn:lsid:biosci.ohio-state.edu:osuc_names:305703; scientificName: Oxyscelio kramatos; **Location:** country: China; stateProvince: Zhejiang; locality: Tianmushan National Nature Reserve, Mt. Xianrending; verbatimElevation: 1200 m; locationRemarks: label transliteration: "Zhejiang,Tianmushan, Xianrending, 2011.07.25-29, Chen Huayan"; [浙江天目山仙人顶 1200 m, 30°20.56'N 119°26.03'E, 2011.07.25-29, sweeping, 陈华燕]; verbatimCoordinates: 30°20.56'N 119°26.03'E; decimalLatitude: 30.3427; decimalLongitude: 119.4338; georeferenceProtocol: label; **Identification:** identifiedBy: Norman F. Johnson; dateIdentified: 2012; **Event:** eventID: urn:lsid:biosci.ohio-state.edu:osuc_occurrences:SCAU__2011000089; samplingProtocol: sweeping; eventDate: 2011-07-25/29; **Record Level:** modified: 2013-07-17T11:03:34Z; language: en; collectionID: urn:lsid:biocol.org:col:34252; collectionCode: Insects; basisOfRecord: PreservedSpecimen; source: http://hol.osu.edu/spmInfo.html?id=SCAU%202011000089

#### Distribution

Originally described from the island of Taiwan, this species is also found in Guangdong, Zhejiang, and Guangxi Provinces. Link to dynamic distribution map: http://hol.osu.edu/map-large.html?id=305703

### Oxyscelio longiventris

Burks, 2013

http://lsid.tdwg.org/urn:lsid:biosci.ohio-state.edu:osuc_concepts:275522

urn:lsid:zoobank.org:act:6BE6E08D-9977-4EC8-A7B5-50CDB92457FD

http://species-id.net/wiki/Oxyscelio_longiventris

Oxyscelio longiventris Burks et al. 2013: 15, 25, 177. Original description, keyed, placed in *crebritas* species group.

#### Materials

- **Type status:** Other material. **Occurrence:** catalogNumber: SCAU 2011000086; recordedBy: Chen Hua-Yan; individualCount: 1; sex: male; lifeStage: adult; **Taxon:** taxonID: urn:lsid:biosci.ohio-state.edu:osuc_names:275522; scientificName: Oxyscelio longiventris; **Location:** country: China; stateProvince: Zhejiang; locality: Tianmushan National Nature Reserve, Mt. Xianrending; verbatimElevation: 1200 m; locationRemarks: label transliteration: "Zhejiang,Tianmushan, Xianrending, 2011.07.25-29, Chen Huayan"; [浙江天目山仙人顶 1200 m, 30°20.56'N 119°26.03'E, 2011.07.25-29, sweeping, 陈华燕]; verbatimCoordinates: 30°20.56'N 119°26.03'E; decimalLatitude: 30.3427; decimalLongitude: 119.4338; georeferenceProtocol: label; **Identification:** identifiedBy: Norman F. Johnson; dateIdentified: 2012; **Event:** eventID: urn:lsid:biosci.ohio-state.edu:osuc_occurrences:SCAU__2011000086; samplingProtocol: sweeping; eventDate: 2011-07-25/29; **Record Level:** modified: 2013-07-17T11:03:34Z; language: en; collectionID: urn:lsid:biocol.org:col:34252; collectionCode: Insects; basisOfRecord: PreservedSpecimen; source: http://hol.osu.edu/spmInfo.html?id=SCAU%202011000086
- **Type status:** Other material. **Occurrence:** catalogNumber: SCAU 2011000085; recordedBy: Chen Hua-Yan; individualCount: 1; sex: male; lifeStage: adult; **Taxon:** taxonID: urn:lsid:biosci.ohio-state.edu:osuc_names:275522; scientificName: Oxyscelio longiventris; **Location:** country: China; stateProvince: Zhejiang; locality: Tianmushan National Nature Reserve, Mt. Xianrending; verbatimElevation: 1200 m; locationRemarks: label transliteration: "Zhejiang, Tianmushan, Xianrending, 2011.07.25-29, Chen Huayan"; [浙江天目山仙人顶 1200 m, 30°20.56'N 119°26.03'E, 2011.07.25-29, sweeping, 陈华燕]; verbatimCoordinates: 30°20.56'N 119°26.03'E; decimalLatitude: 30.3427; decimalLongitude: 119.4338; georeferenceProtocol: label; **Identification:** identifiedBy: Norman F. Johnson; dateIdentified: 2012; **Event:** eventID: urn:lsid:biosci.ohio-state.edu:osuc_occurrences:SCAU__2011000085; samplingProtocol: sweeping; eventDate: 2011-07-25/29; **Record Level:** modified: 2013-07-17T11:03:33Z; language: en; collectionID: urn:lsid:biocol.org:col:34252; collectionCode: Insects; basisOfRecord: PreservedSpecimen; source: http://hol.osu.edu/spmInfo.html?id=SCAU%202011000085
- **Type status:** Other material. **Occurrence:** catalogNumber: SCAU 2011000083; recordedBy: Chen Hua-Yan; individualCount: 1; sex: male; lifeStage: adult; **Taxon:** taxonID: urn:lsid:biosci.ohio-state.edu:osuc_names:275522; scientificName: Oxyscelio longiventris; **Location:** country: China; stateProvince: Zhejiang; locality: Tianmushan National Nature Reserve, Mt. Xianrending; verbatimElevation: 1200 m; locationRemarks: label transliteration: "Zhejiang, Tianmushan, Xianrending, 2011.07.25-29, Chen Huayan"; [浙江天目山仙人顶 1200 m, 30°20.56'N 119°26.03'E, 2011.07.25-29, sweeping, 陈华燕]; verbatimCoordinates: 30°20.56'N 119°26.03'E; decimalLatitude: 30.3427; decimalLongitude: 119.4338; georeferenceProtocol: label; **Identification:** identifiedBy: Norman F. Johnson; dateIdentified: 2012; **Event:** eventID: urn:lsid:biosci.ohio-state.edu:osuc_occurrences:SCAU__2011000083; samplingProtocol: sweeping; eventDate: 2011-07-25/29; **Record Level:** modified: 2013-07-17T11:03:33Z; language: en; collectionID: urn:lsid:biocol.org:col:34252; collectionCode: Insects; basisOfRecord: PreservedSpecimen; source: http://hol.osu.edu/spmInfo.html?id=SCAU%202011000083
- **Type status:** Other material. **Occurrence:** catalogNumber: SCAU 2011000053; recordedBy: Jin Cheng-Yuan; individualCount: 1; sex: male; lifeStage: adult; **Taxon:** taxonID: urn:lsid:biosci.ohio-state.edu:osuc_names:275522; scientificName: Oxyscelio longiventris; **Location:** country: China; stateProvince: Zhejiang; locality: Tianmushan National Nature Reserve, Mt. Xianrending; verbatimElevation: 1200 m; locationRemarks: label transliteration: "Zhejiang, Tianmushan, Xianrending, 2011.07.25-29, Jin Chengyuan"; [浙江天目山仙人顶 1200 m, 30°20.56'N 119°26.03'E, 2011.07.25-29, sweeping, 金程远]; verbatimCoordinates: 30°20.56'N 119°26.03'E; decimalLatitude: 30.3427; decimalLongitude: 119.4338; georeferenceProtocol: label; **Identification:** identifiedBy: Norman F. Johnson; dateIdentified: 2012; **Event:** eventID: urn:lsid:biosci.ohio-state.edu:osuc_occurrences:SCAU__2011000053; samplingProtocol: sweeping; eventDate: 2011-07-25/29; **Record Level:** modified: 2013-07-17T11:03:29Z; language: en; collectionID: urn:lsid:biocol.org:col:34252; collectionCode: Insects; basisOfRecord: PreservedSpecimen; source: http://hol.osu.edu/spmInfo.html?id=SCAU%202011000053
- **Type status:** Other material. **Occurrence:** catalogNumber: SCAU 2011000051; recordedBy: Jin Cheng-Yuan; individualCount: 1; sex: male; lifeStage: adult; **Taxon:** taxonID: urn:lsid:biosci.ohio-state.edu:osuc_names:275522; scientificName: Oxyscelio longiventris; **Location:** country: China; stateProvince: Zhejiang; locality: Tianmushan National Nature Reserve, Mt. Xianrending; verbatimElevation: 1200 m; locationRemarks: label transliteration: "Zhejiang, Tianmushan, Xianrending, 2011.07.25-29, Jin Chengyuan"; [浙江天目山仙人顶 1200 m, 30°20.56'N 119°26.03'E, 2011.07.25-29, sweeping, 金程远]; verbatimCoordinates: 30°20.56'N 119°26.03'E; decimalLatitude: 30.3427; decimalLongitude: 119.4338; georeferenceProtocol: label; **Identification:** identifiedBy: Norman F. Johnson; dateIdentified: 2012; **Event:** eventID: urn:lsid:biosci.ohio-state.edu:osuc_occurrences:SCAU__2011000051; samplingProtocol: sweeping; eventDate: 2011-07-25/29; **Record Level:** modified: 2013-07-17T11:03:29Z; language: en; collectionID: urn:lsid:biocol.org:col:34252; collectionCode: Insects; basisOfRecord: PreservedSpecimen; source: http://hol.osu.edu/spmInfo.html?id=SCAU%202011000051
- **Type status:** Other material. **Occurrence:** catalogNumber: SCAU 2011000050; recordedBy: Jin Cheng-Yuan; individualCount: 1; sex: male; lifeStage: adult; **Taxon:** taxonID: urn:lsid:biosci.ohio-state.edu:osuc_names:275522; scientificName: Oxyscelio longiventris; **Location:** country: China; stateProvince: Zhejiang; locality: Tianmushan National Nature Reserve, Mt. Xianrending; verbatimElevation: 1200 m; locationRemarks: label transliteration: "Zhejiang, Tianmushan, Xianrending, 2011.07.25-29, Jin Chengyuan"; [浙江天目山仙人顶 1200 m, 30°20.56'N 119°26.03'E, 2011.07.25-29, sweeping, 金程远]; verbatimCoordinates: 30°20.56'N 119°26.03'E; decimalLatitude: 30.3427; decimalLongitude: 119.4338; georeferenceProtocol: label; **Identification:** identifiedBy: Norman F. Johnson; dateIdentified: 2012; **Event:** eventID: urn:lsid:biosci.ohio-state.edu:osuc_occurrences:SCAU__2011000050; samplingProtocol: sweeping; eventDate: 2011-07-25/29; **Record Level:** modified: 2013-07-17T11:03:29Z; language: en; collectionID: urn:lsid:biocol.org:col:34252; collectionCode: Insects; basisOfRecord: PreservedSpecimen; source: http://hol.osu.edu/spmInfo.html?id=SCAU%202011000050
- **Type status:** Other material. **Occurrence:** catalogNumber: SCAU 2011000698; recordedBy: Jin Cheng-Yuan; individualCount: 1; sex: male; lifeStage: adult; **Taxon:** taxonID: urn:lsid:biosci.ohio-state.edu:osuc_names:275522; scientificName: Oxyscelio longiventris; **Location:** country: China; stateProvince: Zhejiang; locality: Tianmushan National Nature Reserve, Mt. Xianrending; verbatimElevation: 1200 m; locationRemarks: label transliteration: "Zhejiang, Tianmushan, Xianrending, 2011.07.25-29, Jin Chengyuan"; [浙江天目山仙人顶 1200 m, 30°20.56'N 119°26.03'E, 2011.07.25-29, sweeping, 金程远]; verbatimCoordinates: 30°20.56'N 119°26.03'E; decimalLatitude: 30.3427; decimalLongitude: 119.4338; georeferenceProtocol: label; **Identification:** identifiedBy: Norman F. Johnson; dateIdentified: 2012; **Event:** eventID: urn:lsid:biosci.ohio-state.edu:osuc_occurrences:SCAU__2011000698; samplingProtocol: sweeping; eventDate: 2011-07-25/29; **Record Level:** modified: 2013-07-17T11:04:03Z; language: en; collectionID: urn:lsid:biocol.org:col:34252; collectionCode: Insects; basisOfRecord: PreservedSpecimen; source: http://hol.osu.edu/spmInfo.html?id=SCAU%202011000698
- **Type status:** Other material. **Occurrence:** catalogNumber: SCAU 2011000092; recordedBy: Chen Hua-Yan; individualCount: 1; sex: male; lifeStage: adult; **Taxon:** taxonID: urn:lsid:biosci.ohio-state.edu:osuc_names:275522; scientificName: Oxyscelio longiventris; **Location:** country: China; stateProvince: Zhejiang; locality: Tianmushan National Nature Reserve, Mt. Xianrending; verbatimElevation: 1200 m; locationRemarks: label transliteration: "Zhejiang, Tianmushan, Xianrending, 2011.07.25-29, Chen Huayan"; [浙江天目山仙人顶 1200 m, 30°20.56'N 119°26.03'E, 2011.07.25-29, sweeping, 陈华燕]; verbatimCoordinates: 30°20.56'N 119°26.03'E; decimalLatitude: 30.3427; decimalLongitude: 119.4338; georeferenceProtocol: label; **Identification:** identifiedBy: Norman F. Johnson; dateIdentified: 2012; **Event:** eventID: urn:lsid:biosci.ohio-state.edu:osuc_occurrences:SCAU__2011000092; samplingProtocol: sweeping; eventDate: 2011-07-25/29; **Record Level:** modified: 2013-07-17T11:03:36Z; language: en; collectionID: urn:lsid:biocol.org:col:34252; collectionCode: Insects; basisOfRecord: PreservedSpecimen; source: http://hol.osu.edu/spmInfo.html?id=SCAU%202011000092

#### Distribution

*Oxyscelio longiventris* was described by Burks et al. 2013 on the basis of specimens from Laos and Thailand. Its distribution extends north to Zhejiang Province in eastern China. Link to dynamic distribution map: http://hol.osu.edu/map-large.html?id=275522

### Oxyscelio naraws

Kozlov & Lê, 2000

http://lsid.tdwg.org/urn:lsid:biosci.ohio-state.edu:osuc_concepts:179750

urn:lsid:zoobank.org:act:377E4C35-6CDC-4D4E-9C7B-537662DFB8F2

http://species-id.net/wiki/Oxyscelio_naraws

Oxyscelio naraws Lê 2000: 40, 326. Original description.

#### Materials

- **Type status:** Other material. **Occurrence:** catalogNumber: SCAU 2011000473; recordedBy: Shi Min; individualCount: 1; sex: male; lifeStage: adult; **Taxon:** taxonID: urn:lsid:biosci.ohio-state.edu:osuc_names:179750; scientificName: Oxyscelio naraws; **Location:** country: China; stateProvince: Zhejiang; locality: Mt Qingliangfeng; locationRemarks: label transliteration: "Zhejiang, Qingliangfeng, 2005.08.09, Shi Min"; [浙江清凉峰 2005.08.09 时敏]; decimalLatitude: 30.0703; decimalLongitude: 118.8944; georeferenceProtocol: Google Earth; georeferenceRemarks: GPS coords. adjusted to place within Zhejiang Prov.; **Identification:** identifiedBy: Norman F. Johnson; dateIdentified: 2012; **Event:** eventID: urn:lsid:biosci.ohio-state.edu:osuc_occurrences:SCAU__2011000473; samplingProtocol: none specified; eventDate: 2005-08-09; **Record Level:** modified: 2013-07-17T11:03:54Z; language: en; collectionID: urn:lsid:biocol.org:col:34252; collectionCode: Insects; basisOfRecord: PreservedSpecimen; source: http://hol.osu.edu/spmInfo.html?id=SCAU%202011000473
- **Type status:** Other material. **Occurrence:** catalogNumber: SCAU 2011000471; recordedBy: Shi Min; individualCount: 1; sex: female; lifeStage: adult; **Taxon:** taxonID: urn:lsid:biosci.ohio-state.edu:osuc_names:179750; scientificName: Oxyscelio naraws; **Location:** country: China; stateProvince: Zhejiang; locality: Mt Qingliangfeng; locationRemarks: label transliteration: "Zhejiang, Qingliangfeng, 2005.08.09, Shi Min"; [浙江清凉峰 2005.08.09 时敏]; decimalLatitude: 30.0703; decimalLongitude: 118.8944; georeferenceProtocol: Google Earth; georeferenceRemarks: GPS coords. adjusted to place within Zhejiang Prov.; **Identification:** identifiedBy: Norman F. Johnson; dateIdentified: 2012; **Event:** eventID: urn:lsid:biosci.ohio-state.edu:osuc_occurrences:SCAU__2011000471; samplingProtocol: none specified; eventDate: 2005-08-09; **Record Level:** modified: 2013-07-17T11:03:54Z; language: en; collectionID: urn:lsid:biocol.org:col:34252; collectionCode: Insects; basisOfRecord: PreservedSpecimen; source: http://hol.osu.edu/spmInfo.html?id=SCAU%202011000471
- **Type status:** Other material. **Occurrence:** catalogNumber: SCAU 2011000470; recordedBy: Shi Min; individualCount: 1; sex: female; lifeStage: adult; **Taxon:** taxonID: urn:lsid:biosci.ohio-state.edu:osuc_names:179750; scientificName: Oxyscelio naraws; **Location:** country: China; stateProvince: Zhejiang; locality: Mt Qingliangfeng; locationRemarks: label transliteration: "Zhejiang, Qingliangfeng, 2005.08.09, Shi Min"; [浙江清凉峰 2005.08.09 时敏]; decimalLatitude: 30.0703; decimalLongitude: 118.8944; georeferenceProtocol: Google Earth; georeferenceRemarks: GPS coords. adjusted to place within Zhejiang Prov.; **Identification:** identifiedBy: Norman F. Johnson; dateIdentified: 2012; **Event:** eventID: urn:lsid:biosci.ohio-state.edu:osuc_occurrences:SCAU__2011000470; samplingProtocol: none specified; eventDate: 2005-08-09; **Record Level:** modified: 2013-07-17T11:03:54Z; language: en; collectionID: urn:lsid:biocol.org:col:34252; collectionCode: Insects; basisOfRecord: PreservedSpecimen; source: http://hol.osu.edu/spmInfo.html?id=SCAU%202011000470
- **Type status:** Other material. **Occurrence:** catalogNumber: SCAU 2011000362; recordedBy: Chen Hua-Yan; individualCount: 1; sex: female; lifeStage: adult; **Taxon:** taxonID: urn:lsid:biosci.ohio-state.edu:osuc_names:179750; scientificName: Oxyscelio naraws; **Location:** country: China; stateProvince: Guangdong; locality: Nanling National Nature Reserve; locationRemarks: label transliteration: "Guangdong, Nanling, 2010.08.08, Chen Huayan"; [广东南岭国家自然保护区, 2010.08.08, YPT, 陈华燕]; decimalLatitude: 24.9; decimalLongitude: 113; georeferenceProtocol: GPS; **Identification:** identifiedBy: Norman F. Johnson; dateIdentified: 2012; **Event:** eventID: urn:lsid:biosci.ohio-state.edu:osuc_occurrences:SCAU__2011000362; samplingProtocol: yellow pan trap; eventDate: 2005-08-08; **Record Level:** modified: 2013-07-17T11:03:48Z; language: en; collectionID: urn:lsid:biocol.org:col:34252; collectionCode: Insects; basisOfRecord: PreservedSpecimen; source: http://hol.osu.edu/spmInfo.html?id=SCAU%202011000362
- **Type status:** Other material. **Occurrence:** catalogNumber: SCAU 2010100358; recordedBy: Chen Hua-Yan; individualCount: 1; sex: female; lifeStage: adult; **Taxon:** taxonID: urn:lsid:biosci.ohio-state.edu:osuc_names:179750; scientificName: Oxyscelio naraws; **Location:** country: China; stateProvince: Guangdong; locality: Nanling National Nature Reserve; locationRemarks: label transliteration: "Guangdong, Nanling, 2010.08.08-17, Chen Huayan"; [广东南岭, 2010.08.08-17, 陈华燕]; decimalLatitude: 24.9; decimalLongitude: 113; georeferenceProtocol: GPS; **Identification:** identifiedBy: Norman F. Johnson; dateIdentified: 2012; **Event:** eventID: urn:lsid:biosci.ohio-state.edu:osuc_occurrences:SCAU__2010100358; samplingProtocol: none specified; eventDate: 2005-08-08-17; **Record Level:** modified: 2013-07-17T11:03:25Z; language: en; collectionID: urn:lsid:biocol.org:col:34252; collectionCode: Insects; basisOfRecord: PreservedSpecimen; source: http://hol.osu.edu/spmInfo.html?id=SCAU%202010100358
- **Type status:** Other material. **Occurrence:** catalogNumber: SCAU 2011000073; recordedBy: Chen Hua-Yan; individualCount: 1; sex: male; lifeStage: adult; **Taxon:** taxonID: urn:lsid:biosci.ohio-state.edu:osuc_names:179750; scientificName: Oxyscelio naraws; **Location:** country: China; stateProvince: Zhejiang; locality: Tianmushan National Nature Reserve, Mt. Xianrending; verbatimElevation: 1200 m; locationRemarks: label transliteration: "Zhejiang, Tianmushan, Xianrending, 2011.07.25-29, Chen Huayan"; [浙江天目山仙人顶 1200 m, 30°20.56'N 119°26.03'E, 2011.07.25-29, sweeping, 陈华燕]; verbatimCoordinates: 30°20.56'N 119°26.03'E; decimalLatitude: 30.3427; decimalLongitude: 119.4338; georeferenceProtocol: label; **Identification:** identifiedBy: Norman F. Johnson; dateIdentified: 2012; **Event:** eventID: urn:lsid:biosci.ohio-state.edu:osuc_occurrences:SCAU__2011000073; samplingProtocol: sweeping; eventDate: 2011-07-25/29; **Record Level:** modified: 2013-07-17T11:03:30Z; language: en; collectionID: urn:lsid:biocol.org:col:34252; collectionCode: Insects; basisOfRecord: PreservedSpecimen; source: http://hol.osu.edu/spmInfo.html?id=SCAU%202011000073
- **Type status:** Other material. **Occurrence:** catalogNumber: SCAU 2011000109; recordedBy: Jin Cheng-Yuan; individualCount: 1; sex: male; lifeStage: adult; **Taxon:** taxonID: urn:lsid:biosci.ohio-state.edu:osuc_names:179750; scientificName: Oxyscelio naraws; **Location:** country: China; stateProvince: Zhejiang; locality: Tianmushan National Nature Reserve, Mt. Xianrending; verbatimElevation: 1200 m; locationRemarks: label transliteration: "Zhejiang, Tianmushan, Xianrending, 2011.07.25-29, Jin Chengyuan"; [浙江天目山仙人顶 1200 m, 30°20.56'N 119°26.03'E, 2011.07.25-29, YPT, 金程远]; verbatimCoordinates: 30°20.56'N 119°26.03'E; decimalLatitude: 30.3427; decimalLongitude: 119.4338; georeferenceProtocol: label; **Identification:** identifiedBy: Norman F. Johnson; dateIdentified: 2012; **Event:** eventID: urn:lsid:biosci.ohio-state.edu:osuc_occurrences:SCAU__2011000109; samplingProtocol: yellow pan trap; eventDate: 2011-07-25/29; **Record Level:** modified: 2013-07-17T11:03:40Z; language: en; collectionID: urn:lsid:biocol.org:col:34252; collectionCode: Insects; basisOfRecord: PreservedSpecimen; source: http://hol.osu.edu/spmInfo.html?id=SCAU%202011000109
- **Type status:** Other material. **Occurrence:** catalogNumber: SCAU 2011000108; recordedBy: Jin Cheng-Yuan; individualCount: 1; sex: male; lifeStage: adult; **Taxon:** taxonID: urn:lsid:biosci.ohio-state.edu:osuc_names:179750; scientificName: Oxyscelio naraws; **Location:** country: China; stateProvince: Zhejiang; locality: Tianmushan National Nature Reserve, Mt. Xianrending; verbatimElevation: 1200 m; locationRemarks: label transliteration: "Zhejiang, Tianmushan, Xianrending, 2011.07.25-29, Jin Chengyuan"; [浙江天目山仙人顶 1200 m, 30°20.56'N 119°26.03'E, 2011.07.25-29, YPT, 金程远]; verbatimCoordinates: 30°20.56'N 119°26.03'E; decimalLatitude: 30.3427; decimalLongitude: 119.4338; georeferenceProtocol: label; **Identification:** identifiedBy: Norman F. Johnson; dateIdentified: 2012; **Event:** eventID: urn:lsid:biosci.ohio-state.edu:osuc_occurrences:SCAU__2011000108; samplingProtocol: yellow pan trap; eventDate: 2011-07-25/29; **Record Level:** modified: 2013-07-17T11:03:40Z; language: en; collectionID: urn:lsid:biocol.org:col:34252; collectionCode: Insects; basisOfRecord: PreservedSpecimen; source: http://hol.osu.edu/spmInfo.html?id=SCAU%202011000108
- **Type status:** Other material. **Occurrence:** catalogNumber: SCAU 2011000107; recordedBy: Jin Cheng-Yuan; individualCount: 1; sex: male; lifeStage: adult; **Taxon:** taxonID: urn:lsid:biosci.ohio-state.edu:osuc_names:179750; scientificName: Oxyscelio naraws; **Location:** country: China; stateProvince: Zhejiang; locality: Tianmushan National Nature Reserve, Mt. Xianrending; verbatimElevation: 1200 m; locationRemarks: label transliteration: "Zhejiang, Tianmushan, Xianrending, 2011.07.25-29, Jin Chengyuan"; [浙江天目山仙人顶 1200 m, 30°20.56'N 119°26.03'E, 2011.07.25-29, YPT, 金程远]; verbatimCoordinates: 30°20.56'N 119°26.03'E; decimalLatitude: 30.3427; decimalLongitude: 119.4338; georeferenceProtocol: label; **Identification:** identifiedBy: Norman F. Johnson; dateIdentified: 2012; **Event:** eventID: urn:lsid:biosci.ohio-state.edu:osuc_occurrences:SCAU__2011000107; samplingProtocol: yellow pan trap; eventDate: 2011-07-25/29; **Record Level:** modified: 2013-07-17T11:03:39Z; language: en; collectionID: urn:lsid:biocol.org:col:34252; collectionCode: Insects; basisOfRecord: PreservedSpecimen; source: http://hol.osu.edu/spmInfo.html?id=SCAU%202011000107
- **Type status:** Other material. **Occurrence:** catalogNumber: SCAU 2011000048; recordedBy: Jin Cheng-Yuan; individualCount: 1; sex: female; lifeStage: adult; **Taxon:** taxonID: urn:lsid:biosci.ohio-state.edu:osuc_names:179750; scientificName: Oxyscelio naraws; **Location:** country: China; stateProvince: Zhejiang; locality: Tianmushan National Nature Reserve, Mt. Xianrending; verbatimElevation: 1200 m; locationRemarks: label transliteration: "Zhejiang, Tianmushan, Xianrending, 2011.07.25-29, Jin Chengyuan"; [浙江天目山仙人顶 1200 m, 30°20.56'N 119°26.03'E, 2011.07.25-29, sweeping, 金程远]; verbatimCoordinates: 30°20.56'N 119°26.03'E; decimalLatitude: 30.3427; decimalLongitude: 119.4338; georeferenceProtocol: label; **Identification:** identifiedBy: Norman F. Johnson; dateIdentified: 2012; **Event:** eventID: urn:lsid:biosci.ohio-state.edu:osuc_occurrences:SCAU__2011000048; samplingProtocol: sweeping; eventDate: 2011-07-25/29; **Record Level:** modified: 2013-07-17T11:03:28Z; language: en; collectionID: urn:lsid:biocol.org:col:34252; collectionCode: Insects; basisOfRecord: PreservedSpecimen; source: http://hol.osu.edu/spmInfo.html?id=SCAU%202011000048
- **Type status:** Other material. **Occurrence:** catalogNumber: SCAU 2011000106; recordedBy: Jin Cheng-Yuan; individualCount: 1; sex: male; lifeStage: adult; **Taxon:** taxonID: urn:lsid:biosci.ohio-state.edu:osuc_names:179750; scientificName: Oxyscelio naraws; **Location:** country: China; stateProvince: Zhejiang; locality: Tianmushan National Nature Reserve, Mt. Xianrending; verbatimElevation: 1200 m; locationRemarks: label transliteration: "Zhejiang, Tianmushan, Xianrending, 2011.07.25-29, Jin Chengyuan"; [浙江天目山仙人顶 1200 m, 30°20.56'N 119°26.03'E, 2011.07.25-29, YPT, 金程远]; verbatimCoordinates: 30°20.56'N 119°26.03'E; decimalLatitude: 30.3427; decimalLongitude: 119.4338; georeferenceProtocol: label; **Identification:** identifiedBy: Norman F. Johnson; dateIdentified: 2012; **Event:** eventID: urn:lsid:biosci.ohio-state.edu:osuc_occurrences:SCAU__2011000106; samplingProtocol: yellow pan trap; eventDate: 2011-07-25/29; **Record Level:** modified: 2013-07-17T11:03:39Z; language: en; collectionID: urn:lsid:biocol.org:col:34252; collectionCode: Insects; basisOfRecord: PreservedSpecimen; source: http://hol.osu.edu/spmInfo.html?id=SCAU%202011000106
- **Type status:** Other material. **Occurrence:** catalogNumber: SCAU 2011000105; recordedBy: Jin Cheng-Yuan; individualCount: 1; sex: male; lifeStage: adult; **Taxon:** taxonID: urn:lsid:biosci.ohio-state.edu:osuc_names:179750; scientificName: Oxyscelio naraws; **Location:** country: China; stateProvince: Zhejiang; locality: Tianmushan National Nature Reserve, Mt. Xianrending; verbatimElevation: 1200 m; locationRemarks: label transliteration: "Zhejiang, Tianmushan, Xianrending, 2011.07.25-29, Jin Chengyuan"; [浙江天目山仙人顶 1200 m, 30°20.56'N 119°26.03'E, 2011.07.25-29, YPT, 金程远]; verbatimCoordinates: 30°20.56'N 119°26.03'E; decimalLatitude: 30.3427; decimalLongitude: 119.4338; georeferenceProtocol: label; **Identification:** identifiedBy: Norman F. Johnson; dateIdentified: 2012; **Event:** eventID: urn:lsid:biosci.ohio-state.edu:osuc_occurrences:SCAU__2011000105; samplingProtocol: yellow pan trap; eventDate: 2011-07-25/29; **Record Level:** modified: 2013-07-17T11:03:39Z; language: en; collectionID: urn:lsid:biocol.org:col:34252; collectionCode: Insects; basisOfRecord: PreservedSpecimen; source: http://hol.osu.edu/spmInfo.html?id=SCAU%202011000105
- **Type status:** Other material. **Occurrence:** catalogNumber: SCAU 2011000101; recordedBy: Jin Cheng-Yuan; individualCount: 1; sex: female; lifeStage: adult; **Taxon:** taxonID: urn:lsid:biosci.ohio-state.edu:osuc_names:179750; scientificName: Oxyscelio naraws; **Location:** country: China; stateProvince: Zhejiang; locality: Tianmushan National Nature Reserve, Mt. Xianrending; verbatimElevation: 1200 m; locationRemarks: label transliteration: "Zhejiang, Tianmushan, Xianrending, 2011.07.25-29, Jin Chengyuan"; [浙江天目山仙人顶 1200 m, 30°20.56'N 119°26.03'E, 2011.07.25-29, YPT, 金程远]; verbatimCoordinates: 30°20.56'N 119°26.03'E; decimalLatitude: 30.3427; decimalLongitude: 119.4338; georeferenceProtocol: label; **Identification:** identifiedBy: Norman F. Johnson; dateIdentified: 2012; **Event:** eventID: urn:lsid:biosci.ohio-state.edu:osuc_occurrences:SCAU__2011000101; samplingProtocol: yellow pan trap; eventDate: 2011-07-25/29; **Record Level:** modified: 2013-07-17T11:03:38Z; language: en; collectionID: urn:lsid:biocol.org:col:34252; collectionCode: Insects; basisOfRecord: PreservedSpecimen; source: http://hol.osu.edu/spmInfo.html?id=SCAU%202011000101
- **Type status:** Other material. **Occurrence:** catalogNumber: SCAU 2011000100; recordedBy: Jin Cheng-Yuan; individualCount: 1; sex: female; lifeStage: adult; **Taxon:** taxonID: urn:lsid:biosci.ohio-state.edu:osuc_names:179750; scientificName: Oxyscelio naraws; **Location:** country: China; stateProvince: Zhejiang; locality: Tianmushan National Nature Reserve, Mt. Xianrending; verbatimElevation: 1200 m; locationRemarks: label transliteration: "Zhejiang, Tianmushan, Xianrending, 2011.07.25-29, Jin Chengyuan"; [浙江天目山仙人顶 1200 m, 30°20.56'N 119°26.03'E, 2011.07.25-29, YPT, 金程远]; verbatimCoordinates: 30°20.56'N 119°26.03'E; decimalLatitude: 30.3427; decimalLongitude: 119.4338; georeferenceProtocol: label; **Identification:** identifiedBy: Norman F. Johnson; dateIdentified: 2012; **Event:** eventID: urn:lsid:biosci.ohio-state.edu:osuc_occurrences:SCAU__2011000100; samplingProtocol: yellow pan trap; eventDate: 2011-07-25/29; **Record Level:** modified: 2013-07-17T11:03:38Z; language: en; collectionID: urn:lsid:biocol.org:col:34252; collectionCode: Insects; basisOfRecord: PreservedSpecimen; source: http://hol.osu.edu/spmInfo.html?id=SCAU%202011000100

#### Distribution

This species was originally described by Kozlov and Lê from Vietname (Lê 2000), and Burks et al. 2013 extended its known range to include Thailand, Indonesia, Malaysia and Brunei. These records extend the range to the north to include Guangdong and Zhejiang Provinces. Link to dynamic distribution map: http://hol.osu.edu/map-large.html?id=179750

### Oxyscelio perpensus

Kononova, 2007

http://lsid.tdwg.org/urn:lsid:biosci.ohio-state.edu:osuc_concepts:243849

urn:lsid:zoobank.org:act:8323BFBD-BFC7-4DA7-9C8A-767CECA029EC

http://species-id.net/wiki/Oxyscelio_perpensus

Oxyscelio perpensum Kononova and Fursov 2007: 63. Original description.

#### Materials

- **Type status:** Other material. **Occurrence:** catalogNumber: SCAU 2011000418; recordedBy: Shi Min; individualCount: 1; sex: female; lifeStage: adult; **Taxon:** taxonID: urn:lsid:biosci.ohio-state.edu:osuc_names:243849; scientificName: Oxyscelio perpensus; **Location:** country: China; stateProvince: Hebei; county: Zhangjiakou; locality: Mt Dongling; locationRemarks: label transliteration: "Hebei, Zhang jia kou, Donglingshan, 2005.08.11, Shi Min"; [河北张家口东灵山, 2005.08.11, 时敏]; decimalLatitude: 40.0333; decimalLongitude: 115.45; georeferenceProtocol: GPS; **Identification:** identifiedBy: Norman F. Johnson; dateIdentified: 2012; **Event:** eventID: urn:lsid:biosci.ohio-state.edu:osuc_occurrences:SCAU__2011000418; samplingProtocol: none specified; eventDate: 2005-08-11; **Record Level:** modified: 2013-07-17T11:03:50Z; language: en; collectionID: urn:lsid:biocol.org:col:34252; collectionCode: Insects; basisOfRecord: PreservedSpecimen; source: http://hol.osu.edu/spmInfo.html?id=SCAU%202011000418
- **Type status:** Other material. **Occurrence:** catalogNumber: SCAU 2011000419; recordedBy: Shi Min; individualCount: 1; sex: female; lifeStage: adult; **Taxon:** taxonID: urn:lsid:biosci.ohio-state.edu:osuc_names:243849; scientificName: Oxyscelio perpensus; **Location:** country: China; stateProvince: Hebei; county: Zhangjiakou; locality: Mt Dongling; locationRemarks: label transliteration: "Hebei, Zhang jia kou, Donglingshan, 2005.08.11, Shi Min"; [河北张家口东灵山, 2005.08.11, 时敏]; decimalLatitude: 40.0333; decimalLongitude: 115.45; georeferenceProtocol: GPS; **Identification:** identifiedBy: Norman F. Johnson; dateIdentified: 2012; **Event:** eventID: urn:lsid:biosci.ohio-state.edu:osuc_occurrences:SCAU__2011000419; samplingProtocol: none specified; eventDate: 2005-08-11; **Record Level:** modified: 2013-07-17T11:03:50Z; language: en; collectionID: urn:lsid:biocol.org:col:34252; collectionCode: Insects; basisOfRecord: PreservedSpecimen; source: http://hol.osu.edu/spmInfo.html?id=SCAU%202011000419
- **Type status:** Other material. **Occurrence:** catalogNumber: SCAU 2011000672; recordedBy: Shi Min; individualCount: 1; sex: female; lifeStage: adult; **Taxon:** taxonID: urn:lsid:biosci.ohio-state.edu:osuc_names:243849; scientificName: Oxyscelio perpensus; **Location:** country: China; stateProvince: Hebei; county: Zhangjiakou; locality: Mt Dongling; locationRemarks: label transliteration: "Hebei, Zhang jia kou, Donglingshan, 2005.08.11, Shi Min"; [河北张家口东灵山, 2005.08.11, 时敏]; decimalLatitude: 40.0333; decimalLongitude: 115.45; georeferenceProtocol: GPS; **Identification:** identifiedBy: Norman F. Johnson; dateIdentified: 2012; **Event:** eventID: urn:lsid:biosci.ohio-state.edu:osuc_occurrences:SCAU__2011000672; samplingProtocol: none specified; eventDate: 2005-08-11; **Record Level:** modified: 2013-07-17T11:04:02Z; language: en; collectionID: urn:lsid:biocol.org:col:34252; collectionCode: Insects; basisOfRecord: PreservedSpecimen; source: http://hol.osu.edu/spmInfo.html?id=SCAU%202011000672
- **Type status:** Other material. **Occurrence:** catalogNumber: SCAU 2011000072; recordedBy: Chen Hua-Yan; individualCount: 1; sex: male; lifeStage: adult; **Taxon:** taxonID: urn:lsid:biosci.ohio-state.edu:osuc_names:243849; scientificName: Oxyscelio perpensus; **Location:** country: China; stateProvince: Zhejiang; locality: Tianmushan National Nature Reserve, Mt. Xianrending; verbatimElevation: 1200 m; locationRemarks: label transliteration: "Zhejiang, Tianmushan, Xianrending, 2011.07.25-29, Chen Huayan"; [浙江天目山仙人顶 1200 m, 30°20.56'N 119°26.03'E, 2011.07.25-29, sweeping, 陈华燕]; verbatimCoordinates: 30°20.56'N 119°26.03'E; decimalLatitude: 30.3427; decimalLongitude: 119.4338; georeferenceProtocol: label; **Identification:** identifiedBy: Norman F. Johnson; dateIdentified: 2012; **Event:** eventID: urn:lsid:biosci.ohio-state.edu:osuc_occurrences:SCAU__2011000072; samplingProtocol: sweeping; eventDate: 2011-07-25/29; **Record Level:** modified: 2013-07-17T11:03:30Z; language: en; collectionID: urn:lsid:biocol.org:col:34252; collectionCode: Insects; basisOfRecord: PreservedSpecimen; source: http://hol.osu.edu/spmInfo.html?id=SCAU%202011000072

#### Distribution

This species was known only from Japan. Its distribution in China extends from Hebei in the north to Zhejiang in the east. Link to dynamic distribution map: http://hol.osu.edu/map-large.html?id=243849

### Oxyscelio striarum

Burks, 2013

http://lsid.tdwg.org/urn:lsid:biosci.ohio-state.edu:osuc_concepts:275511

urn:lsid:zoobank.org:act:DAE4E3B3-8F03-4E08-88F1-DD684EE132A1

http://species-id.net/wiki/Oxyscelio_striarum

Oxyscelio striarum Burks et al. 2013: 18, 27, 233. Original description, keyed, placed in *striarum* species group.

#### Materials

- **Type status:** Other material. **Occurrence:** catalogNumber: SCAU 201010064; recordedBy: Chen Hua-Yan; individualCount: 1; sex: female; lifeStage: adult; **Taxon:** taxonID: urn:lsid:biosci.ohio-state.edu:osuc_names:275511; scientificName: Oxyscelio striarum; **Location:** country: China; stateProvince: Hainan; locality: Mt Yinggeling; locationRemarks: label transliteration: "Hainan, Yinggeshan, 2008.11.16-20, Chen Huayan"; [海南鹦哥岭, 2008.11.16-20, 陈华燕]; decimalLatitude: 18.8167; decimalLongitude: 109.1833; georeferenceProtocol: GPS; **Identification:** identifiedBy: Norman F. Johnson; dateIdentified: 2012; **Event:** eventID: urn:lsid:biosci.ohio-state.edu:osuc_occurrences:SCAU__201010064; samplingProtocol: none specified; eventDate: 2008-11-16/20; **Record Level:** modified: 2013-07-17T11:03:26Z; language: en; collectionID: urn:lsid:biocol.org:col:34252; collectionCode: Insects; basisOfRecord: PreservedSpecimen; source: http://hol.osu.edu/spmInfo.html?id=SCAU%20201010064
- **Type status:** Other material. **Occurrence:** catalogNumber: SCAU 2011000038; recordedBy: Wang Man-Man; individualCount: 1; sex: female; lifeStage: adult; **Taxon:** taxonID: urn:lsid:biosci.ohio-state.edu:osuc_names:275511; scientificName: Oxyscelio striarum; **Location:** country: China; stateProvince: Hainan; locality: Bawangling National Nature Reserve; locationRemarks: label transliteration: "Hainan, Bawangling, 2008.11.26, Wang Manman"; [海南霸王岭, 2008.11.26, 王漫漫]; decimalLatitude: 19.1167; decimalLongitude: 109.05; georeferenceProtocol: GPS; **Identification:** identifiedBy: Norman F. Johnson; dateIdentified: 2012; **Event:** eventID: urn:lsid:biosci.ohio-state.edu:osuc_occurrences:SCAU__2011000038; samplingProtocol: none specified; eventDate: 2008-11-26; **Record Level:** modified: 2013-07-17T11:03:27Z; language: en; collectionID: urn:lsid:biocol.org:col:34252; collectionCode: Insects; basisOfRecord: PreservedSpecimen; source: http://hol.osu.edu/spmInfo.html?id=SCAU%202011000038

#### Distribution

This is a southeast Asian species, known to extend from Thailand east to Borneo (Brunei, Sabah, West Kalimantan). It is also found in the southernmost part of China, Hainan. Link to dynamic distribution map: http://hol.osu.edu/map-large.html?id=275511

### Heptascelio hamatus

Masner & Johnson, 2008

http://lsid.tdwg.org/urn:lsid:biosci.ohio-state.edu:osuc_concepts:223416

urn:lsid:zoobank.org:act:950F63A6-7258-448D-8905-6A7A9C4594D6

Heptascelio hamatus Johnson et al. 2008: 8, 17. Original description, keyed.

#### Materials

- **Type status:** Other material. **Occurrence:** catalogNumber: SCAU 200705955; recordedBy: Weng Li-Qiong; individualCount: 1; sex: female; lifeStage: adult; **Taxon:** taxonID: urn:lsid:biosci.ohio-state.edu:osuc_names:223416; scientificName: Heptascelio hamatus; **Location:** country: China; stateProvince: Hainan; locality: Wuzhishan National Nature Reserve; locationRemarks: label transliteration: "Hainan, Wuzhishan, Shuiman, 2007.5.16-18, Weng Liqiong"; [海南五指山水满, 2007.5.16-18, 翁丽琼]; decimalLatitude: 18.85; decimalLongitude: 109.65; georeferenceProtocol: GPS; **Identification:** identifiedBy: Norman F. Johnson; dateIdentified: 2012; **Event:** eventID: urn:lsid:biosci.ohio-state.edu:osuc_occurrences:SCAU__200705955; samplingProtocol: none specified; eventDate: 2007-05-16/18; habitat: waterfall; **Record Level:** modified: 2013-07-17T11:03:12Z; language: en; collectionID: urn:lsid:biocol.org:col:34252; collectionCode: Insects; basisOfRecord: PreservedSpecimen; source: http://hol.osu.edu/spmInfo.html?id=SCAU%20200705955
- **Type status:** Other material. **Occurrence:** catalogNumber: SCAU 200705954; recordedBy: Weng Li-Qiong; individualCount: 1; sex: female; lifeStage: adult; **Taxon:** taxonID: urn:lsid:biosci.ohio-state.edu:osuc_names:223416; scientificName: Heptascelio hamatus; **Location:** country: China; stateProvince: Hainan; locality: Wuzhishan National Nature Reserve; locationRemarks: label transliteration: "Hainan, Wuzhishan, Shuiman, 2007.5.16-18, Weng Liqiong"; [海南五指山水满, 2007.5.16-18, 翁丽琼]; decimalLatitude: 18.85; decimalLongitude: 109.65; georeferenceProtocol: GPS; **Identification:** identifiedBy: Norman F. Johnson; dateIdentified: 2012; **Event:** eventID: urn:lsid:biosci.ohio-state.edu:osuc_occurrences:SCAU__200705954; samplingProtocol: none specified; eventDate: 2007-05-16/18; habitat: waterfall; **Record Level:** modified: 2013-07-17T11:03:12Z; language: en; collectionID: urn:lsid:biocol.org:col:34252; collectionCode: Insects; basisOfRecord: PreservedSpecimen; source: http://hol.osu.edu/spmInfo.html?id=SCAU%20200705954
- **Type status:** Other material. **Occurrence:** catalogNumber: SCAU 200705947; recordedBy: Weng Li-Qiong; individualCount: 1; sex: female; lifeStage: adult; **Taxon:** taxonID: urn:lsid:biosci.ohio-state.edu:osuc_names:223416; scientificName: Heptascelio hamatus; **Location:** country: China; stateProvince: Hainan; locality: Wuzhishan National Nature Reserve; locationRemarks: label transliteration: "Hainan, Wuzhishan, Shuiman, 2007.5.16-18, Weng Liqiong"; [海南五指山水满, 2007.5.16-18, 翁丽琼]; decimalLatitude: 18.85; decimalLongitude: 109.65; georeferenceProtocol: GPS; **Identification:** identifiedBy: Norman F. Johnson; dateIdentified: 2012; **Event:** eventID: urn:lsid:biosci.ohio-state.edu:osuc_occurrences:SCAU__200705947; samplingProtocol: none specified; eventDate: 2007-05-16/18; habitat: waterfall; **Record Level:** modified: 2013-07-17T11:03:11Z; language: en; collectionID: urn:lsid:biocol.org:col:34252; collectionCode: Insects; basisOfRecord: PreservedSpecimen; source: http://hol.osu.edu/spmInfo.html?id=SCAU%20200705947
- **Type status:** Other material. **Occurrence:** catalogNumber: SCAU 2011000599; recordedBy: Xu Zai-Fu; individualCount: 1; sex: female; lifeStage: adult; **Taxon:** taxonID: urn:lsid:biosci.ohio-state.edu:osuc_names:223416; scientificName: Heptascelio hamatus; **Location:** country: China; stateProvince: Guangdong; locality: Nanling National Nature Reserve; locationRemarks: label transliteration: "Guangdong, Nanling Nature Reserve, 2011.09.10-12, YPT, Xu Zaifu"; [广东南岭自然保护区, 2011.09.10-12, YPT, 许再福]; decimalLatitude: 24.9; decimalLongitude: 113; georeferenceProtocol: GPS; **Identification:** identifiedBy: Norman F. Johnson; dateIdentified: 2012; **Event:** eventID: urn:lsid:biosci.ohio-state.edu:osuc_occurrences:SCAU__2011000599; samplingProtocol: yellow pan trap; eventDate: 2011-09-10/12; **Record Level:** modified: 2013-07-17T11:03:56Z; language: en; collectionID: urn:lsid:biocol.org:col:34252; collectionCode: Insects; basisOfRecord: PreservedSpecimen; source: http://hol.osu.edu/spmInfo.html?id=SCAU%202011000599
- **Type status:** Other material. **Occurrence:** catalogNumber: SCAU 2011000598; recordedBy: Xu Zai-Fu; individualCount: 1; sex: female; lifeStage: adult; **Taxon:** taxonID: urn:lsid:biosci.ohio-state.edu:osuc_names:223416; scientificName: Heptascelio hamatus; **Location:** country: China; stateProvince: Guangdong; locality: Nanling National Nature Reserve; locationRemarks: label transliteration: "Guangdong, Nanling Nature Reserve, 2011.09.10-12, YPT, Xu Zaifu"; [广东南岭自然保护区, 2011.09.10-12, YPT, 许再福]; decimalLatitude: 24.9; decimalLongitude: 113; georeferenceProtocol: GPS; **Identification:** identifiedBy: Norman F. Johnson; dateIdentified: 2012; **Event:** eventID: urn:lsid:biosci.ohio-state.edu:osuc_occurrences:SCAU__2011000598; samplingProtocol: yellow pan trap; eventDate: 2011-09-10/12; **Record Level:** modified: 2013-07-17T11:03:56Z; language: en; collectionID: urn:lsid:biocol.org:col:34252; collectionCode: Insects; basisOfRecord: PreservedSpecimen; source: http://hol.osu.edu/spmInfo.html?id=SCAU%202011000598
- **Type status:** Other material. **Occurrence:** catalogNumber: SCAU 2011000595; recordedBy: Xu Zai-Fu; individualCount: 1; sex: female; lifeStage: adult; **Taxon:** taxonID: urn:lsid:biosci.ohio-state.edu:osuc_names:223416; scientificName: Heptascelio hamatus; **Location:** country: China; stateProvince: Guangdong; locality: Nanling National Nature Reserve; locationRemarks: label transliteration: "Guangdong, Nanling Nature Reserve, 2011.09.10-12, YPT, Xu Zaifu"; [广东南岭自然保护区, 2011.09.10-12, YPT, 许再福]; decimalLatitude: 24.9; decimalLongitude: 113; georeferenceProtocol: GPS; **Identification:** identifiedBy: Norman F. Johnson; dateIdentified: 2012; **Event:** eventID: urn:lsid:biosci.ohio-state.edu:osuc_occurrences:SCAU__2011000595; samplingProtocol: yellow pan trap; eventDate: 2011-09-10/12; **Record Level:** modified: 2013-07-17T11:03:55Z; language: en; collectionID: urn:lsid:biocol.org:col:34252; collectionCode: Insects; basisOfRecord: PreservedSpecimen; source: http://hol.osu.edu/spmInfo.html?id=SCAU%202011000595
- **Type status:** Other material. **Occurrence:** catalogNumber: SCAU 2011000596; recordedBy: Xu Zai-Fu; individualCount: 1; sex: female; lifeStage: adult; **Taxon:** taxonID: urn:lsid:biosci.ohio-state.edu:osuc_names:223416; scientificName: Heptascelio hamatus; **Location:** country: China; stateProvince: Guangdong; locality: Nanling National Nature Reserve; locationRemarks: label transliteration: "Guangdong, Nanling Nature Reserve, 2011.09.10-12, YPT, Xu Zaifu"; [广东南岭自然保护区, 2011.09.10-12, YPT, 许再福]; decimalLatitude: 24.9; decimalLongitude: 113; georeferenceProtocol: GPS; **Identification:** identifiedBy: Norman F. Johnson; dateIdentified: 2012; **Event:** eventID: urn:lsid:biosci.ohio-state.edu:osuc_occurrences:SCAU__2011000596; samplingProtocol: yellow pan trap; eventDate: 2011-09-10/12; **Record Level:** modified: 2013-07-17T11:03:56Z; language: en; collectionID: urn:lsid:biocol.org:col:34252; collectionCode: Insects; basisOfRecord: PreservedSpecimen; source: http://hol.osu.edu/spmInfo.html?id=SCAU%202011000596
- **Type status:** Other material. **Occurrence:** catalogNumber: SCAU 2011000597; recordedBy: Xu Zai-Fu; individualCount: 1; sex: female; lifeStage: adult; **Taxon:** taxonID: urn:lsid:biosci.ohio-state.edu:osuc_names:223416; scientificName: Heptascelio hamatus; **Location:** country: China; stateProvince: Guangdong; locality: Nanling National Nature Reserve; locationRemarks: label transliteration: "Guangdong, Nanling Nature Reserve, 2011.09.10-12, YPT, Xu Zaifu"; [广东南岭自然保护区, 2011.09.10-12, YPT, 许再福]; decimalLatitude: 24.9; decimalLongitude: 113; georeferenceProtocol: GPS; **Identification:** identifiedBy: Norman F. Johnson; dateIdentified: 2012; **Event:** eventID: urn:lsid:biosci.ohio-state.edu:osuc_occurrences:SCAU__2011000597; samplingProtocol: yellow pan trap; eventDate: 2011-09-10/12; **Record Level:** modified: 2013-07-17T11:03:56Z; language: en; collectionID: urn:lsid:biocol.org:col:34252; collectionCode: Insects; basisOfRecord: PreservedSpecimen; source: http://hol.osu.edu/spmInfo.html?id=SCAU%202011000597

#### Distribution

*Heptascelio hamatus* is the most common species of the genus in Asia and is widespread from Thailand to Sulawesi and north to Taiwan. On mainland China it has been rarely collected and is known only from Hainan and Guangdong. Link to dynamic distribution map: http://hol.osu.edu/map-large.html?id=223416

### Platyscelio pulchricornis

Kieffer, 1905

http://lsid.tdwg.org/urn:lsid:biosci.ohio-state.edu:osuc_concepts:5091

urn:lsid:zoobank.org:act:7290F370-068F-458B-86BB-22CFEB2C2926

Platyscelio pulchricornis Kieffer 1905: 13. Orlginal description.
Platyscelio abnormis Crawford 1910: 126. Original description.
Platyscelio mirabilis Dodd 1913: 132. Original description.
Platyscelio punctatus Kieffer 1913:321. Original description.
Platyscelio wilcoxi Fullaway 1913: 283. Original description.
Platyscelio dunensis Mukerjee 1993: 78. Original description.

#### Materials

- **Type status:** Other material. **Occurrence:** catalogNumber: OSUC 207884; recordedBy: D. Munroe; individualCount: 1; sex: male; lifeStage: adult; **Taxon:** taxonID: urn:lsid:biosci.ohio-state.edu:osuc_names:5091; scientificName: Platyscelio pulchricornis; **Location:** country: Malaysia; stateProvince: Sarawak; county: Sibu; locality: Nanga Ngungun, NF-2-13, Ngemah River; locationRemarks: [MALAYSIA: Sarawak 3rd division, Sg. Ngemah, Ng. Ngungun 10.I.1975, D. Munroe NF-2-13]; decimalLatitude: 1.9886; decimalLongitude: 112.3994; georeferenceProtocol: WikiMapia; georeferenceRemarks: Third Division is a former name of Sibu Dist.; **Identification:** identifiedBy: Norman F. Johnson; dateIdentified: 2008; **Event:** eventID: urn:lsid:biosci.ohio-state.edu:osuc_occurrences:OSUC__207884; samplingProtocol: none specified; eventDate: 1975-01-10; **Record Level:** modified: 2013-01-30T10:13:21Z; language: en; collectionID: urn:lsid:biocol.org:col:34252; collectionCode: Insects; basisOfRecord: PreservedSpecimen; source: http://hol.osu.edu/spmInfo.html?id=OSUC%20207884
- **Type status:** Other material. **Occurrence:** catalogNumber: OSUC 207891; recordedBy: Roy Snelling; individualCount: 1; sex: female; lifeStage: adult; **Taxon:** taxonID: urn:lsid:biosci.ohio-state.edu:osuc_names:5091; scientificName: Platyscelio pulchricornis; **Location:** country: Thailand; stateProvince: Chiang Mai; locality: Amphur Mae; verbatimElevation: 250 m; locationRemarks: [THAILAND: Ch.Mai Amphur Mae 250m. 15°42'N 98°49'E 1-31.i.1998, MT Roy Snelling ]; verbatimCoordinates: 15°42'N 98°49'E; decimalLatitude: 15.7; decimalLongitude: 98.8167; georeferenceProtocol: label; **Identification:** identifiedBy: Norman F. Johnson; dateIdentified: 2008; **Event:** eventID: urn:lsid:biosci.ohio-state.edu:osuc_occurrences:OSUC__207891; samplingProtocol: malaise trap; eventDate: 1998-01-01/31; **Record Level:** modified: 2013-03-31T08:00:12Z; language: en; collectionID: urn:lsid:biocol.org:col:34252; collectionCode: Insects; basisOfRecord: PreservedSpecimen; source: http://hol.osu.edu/spmInfo.html?id=OSUC%20207891
- **Type status:** Other material. **Occurrence:** catalogNumber: SCAU 2010100196; recordedBy: Xu Zai-Fu; individualCount: 1; sex: female; lifeStage: adult; **Taxon:** taxonID: urn:lsid:biosci.ohio-state.edu:osuc_names:5091; scientificName: Platyscelio pulchricornis; **Location:** country: China; stateProvince: Guangdong; county: Guangzhou; locality: Tianlu Lake; locationRemarks: scutellar line & propodeum like africanus; label transliteration: "[Guangdong] Guangzhou, Tianluhu, 2009.V.16, Xu Zaifu"; [广州天麓湖, 2009.V.16, 许再福]; decimalLatitude: 23.2167; decimalLongitude: 113.4167; georeferenceProtocol: GPS; **Identification:** identifiedBy: Norman F. Johnson; dateIdentified: 2012; **Event:** eventID: urn:lsid:biosci.ohio-state.edu:osuc_occurrences:SCAU__2010100196; samplingProtocol: none specified; eventDate: 2009-05-16; **Record Level:** modified: 2013-07-17T11:03:24Z; language: en; collectionID: urn:lsid:biocol.org:col:34252; collectionCode: Insects; basisOfRecord: PreservedSpecimen; source: http://hol.osu.edu/spmInfo.html?id=SCAU%202010100196
- **Type status:** Other material. **Occurrence:** catalogNumber: SCAU 201010003; recordedBy: Chen Hua-Yan; individualCount: 1; sex: male; lifeStage: adult; **Taxon:** taxonID: urn:lsid:biosci.ohio-state.edu:osuc_names:5091; scientificName: Platyscelio pulchricornis; **Location:** country: China; stateProvince: Hainan; locality: Mt Yinggeling; locationRemarks: label transliteration: "Hainan, Yinggeling, 2010.07.17-20, Chen Huayan"; [海南鹦哥岭, 2010.07.17-20, 陈华燕]; decimalLatitude: 18.8167; decimalLongitude: 109.1833; georeferenceProtocol: GPS; **Identification:** identifiedBy: Norman F. Johnson; dateIdentified: 2012; **Event:** eventID: urn:lsid:biosci.ohio-state.edu:osuc_occurrences:SCAU__201010003; samplingProtocol: none specified; eventDate: 2010-07-17/20; **Record Level:** modified: 2013-07-17T11:03:23Z; language: en; collectionID: urn:lsid:biocol.org:col:34252; collectionCode: Insects; basisOfRecord: PreservedSpecimen; source: http://hol.osu.edu/spmInfo.html?id=SCAU%20201010003
- **Type status:** Other material. **Occurrence:** catalogNumber: SCAU 2010100037; recordedBy: Chen Hua-Yan; individualCount: 1; sex: male; lifeStage: adult; **Taxon:** taxonID: urn:lsid:biosci.ohio-state.edu:osuc_names:5091; scientificName: Platyscelio pulchricornis; **Location:** country: China; stateProvince: Hainan; locality: Mt Yinggeling; locationRemarks: label transliteration: "Hainan, Yinggeling, 2010.07.17-20, Chen Huayan"; [海南鹦哥岭, 2010.07.17-20, 陈华燕]; decimalLatitude: 18.8167; decimalLongitude: 109.1833; georeferenceProtocol: GPS; **Identification:** identifiedBy: Norman F. Johnson; dateIdentified: 2012; **Event:** eventID: urn:lsid:biosci.ohio-state.edu:osuc_occurrences:SCAU__2010100037; samplingProtocol: none specified; eventDate: 2010-07-17/20; **Record Level:** modified: 2013-07-17T11:03:23Z; language: en; collectionID: urn:lsid:biocol.org:col:34252; collectionCode: Insects; basisOfRecord: PreservedSpecimen; source: http://hol.osu.edu/spmInfo.html?id=SCAU%202010100037

#### Distribution

*Platyscelio pulchricornis* is very widely distributed, usually in lowland tropics from India to Australia, Guam and Vanuatu, but also extending north into Nepal and Japan. In China it has been recorded in the southern part of the country only, in Guangdong and Hainan. Link to dynamic distribution map: http://hol.osu.edu/map-large.html?id=5091

## Supplementary Material

XML Treatment for Oxyscelio arvi

XML Treatment for Oxyscelio ceylonensis

XML Treatment for Oxyscelio convergens

XML Treatment for Oxyscelio cordis

XML Treatment for Oxyscelio crebritas

XML Treatment for Oxyscelio cuculli

XML Treatment for Oxyscelio dermatoglyphes

XML Treatment for Oxyscelio doumao

XML Treatment for Oxyscelio florus

XML Treatment for Oxyscelio granorum

XML Treatment for Oxyscelio intermedietas

XML Treatment for Oxyscelio jugi

XML Treatment for Oxyscelio kramatos

XML Treatment for Oxyscelio longiventris

XML Treatment for Oxyscelio naraws

XML Treatment for Oxyscelio perpensus

XML Treatment for Oxyscelio striarum

XML Treatment for Heptascelio hamatus

XML Treatment for Platyscelio pulchricornis

## Supplementary files

### DarwinCore Archive files of Chinese Oxyscelio, Heptascelio, Platyscelio

**Authors:** Norman F. Johnson, Roger D. Burks, Andrew D. Austin, Xu Zaifu **Data type:** occurrences DarwinCore Archive files for data reported, in zipped format. **File name:** ChineseOxyscelio.zip
